# Miniaturized Modular Click Chemistry‐enabled Rapid Discovery of Unique SARS‐CoV‐2 M^pro^ Inhibitors With Robust Potency and Drug‐like Profile

**DOI:** 10.1002/advs.202404884

**Published:** 2024-09-25

**Authors:** Mianling Yang, Myoung Kyu Lee, Shenghua Gao, Letian Song, Hye‐Yeon Jang, Inseong Jo, Chun‐Chiao Yang, Katharina Sylvester, Chunkyu Ko, Shuo Wang, Bing Ye, Kai Tang, Junyi Li, Manyu Gu, Christa E. Müller, Norbert Sträter, Xinyong Liu, Meehyein Kim, Peng Zhan

**Affiliations:** ^1^ Department of Medicinal Chemistry Key Laboratory of Chemical Biology Ministry of Education School of Pharmaceutical Sciences Shandong University Ji'nan 250012 China; ^2^ Infectious Diseases Therapeutic Research Center Korea Research Institute of Chemical Technology (KRICT) Daejeon 34114 Republic of Korea; ^3^ PharmaCenter Bonn & Pharmaceutical Institute Department of Pharmaceutical & Medicinal Chemistry University of Bonn An der Immenburg 4 53113 Bonn Germany; ^4^ Institute of Bioanalytical Chemistry Leipzig University Deutscher Platz 5 04103 Leipzig Germany

**Keywords:** click chemistry, direct screening, main protease, miniaturized synthesis, non‐covalent inhibitors, SARS‐CoV‐2

## Abstract

The COVID‐19 pandemic has required an expeditious advancement of innovative antiviral drugs. In this study, focused compound libraries are synthesized in 96‐ well plates utilizing modular click chemistry to rapidly discover potent inhibitors targeting the main protease (M^pro^) of SARS‐CoV‐2. Subsequent direct biological screening identifies novel 1,2,3‐triazole derivatives as robust M^pro^ inhibitors with high anti‐SARS‐CoV‐2 activity. Notably, **C5N17B** demonstrates sub‐micromolar M^pro^ inhibitory potency (IC_50_ = 0.12 µM) and excellent antiviral activity in Calu‐3 cells determined in an immunofluorescence‐based antiviral assay (EC_50_ = 0.078 µM, no cytotoxicity: CC_50_ > 100 µM). **C5N17B** shows superior potency to nirmatrelvir (EC_50_ = 1.95 µM) and similar efficacy to ensitrelvir (EC_50_ = 0.11 µM). Importantly, this compound displays high antiviral activities against several SARS‐CoV‐2 variants (Gamma, Delta, and Omicron, EC_50_ = 0.13 – 0.26 µM) and HCoV‐OC43, indicating its broad‐spectrum antiviral activity. It is worthy that **C5N17B** retains antiviral activity against nirmatrelvir‐resistant strains with T21I/E166V and L50F/E166V mutations in M^pro^ (EC_50_ = 0.26 and 0.15 µM, respectively). Furthermore, **C5N17B** displays favorable pharmacokinetic properties. Crystallography studies reveal a unique, non‐covalent multi‐site binding mode. In conclusion, these findings substantiate the potential of **C5N17B** as an up‐and‐coming drug candidate targeting SARS‐CoV‐2 M^pro^ for clinical therapy.

## Graphical abstract

Click chemistry‐based miniaturized synthesis and direct screening of products at SARS‐CoV‐2 main protease (M^pro^), culminated in the discovery of a potent inhibitor, **C5N17B**, which exhibits broad‐spectrum antiviral activity and excellent anti‐drug resistance profiles. This study presents a paradigm for the rapid identification of enzyme inhibitors and provides a potential drug candidate for the treatment of SARS‐CoV‐2

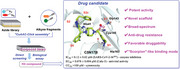

## 1 Introduction

The coronavirus disease 2019 (COVID‐19) pandemic, caused by the severe acute respiratory syndrome coronavirus 2 (SARS‐CoV‐2), was declared over in May 2023 but continues to pose a considerable public health concern, accounting for nearly 800 million confirmed cases and over 6.98 million deaths as of the end of April 2024.^[^ ^1^, ^2^ ^]^ The widespread global prevalence of SARS‐CoV‐2 and its robust human‐to‐human transmission was alarming both during and after the COVID‐19 pandemic. Due to its error‐prone RNA‐dependent RNA polymerase, the virus continues to undergo genetic mutations, giving rise to the emergence of novel variants with limited sensitivity to existing antivirals, and substantial immune evasion capabilities that can bypass acquired immunity from vaccination or prior infection, thereby instigating subsequent waves of infections.^[^ ^3^, ^4^ ^]^ A residual risk of SARS‐CoV‐2 outbreaks remains in various countries, and the potential co‐circulation of multiple respiratory diseases, including influenza, and pneumonia caused by unidentified pathogens, poses a formidable and terrifying challenge.^[^ ^5^ ^]^ In the aftermath of the COVID‐19 pandemic, it is imperative to develop highly effective and less toxic broad‐spectrum antiviral drugs that can combat drug‐resistant strains potentially emerging through accumulated use. These drugs should be considered an integral component of our pharmaceutical arsenal, pivotal in addressing recurrent outbreaks and mitigating the risk of potential future coronavirus‐induced diseases.

**Figure 1:**
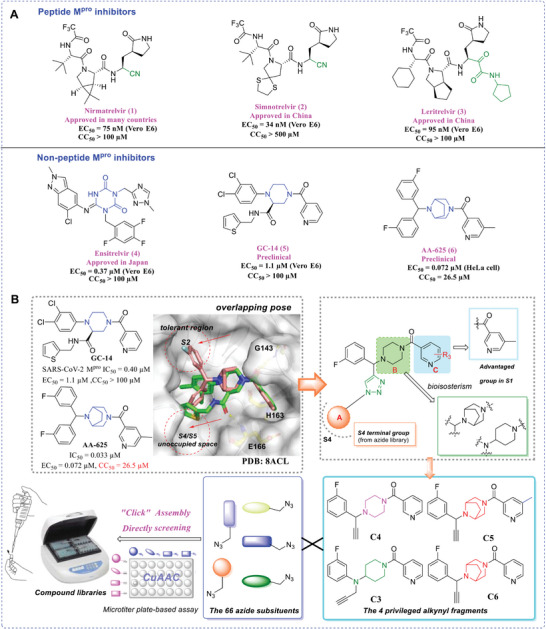
A) Structures of the marketed drugs, including **Nirmatrelvir**, **Simnotrelvir**, **Leritrelvir**, and **Ensitrelvir**, and the preclinical compounds **GC‐14** and **AA‐625**. EC_50_ values and CC_50_ values correspond to antiviral effects and cytotoxicity, respectively. B) Illustration of the general design concept of focused compound libraries.

The main protease (M^pro^, also known as 3CL^pro^) is a homodimeric cysteine protease,^[^ ^6^ ^]^ whose specific function is to recognize and cleave polyproteins (pp1a and pp1ab) into non‐structural proteins Nsp4 through Nsp16.^[^ ^7^ ^]^ Due to its crucial role in the production of mature viral proteins during the viral life cycle and the lack of analogous human proteases with comparable cleavage specificity,^[^ ^8^ ^]^ M^pro^ has emerged as an ideal target for drug design aimed at treating SARS‐CoV‐2 infections. Currently, M^pro^ inhibitors available in the market or undergoing clinical development encompass peptidomimetic covalent inhibitors such as nirmatrelvir (**1**, PF‐07321332),^[^ ^9^ ^]^ simnotrelvir (**2**, SIM0417),^[^ ^10^ ^]^ and leritrelvir (**3**, RAY1216),^[^ ^11^ ^]^ and non‐covalent inhibitors, e.g. ensitrelvir (**4**, S‐217622)^[^ ^12^ ^]^ and the preclinical candidate small molecule inhibitors **GC‐14** (**5**)^[^ ^13^ ^]^ and **AA‐625** (**6**)^[^ ^14^ ^]^ (**Figure** **1**A). However, peptidomimetic‐based inhibitors share similar structures, rendering them susceptible to comparably inducing drug‐resistant mutations.^[^ ^15^ ^]^ Besides, most of these antiviral agents exhibit poor metabolic stability and necessitate co‐administration with a cytochrome P450 enzyme inhibitor, e.g. ritonavir, to extend the antiviral drug's duration of exposure.^[^ ^16^ ^]^ Furthermore, due to the high mutation rates of its RNA genome, SARS‐CoV‐2 quickly develops drug resistance upon the widespread use of direct‐acting antiviral drugs.^[^ ^17^ ^]^ Resistance to nirmatrelvir has been observed in SARS‐CoV‐2, attributed to multiple mutations in M^pro^. For instance, the T21I/E166V and L50F/E166V double mutants of M^pro^ confer robust resistance to nirmatrelvir, while maintaining acceptable enzymatic activity.^[^ ^18^, ^19^, ^20^ ^]^ The potential occurrence of such mutations in future virus strains raises great concerns. Thus, there is an urgent need to develop novel technologies and strategies to discover small molecule drugs with unique scaffolds and improved antiviral activity against multiple coronaviral strains.

Non‐peptide inhibitors, represented by ensitrelvir,^[^ ^12^ ^]^ present advantages in terms of metabolic stability. Our previous research efforts have led to the discovery of **GC‐14**, a novel compound with a multi‐substituted piperazine scaffold (IC_50_ = 0.4 µM, EC_50_ = 1.1 µM),^[^ ^13^ ^]^ which demonstrates promising efficiency against SARS‐CoV‐2 and exceptional target specificity. However, its cellular activity is inferior to that of nirmatrelvir, and further enhancement of its pharmacokinetic (PK) properties is required. A crystallographic study of **GC‐14** bound to M^pro^ revealed that an appropriately multi‐substituted piperazine would be a feasible scaffold (PDB ID: 8ACL). **AA‐625**, a highly potent M^pro^ inhibitor, features a piperazine‐like diazabicyclooctane scaffold. However, this compound exhibits significant cytotoxicity (CC_50_ = 22.5 µM).^[^ ^14^ ^]^ Consequently, the present study aims to identify a non‐covalent M^pro^ inhibitor based on a central piperazine‐like scaffold with improved antiviral efficacy, lower toxicity, and suitable drug‐like properties.

Click chemistry serves as a versatile “chemical toolbox” that employs rapid modular synthesis to achieve broad functionalization of bioactive molecules. The prompt assembly and in situ screening of focused combinatorial libraries using copper(I)‐catalyzed alkyne‐azide cycloaddition (CuAAC), also termed click chemistry, represents a highly robust and efficient strategy for discovering bioactive compounds.^[^ ^21^, ^22^ ^]^ Therefore, we set out to apply miniaturized click chemistry‐based synthesis techniques to explore novel scaffolds as potent non‐peptide M^pro^ inhibitors. This innovative approach proved to be exceedingly effective.^[^ ^23^ ^]^

Docking studies predicted that **AA‐625** binds to M^pro^ in a similar pattern to that of **GC‐14,** but both S2 and S4 cavities have large unoccupied spaces to potentially accommodate additional groups (Figure 1B). Based on a multi‐site binding strategy, we replaced one of the 3‐fluorophenyl groups in the lead compound **AA‐625** with a 1,3‐disubstituted 1,2,3‐triazole, which can extend further into nearby sub‐pockets. Our optimization aimed to explore the chemical space in the M^pro^ S2 and S4 cavities, thereby establishing additional interactions and enhancing target affinity. Through CuAAC reaction between alkynes and azides, large‐scale combinatorial libraries were built without requiring laborious synthesis and purification. Active candidates identified by screening were then re‐synthesized at milligram scale, with subsequent determination of concentration‐dependent enzyme inhibitory activities and cell culture‐based antiviral activities. Representative compounds underwent further extensive evaluation for their anti‐drug resistance properties and broad‐spectrum antiviral efficacy, followed by the determination of co‐crystal structures, in vivo toxicity, and PK properties. Taken together, our study illuminates the benefit of miniaturized click chemistry in developing SARS‐CoV‐2 M^pro^ inhibitors. The identified hit compound **C5N17B** shows promise for future clinical development since it effectively inhibits various coronaviruses, including clinical variants and nirmatrelvir‐resistant strains of SARS‐CoV‐2.

## 2 Results

### 2.1 Chemical Synthesis of Target Compounds

**Scheme 1:**
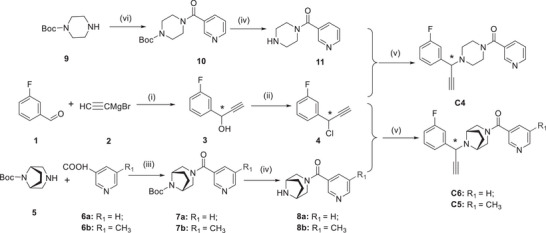
Synthetic route to the intermediates and alkyne fragments **C4, C5,** and **C6** ^a^.^a^Reagents and conditions: i) THF, −20 °C, N_2_; ii) dichlorosulfoxide, dichloromethane (DCM), ice bath; iii) 3‐(ethyliminomethylideneamino)‐*N*,*N*‐dimethylpropan‐1‐amine, hydrochloride (EDCI), 1‐Hydroxybenzotriazole (HOBt), *N*‐methylmorpholine (NMM), DCM, r.t.; iv) hydrogen chloride dioxane solution (4 m), DCM, r.t.; v) CH_3_COOK, methanol (MeOH), 70 °C; vi) *O*‐(7‐azabenzotriazol‐1‐yl)‐*N,N,N″,N″*‐tetramethyluronium hexafluorophosphate (HATU), *N,N*‐diisopropylethylamine (DIPEA), DCM, r.t.

**Scheme 2:**
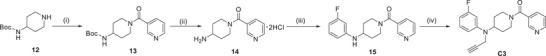
Synthetic Route to the Intermediates and Alkyne fragment **C3** ^a^; ^a^Reagents and conditions: i) HATU, DIPEA, niacin, DCM, r.t.; ii) hydrogen chloride dioxane solution (4 m), DCM, r.t.; iii) 3‐fluorophenylboronic acid, Cu(OAc)_2_, pyridine, O_2_, DCM,r.t.; iv) 3‐bromopropyne, t‐BuOK, ethanol, 60 °C.

The general procedures employed for the synthesis of the alkynes C4 to C6 are outlined in **Schemes** **1**, **2**. As depicted in Scheme 1, starting with raw materials 3‐fluorobenzaldehyde **1** and ethynylmagnesium chloride **2**, intermediate **3** was obtained through the Grignard reaction. Then, intermediate **4** was formed by the treatment of thionyl chloride. Intermediate **7a** or **7b** were synthesized from commercially available *tert*‐butyl (1*R*,5*S*)−3,8‐diazabicyclo[3.2.1]octane‐8‐carboxylate **5** and substituted nicotinic acid **6a** or **6b** through amide condensation, followed by the deprotection reaction to obtain intermediate **8a** or **8b**. The key fragments C6 and C5 were obtained from a substitution reaction between **4** and **8a** or **8b**.

The intermediate **10** was obtained through an amide condensation reaction using 2‐(7‐diazabenzotriazole)‐*N*,*N*,*N*″,*N*″‐tetramethyluronium hexafluorophosphate (HATU) as the coupling reagent in dichloromethane solvent from commercially available 1‐Boc‐piperazine **9** and nicotinic acid **6a**. Subsequently, intermediate **11** was obtained by removing the *t*‐butyloxyl carbonyl (Boc) protecting group. Finally, intermediate **11** was combined with intermediate **4** through a substitution reaction to form the key alkyne fragment C4.

Scheme 2 illustrates the synthesis of the alkyne C3, the intermediate **13** was obtained through an amide condensation reaction from commercially available 4‐Boc‐aminopyridine **12** and nicotinic acid **6a**. Then, followed by the deprotecting reaction to obtain intermediate **14**. Intermediate **15** was synthesized by employing the Cham‐Lam coupling reaction between **14** and 3‐fluorophenylboronic acid in the presence of copper acetate. Finally, the key alkyne fragment C3 was formed by a substitution reaction between **15** and 3‐bromopropyne.

### 2.2 Construction of Focused Libraries by Using Click Chemistry

**Figure 2:**
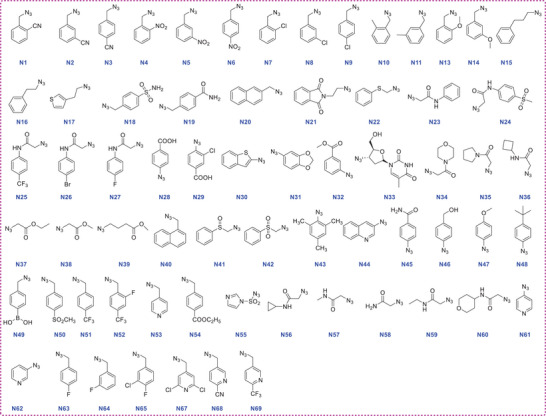
Structures of azides (N1 to N69) employed as starting materials.

Using the CuAAC reaction, we assembled focused libraries containing 268 triazole derivatives with three different scaffold types in microtiter plates on a microgram scale. For the central scaffold containing the alkyne substrate, we adopted a bicyclooctane ring (as in **AA‐625**), along with a piperazine ring or a 4‐aminopiperidine ring system. The azide counterparts were acquired from our in‐house azide library consisting of diverse drug‐like fragments. Each of the alkynes C3 to C6 was combined with azides N1 to N69 in separate reactions (**Figure** **2**). Subsequently, catalytic amounts of tris‐[(1‐benzyl‐1*H*‐1,2,3‐triazol‐4‐yl)methyl]amine (TBTA), CuSO_4_ **·**5H_2_O and sodium ascorbate solutions were added using multi‐channel pipettes. The specific reaction conditions and equivalents of reactants were systematically optimized (Table S1, Supporting Information). In all reactions, 1.0 equivalent of alkyne and 1.4 equivalents of azide were employed. Then, the reaction mixtures were shaken at 37 °C for 24 h on the constant temperature oscillation incubator. The progress of the reactions was monitored by TLC and/or LC‐MS to track the disappearance of the alkynes and the formation of the triazole products, using **C5N5**, **C5N6**, **C5N16**, **C5N17**, **C5N21**, **C5N39** and **C5N58**, as examples (Figures S1–S7, Supporting Information). Finally, each well received an addition of 100 µL DMSO and was further shaken for 1 h to form stock solutions of crude products. These solutions were subsequently transferred to labeled tubes and stored at −20 °C before screening.

### 2.3 Direct Screening of Focused Libraries against SARS‐CoV‐2 Main Protease

The compounds were screened for inhibition of SARS‐CoV‐2 M^pro^ using a commercially available assay kit based on the fluorescence resonance energy transfer (FRET) method.^[^ ^24^ ^]^ The generated library of triazole derivatives was diluted without further purification, and preliminarily screening was performed to assess the inhibitory activity of the compounds at a concentration of 10 µM or lower. The lead compounds **GC‐14** and **AA‐625** were selected as positive controls. A solution containing TBTA, CuSO_4_ **·**5H_2_O, and sodium ascorbate, corresponding to the blank reaction medium, was used as a negative control. A total of 16 hits showing over 80% inhibition of M^pro^ enzymatic activity emerged from the first round of screening of the four crude compound libraries: 2 hits from the C4 series, 12 hits from the C5 series, and 2 hits from the C6 series (Figures S8–S11, Supporting Information). The overall enzyme inhibition of the **C3NX** series was marginal, failing to yield hits. Encouragingly, for the **C4** alkyne building block, the inhibition percentages of **C4N17** (86%) and **C4N21** (82%) showed higher or comparable enzyme inhibition to that of **AA‐625** (57%) and **GC‐14** (98%). In the case of the C5 alkyne building block, it is noteworthy that most of the compounds exhibited higher activity than **GC‐14** and **AA‐625**, including **C5N5** (93%), **C5N6** (88%), **C5N16** (99%), **C5N17** (101%), **C5N21** (100%), **C5N39** (96%), **C5N41** (100%), **C5N42** (101%), **C5N50** (90%), **C5N57** (96%), **C5N58** (99%) and **C5N64** (93%). For the alkyne fragment **C6**, the inhibitory potencies of **C6N17** (90%) and **C6N21** (89%) were found to be either higher than or comparable to those of **AA‐625** and **GC‐14**.

### 2.4 Biological Evaluation of the Purified Hit Compounds Selected from Screening

Next, 16 selected hit compounds were individually synthesized and purified on a milligram scale as racemic mixtures for accurate IC_50_ value determination. The activities of nirmatrelvir, ensitrelvir, and the two lead compounds, **GC‐14** and **AA‐625** were evaluated alongside in the same assay. As indicated in **Table** **1**, all purified compounds from the C5 series demonstrated inhibitory effects with IC_50_ values ranging from 0.150 to 2.50 µM. Consequently, compounds **C5N21** (IC_50_ = 0.150 ± 0.004 µM) and **C5N58** (IC_50_ = 0.151 ± 0.006 µM) were identified as the most promising inhibitors against M^pro^. They demonstrated enhanced activity over the lead compounds **GC‐14** (IC_50_ = 0.750 ± 0.030 µM) and **AA‐625** (IC_50_ = 0.904 ± 0.015 µM) by 5‐ and 6‐fold, respectively, and displayed comparable potency to the positive controls nirmatrelvir (IC_50_ = 0.091 ± 0.010 µM) and ensitrelvir (IC_50_ = 0.116 ± 0.020 µM). **C5N17** (IC_50_ = 0.177 ± 0.016 µM) and **C5N42** (IC_50_ = 0.196 ± 0.009 µM) likewise elicited considerable inhibitory effects. These results warranted further investigation of cellular antiviral activity.

**Table 1:** Chemical structures and biological activities of representative compounds of series C4NX to C6NX.

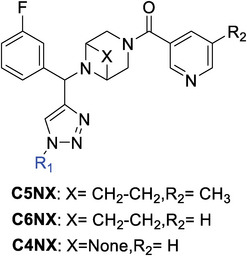					
Compounds	R_1_	IC_50_ ± SD [µM]^a)^	Compounds	*R*_1_	IC_50_ ± SD [µM]^a)^
GC‐14 (lead)	–	0.750 ± 0.030	Nirmatrelvir^b)^	–	0.0910 ± 0.010
AA‐625 (lead)	–	0.904 ± 0.015	Ensitrelvir ^b)^	–	0.116 ± 0.020
**C5N5**	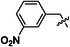	1.23 ± 0.001	**C5N6**	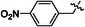	2.50 ± 0.013
**C5N16**	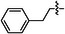	0.283 ± 0.018	**C5N17**	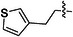	0.177 ± 0.016
**C5N21**	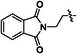	0.150 ± 0.004	**C5N39**	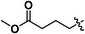	0.253 ± 0.010
**C5N41**	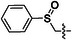	0.243 ± 0.011	**C5N42**	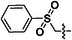	0.196 ± 0.009
**C5N50**	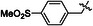	2.40 ± 0.10	**C5N57**	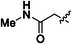	0.580 ± 0.005
**C5N58**	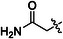	0.151 ± 0.006	**C5N64**	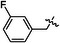	1.23 ± 0.10
**C4N17**	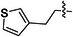	1.87 ± 0.013	**C4N21**	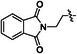	1.30 ± 0.017
**C6N17**	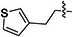	0.300 ± 0.012	**C6N21**	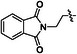	0.200 ± 0.016

^a)^ Enzyme inhibitory activity (SARS‐CoV‐2 M^pro^) determined by a standard fluorescence resonance energy transfer (FRET) assay. IC_50_ values are presented as means ± SD of at least three independent experiments performed in duplicate.

^b)^ Nirmatrelvir and ensitrelvir were used as positive controls (*n =* 3 biological replicates).

### 2.5 Antiviral Efficacy of the Hit Compounds against SARS‐CoV‐2 in Cells

The SARS‐CoV‐2 antiviral activity of selected 14 hit compounds was evaluated in immunofluorescence‐based antiviral assays, both in Vero 81 cells and Calu‐3 cells, to study potential cell type‐dependent antiviral effects.^[^ ^25^ ^]^ Since Vero cells have been reported to express high levels of the efflux transporter P‐glycoprotein,^[^ ^9^, ^26^ ^]^ we investigated the effect of the P‐glycoprotein inhibitor CP‐100356 on antiviral activity in the different cell lines. In Vero cells, the antiviral activities of the active compounds ranged between 1.57 and 36.1 µM in the absence of CP‐100356, and from 0.363 to 6.57 µM in its presence (**Table** **2**). A consistent reduction in EC_50_ values by CP‐100356 was also observed in Calu‐3 cells, ranging from 0.166 to 74.4 µM without CP‐100356 and from 0.028 µM to 4.02 µM with it. These results indicated that the efflux pump‐dependent antiviral activity of the hit compounds varies with the chemical structure and the cell line. For instance, in the absence of CP‐100356, the antiviral activities of **C5N21** (EC_50_ = 16.2 µM, Vero / EC_50_ = 0.591 µM, Calu‐3) and **C5N58** (EC_50_ >100 µM / 4.94 µM, respectively) were relatively weak, whereas both ensitrelvir (EC_50_ = 0.165 µM, Vero / EC_50_ = 0.112 µM, Calu‐3) and nirmatrelvir (EC_50_ = 2.08 µM / 1.00 µM, respectively) exhibited stronger efficacy (Table 2). In the presence of CP‐100356, the EC_50_ values of **C5N21** (1.38 µM, Vero / 0.259 µM, Calu‐3) and **C5N58** (1.39 µM / 0.337 µM, respectively) were remarkably improved, but remained higher than those of ensitrelvir and nirmatrelvir, whose EC_50_ values were below the detection limit of 0.046 µM.

**Table 2:** Anti‐SARS‐CoV‐2 activity and cytotoxicity of C5NX and C4NX compounds determined in Vero cells and Calu‐3 cells.

Compounds	Vero cells			Calu‐3 cells		
	EC_50_ ± SD (µM)^a)^		CC_50_ (µM)^b)^	EC_50_ ± SD (µM)^a)^		CC_50_ (µM)
	without P‐gp inhibitor^c)^	with P‐gp inhibitor		without P‐gp inhibitor	with P‐gp inhibitor	
**C5N5**	1.79 ± 0.021	1.85 ± 0.526	>100	2.86 ± 0.395	2.56 ± 0.086	>100
**C5N6**	1.83 ± 0.030	1.74 ± 0.238	>100	0.767 ± 0.156	1.05 ± 0.014	>100
**C5N16**	2.00 ± 0.021	1.89 ± 0.524	>100	0.417 ± 0.018	0.080 ± 0.002	>100
**C5N17**	1.57 ± 0.131	0.363 ± 0.029	>100	0.166 ± 0.011	0.028 ± 0.002	>100
**C5N21**	16.2 ± 0.410	1.38 ± 0.119	>100	0.591 ± 0.011	0.259 ± 0.043	>100
**C5N39**	1.87 ± 0.270	1.60 ± 0.249	>100	0.899 ± 0.312	0.253 ± 0.026	>100
**C5N41**	3.67 ± 0.513	2.27 ± 0.089	>100	1.78 ± 0.001	0.097 ± 0.005	>100
**C5N42**	3.68 ± 0.952	3.05 ± 0.206	>100	1.77 ± 0.055	0.133 ± 0.019	>100
**C5N50**	36.1 ± 1.38	6.57 ± 0.430	>100	17.0 ± 0.778	2.90 ± 0.263	>100
**C5N57**	>100	>10	>100	4.04 ± 0.986	2.33 ± 0.066	>100
**C5N58**	>100	1.39 ± 0.171	>100	4.94 ± 0.687	0.338 ± 0.056	>100
**C5N64**	4.20 ± 0.738	2.86 ± 0.131	>100	2.94 ± 0.124	0.479 ± 0.026	>100
**C4N17**	2.74 ± 0.110	2.46 ± 0.314	>100	1.85 ± 0.139	0.880 ± 0.109	>100
**C4N21**	>69.8	>10	69.8 ± 1.05	74.4 ± 36.3	4.02 ± 0.566	>100
**AA‐625**	2.05 ± 0.056	2.02 ± 0.076	43.9 ± 2.94	0.148 ± 0.007	<0.01	>100
**GC‐14**	n.t.^d)^	3.42 ± 0.443	n.t.	1.42 ± 0.057	0.755 ± 0.046	>100
**Ensitrelvir**	0.165 ± 0.015	<0.046	>100	0.112 ± 0.011	<0.046	>100
**Nirmatrelvir**	2.08 ± 0.214	<0.046	>100	1.00 ± 0.031	<0.046	>100

^a)^ 50% effective concentration. Antiviral activity was determined in Vero cells (from African green monkey kidney) and Calu‐3 cells (from human lung adenocarcinoma) using an immunofluorescence‐based antiviral assay. EC_50_ values are means ± SD of at least three independent experiments performed in duplicate.

^b)^ 50% cytotoxicity concentration. Cytotoxicity values (CC_50_) were measured in Vero and Calu‐3 cells using an MTT assay.

^c)^ P‐glycoprotein inhibitor, CP‐100356.

^d)^ Not tested.

From a simplified comparative analysis of antiviral efficacy in Vero cells, excluding the participation of CP‐100356, we observed that the antiviral efficacy of **C5N5**, **C5N6**, **C5N16**, **C5N17** and **C5N39** (EC_50_ = 1.57 – 2.00 µM) was comparable to that of **AA‐625** (EC_50_ = 2.05 µM) and nirmatrelvir (EC_50_ = 2.08 µM) (Table 2). Other compounds, including **C5N41**, **C5N42**, **C5N64,** and **C4N17**, were less potent than the two control compounds, resulting in EC_50_ values between 2.74 and 4.20 µM. The remaining compounds were marginally active (EC_50_ = 16.2 µM for **C5N21**, and EC_50_ = 36.1 µM for **C5N50**) or lost antiviral activity (EC_50_ > 100 µM for **C5N57** and **C5N58**, and EC_50_ > 69.8 µM for **C4N21**). Fortunately, all of the compounds, except for **C4N21** (CC_50_ = 69.8 µM), were non‐toxic to Vero cells, at least at concentrations up to 100 µM.

In Calu‐3 cells, derived from human lung epithelial cells and considered more representative of a SARS‐CoV‐2‐infected pathophysiological condition, the overall EC_50_ values were lower as compared to those determined in Vero cells. Notably, **C5N17** was the most potent compound (EC_50_ = 0.166 µM), positioning it between **AA‐625** (EC_50_ = 0.148 µM) or ensitrelvir (EC_50_ = 0.112 µM) and **GC‐14** (EC_50_ = 1.42 µM) or nirmatrelvir (EC_50_ = 1.00 µM). Other active compounds, including **C5N6**, **C5N16**, **C5N21**, and **C5N39**, exhibited considerable antiviral activity with EC_50_ values below 1 µM, whereas **C5N5**, **C5N41**, **C5N42**, **C5N57**, **C5N58**, **C5N64** and **C4N17** had EC_50_ values ranging 1 to 10 µM. However, anti‐SARS‐CoV‐2 efficacy was greatly reduced in the remaining compounds, including **C5N50** (EC_50_ = 17.0 µM), and **C4N21** (EC_50_ = 74.4 µM). No compound was toxic to Calu‐3 cells (CC_50_ > 100 µM). From the antiviral assay data against SARS‐CoV‐2, collected in the pathophysiologically more relevant Calu‐3 cells, it was concluded that **C5N16** and **C5N17** were the most potent antiviral compounds among the 14 selected test compounds. Particularly **C5N17** was comparable to the lead compound **AA‐625** and more potent than the other lead compound, **GC‐14**.

Again, the results from the enzymatic activity assays and the cell‐based antiviral assays showed an inconsistent pattern. For instance, **C5N21** and **C5N58**, endowed with the highest enzyme inhibitory activity, exhibited drastically reduced antiviral activity in Vero 81 and Calu‐3 cells, when compared to nirmatrelvir or ensitrelvir (Tables 1 and 2). **C5N58** completely lost antiviral efficacy in Vero 81 cells. This can be likely explained by their poor membrane permeability, sensitivity to P‐gp, or rapid degradation in cells. Gratifyingly, **C5N17** was identified as the most potent antiviral agent in the cellular assays.

Subsequently, we explored the structure‐activity relationships (SARs) of the new compounds. Combining the primary screening results of combinatorial libraries and the determined IC_50_ and EC_50_ values, a unique pattern of SARs was observed. For S2 or S4 groups, there is a strong preference for an aromatic system linked by a 2‐atom chain. **C5N16** and **C5N17** were the most potent compounds with this feature. Various substituted benzyl groups also showed comparably high activity, presumably because the two cavities have some degree of flexibility. However, aromatic rings directly attached to the triazole ring are not tolerated, causing significantly decreased activities. Another interesting finding is that an acetamide, a polar hydrophilic group, also led to potent M^pro^ inhibitory activity as shown for **C5N58**. However, additional groups attached to the terminal amino group of the acetamide resulted in reduced potency. In cellular antiviral assays, **C5N21** and **C5N58** were less potent than **C5N16** and **C5N17**, probably due to their poor cell permeability. Moreover, derivatives harboring a diazabicyclooctane as the central scaffold, exhibited higher activity as compared to the corresponding piperazine derivatives, while a 4‐aminopiperidine as the central scaffold failed to improve compound activity. The introduction of an extra bridge restricts the conformation of the ligands and fixes the bioactive conformation of the compounds, thereby facilitating its binding to the M^pro^ active center. Although the 5‐methyl group of a nicotinyl moiety does not appear to form any additional interactions with protein residues in the S1 cavity, it plays a pivotal role in the compounds’ activity, acting as a “magic methyl group”.^[^ ^27^ ^]^ How the S1 substituent affects compound activity remains to be clarified.

**Figure 3:**
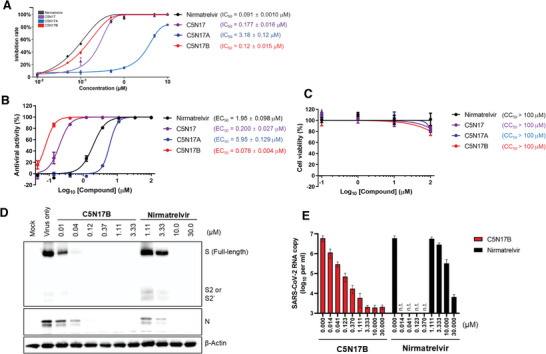
Effects of the racemate **C5N17** and its two enantiomers (**C5N17A**, **C5N17B**) on M^pro^ inhibition and antiviral efficacy determined in a cellular assay. A) M^pro^ inhibitory potency of **C5N17**, **C5N17A**, **C5N17B,** and Nirmatrelvir. B) Anti‐SARS‐CoV‐2 activity of **C5N17**, **C5N17A**, **C5N17B,** and Nirmatrelvir in Calu‐3 cells. C) Toxicity of **C5N17**, **C5N17A**, **C5N17B,** and Nirmatrelvir on Calu‐3 cells. D) Western blot analysis for detecting viral S and N proteins in Calu‐3 cells in the presence of **C5N17B** or Nirmatrelvir on day 2 post‐infection. E) Quantitative RT‐PCR for measuring SARS‐CoV‐2 viral RNA amounts accumulated in Calu‐3 cell culture supernatants in the presence of increasing concentrations of **C5N17B** or Nirmatrelvir on day 2 post‐infection. n.t., not tested. In all experiments, Nirmatrelvir was used as a positive control. In panels (A–C) and (E), all values are represented as means ± SD from three independent experiments.

Given the presence of a chiral carbon atom in the potent compounds, we conducted chiral HPLC separation of **C5N17** to investigate the dependency of the M^pro^ inhibitory effect and of the cellular antiviral efficacy on the stereochemistry of the compound. The absolute stereochemistry of **C5N17** was determined by structural biology studies. A significant disparity was observed in enzyme as well as in antiviral activities between the two diastereomers of **C5N17**, named **C5N17A** and **C5N17B**. The *(R)‐*enantiomer **C5N17B** (IC_50_ = 0.120 µM, EC_50_ = 0.0780 µM) exhibits 27‐fold stronger enzyme inhibitory activity and 76‐fold more potent antiviral activity determined in cells than the *(S)‐*configurated **C5N17A** (IC_50_ = 3.18 µM, EC_50_ = 5.95 µM; **Figure** **3**A,B). Additionally, to further investigate the target affinity, *K*_i_ value for **C5N17B** was determined, along with those of nirmatrelvir and ensitrelvir. The *K*_i_ values were acquired based on a reported *K*_M_ value and Cheng‐Prusoff equation^[^ ^28^, ^29^ ^]^ The results revealed that **C5N17B** displayed a *K*_i_ value of 26.6 nm, indicating strong competitive inhibition with the peptide substrate. This is similar to nirmatrelvir (*K*_i_ = 16.3 nm) and ensitrelvir (*K*_i_ = 16.6 nm) in terms of target affinity (Tables S2 and S3, Figure S12, Supporting Information). Notably, the antiviral activity of **C5N17B** surpassed that of the marketed drug nirmatrelvir (IC_50_ = 0.0910 µM, EC_50_ = 1.95 µM) when measured in the same system. These compounds do not affect Calu‐3 cell viability, maintaining over 80% cell survival at a high concentration of 100 µM (Figure 3C).

Next, we used **C5N17B** as an example to confirm whether the observed reduction in fluorescence intensity in the cell culture‐based antiviral assay actually corresponds to the inhibition of SARS‐CoV‐2 amplification in cells. The quantitative examination of spike (S) / nucleocapsid (N) protein within host cells and the production of progeny virus in culture supernatants was conducted by Western blot analysis and qRT‐PCR, respectively. The Western blot clearly illustrated a dose‐dependent reduction in SARS‐CoV‐2 S and N proteins in virus‐infected cells upon treatment with **C5N17B** (Figure 3D). Consistent with this observation, the reduction in viral RNA exceeded 3‐log orders, reaching a plateau at a concentration of 3.33 µM **C5N17B** (Figure 3E). There was a 20‐fold decrease in viral RNA copy number at 0.041 µM **C5N17B** compared to 10.0 µM nirmatrelvir, indicating ≈250‐fold greater potency of the former compound. Collectively, these results suggest that **C5N17B** has the capability to block the accumulation of viral protein and viral RNA by inhibiting M^pro^ enzymatic activity in Calu‐3 cells with significantly greater potency than nirmatrelvir.

### 2.6 Crystallographic Study of C5N17B in Complex with M^pro^

**Figure 4:**
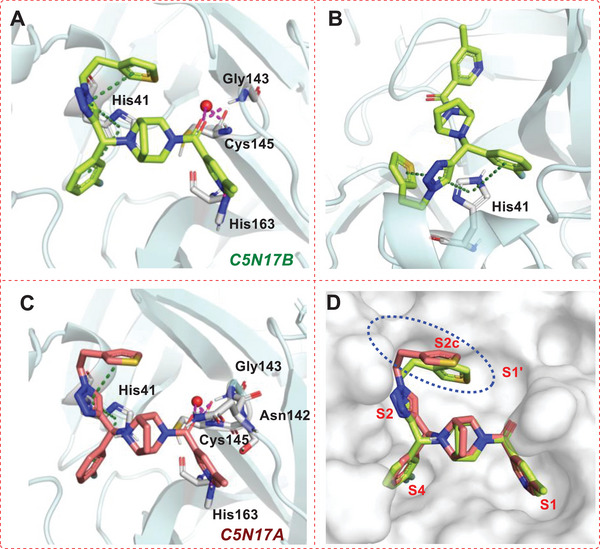
X‐ray co‐crystal structures of the (*R*)‐configurated **C5N17B** (PDB ID: 9G0I) and its (*S*)‐configurated enantiomer **C5N17A** (PDB ID: 9G0H) in complex with M^pro^. A) View of **C5N17B** (green) in the binding pocket. Hydrogen bonds are shown as magenta‐colored dashed lines; π–π stacking is indicated as green dashed lines. B) View of the interactions of **C5N17B** (green) with His41. C) View of **C5N17A** (red) in the binding pocket. D) Binding pose comparison of **C5N17B** (green) and **C5N17A** (red).

X‐ray co‐crystal structures of M^pro^ in complex with **C5N17B** and its less potent enantiomer **C5N17A** were obtained (Table S4, Supporting Information). The electron density maps with anisotropic resolution limits of 2.25 to 1.64 Å (**C5N17A**) and 2.07 to 1.63 Å (**C5N17B**) made it possible to clearly identify the configurations of the chiral carbon centers and the binding modes (Figure S13, Supporting Information). The more potent **C5N17B** represents the (*R*)‐enantiomer of **C5N17**. The bridged diazabicyclooctane ring system served as a central scaffold to support the “multi‐site” binding mode of **C5N17B** (**Figure** **4**A). Specifically, in the S1 pocket, the *N*‐atom of the pyridine forms a strong H‐bond with the imidazole‐*N*H group of His163, which plays a crucial and advantageous role in enhancing antiviral activity. The carbonyl group of the 5‐methylnicotinoyl group interacts via hydrogen bonds with the backbone *N*H‐groups of Gly143 (directly) and Cys145 (via a water bridge). The 3‐fluorophenyl group of **C5N17B** is positioned in the hydrophobic S2 pocket close to the more hydrophobic S4 cavity (Figure S13C, Supporting Information). The triazole ring also binds to the S2 pocket. Both ring systems form stacking interactions with the imidazole side chain of His41 (Figure 4B). The (thiophene‐3‐yl)ethyl group binds to the S1' pocket. The two‐carbon linker allows the thiophene ring to fit into a shallow groove surrounded by Thr25, Leu27, His41, and Val42. Interestingly, no hydrogen bonds involving Glu166 are observed, which is distinct from other potent M^pro^ inhibitors.

Although it shows a weaker affinity for M^pro^, the (*S*)‐enantiomer **C5N17A** (Figure 4C) demonstrates a similar overall binding mode as **C5N17B** (Figure 4D, Figure S13D, Supporting Information). However, since the *N*8 atom of the central scaffold is connected to the chiral carbon atom, its exocyclic *N*8‐C bond is forced to flip into the axial position via inversion at the nitrogen. The thiophene ring in **C5N17A** is shifted 3.4 Å away from the S2 surface as a result of the ≈30° rotation of the triazole ring, which is induced by the different configuration of the chiral carbon. This shift leads to higher solvent exposure and instability of the thiophene ring, as indicated by the lack of electron density of this group (Figure S13A, Supporting Information), which contributes to the lower binding affinity of **C5N17A**. Because the substituents on the *N*‐atom shift into the axial position in the (*S*)‐enantiomer **C5N17A**, this compound loses binding energy in comparison to its (*R*)‐enantiomer **C5N17B**, where the substituents remain in an energetically favorable equatorial position. The instability of **C5N17A** is also confirmed by molecular dynamics simulations, which provided additional support for our conjecture. (Figure S14, Supporting Information).

**Figure 5:**
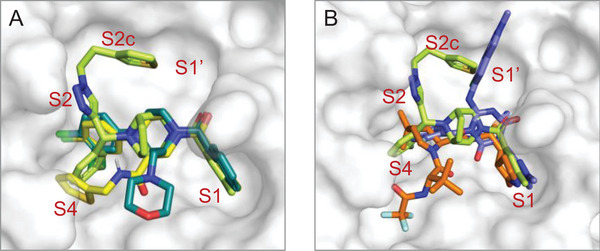
Comparison of the M^pro^ binding modes of **C5N17B** and selected other inhibitors with piperazine scaffolds. A) Binding pose comparison of **C5N17B** (green), **GC‐14** ^[^ ^13^ ^]^ (yellow, PDB ID: 8ACL), and **JZD‐07** ^[^ ^30^ ^]^ (cyan, PDB ID: 8GTV). B) Superposition of the binding modes of **C5N17B** (green), **nirmatrelvir** (orange, PDB ID: 7VH8), and **ensitrelvir** (blue, PDB ID: 7VU6).

Compared to **GC‐14** and another multi‐substituted piperazine derivative, **JZD‐07** (Figure S15, Supporting Information),^[^ ^13^, ^30^ ^]^ **C5N17B** also fully occupies both the S1 and S2 sites (**Figure** **5**A), but varies in the binding pattern of the “side‐wing” thiophene group. Furthermore, in contrast to the binding mode of nirmatrelvir and ensitrelvir, **C5N17B** involves less hydrogen bonding with key residues but mainly forms hydrophobic interactions with the S2, S4, and S2c cavities, which was described by Han et al. (Figure 4D and 5B).^[^ ^31^ ^]^

### 2.7 Antiviral Activity of C5N17B against Nirmatrelvir‐resistant Mutants or SARS‐CoV‐2 Variants and Low Pathogenic Human Coronaviruses

**Figure 6:**
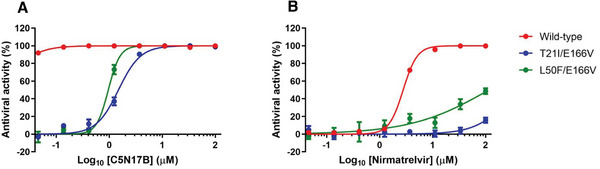
A) The comparison of **C5N17B** and nirmatrelvir B) against SARS‐CoV‐2 nirmatrelvir‐resistant mutants.

It has been reported that SARS‐CoV‐2 develops resistance to nirmatrelvir through mutations in M^pro^, such as T21I/E166V and L50F/E166V while maintaining substrate cleavage activity comparable to the wild‐type protein.^[^ ^19^, ^20^ ^]^ Using a reverse genetics technology,^[^ ^32^ ^]^ we generated recombinant wild‐type SARS‐CoV‐2, designated rgSARS‐CoV‐2, as well as its nirmatrelvir‐resistant mutants, designated rgSARS‐CoV‐2‐M^pro^/T21I+E166V and rgSARS‐CoV‐2‐M^pro^/L50F+E166V, respectively, in which GFP is expressed as a reporter. To investigate whether **C5N17** and its enantiomer **C5N17B** can distinguish themselves from nirmatrelvir in their ability to counteract nirmatrelvir‐resistant SARS‐CoV‐2 mutants, the antiviral efficacy was assessed against wild‐type rgSARS‐CoV‐2 as well as the mutant viruses grown in Calu‐3 cells. Nirmatrelvir and **AA‐625** were included as controls (**Figure** **6**, **Table** **3**).

**Table 3:** Antiviral activity of **C5N17B** against nirmatrelvir‐resistant SARS‐CoV‐2 mutant viruses.

Compounds	EC_50_ ± SD (µM)^a)^			CC_50_ [µM]^b)^
	Wild‐type rgSARS‐CoV‐2	rgSARS‐CoV‐2‐M^pro^/T21I +E166V	rgSARS‐CoV‐2‐M^pro^/L50F+E166V	
**C5N17B**	<0.046	0.260 ± 0.029	0.150 ± 0.019	>100
**C5N17**	0.441 ± 0.032	1.47 ± 0.123	0.966 ± 0.040	>100
**AA‐625**	0.779 ± 0.173	3.41 ± 0.821	5.71 ± 0.664	42.9 ± 0.414
**Nirmatrelvir**	2.77 ± 0.033	>100	>100	>100

^a)^ 50% effective concentration against viruses rescued by reverse genetics system in Calu‐3 cells.

^b)^ 50% cytotoxicity concentration to Calu‐3 cells. Values are represented as means ± SD of data derived from *n =* 3 independent experiments in duplicate.

The replication of recombinant rgSARS‐CoV‐2s was reflected by the GFP expression level and quantified in the presence of increasing concentrations of each compound. As anticipated, nirmatrelvir exhibited antiviral activity against the wild‐type virus (EC_50_ = 2.77 µM) but was not able to inhibit the replication of two mutant viruses at concentrations up to 100 µM (Table 3). In contrast, **AA‐625** demonstrated efficacy not only against the wild‐type virus (EC_50_ = 0.779 µM) but also against the mutants (EC_50_ = 3.41 µM for rgSARS‐CoV‐2‐M^pro^/T21I+E166V, and EC_50_ = 5.71 µM for rgSARS‐CoV‐2‐M^pro^/L50F+E166V), albeit being somewhat cytotoxic to Calu‐3 cells (CC_50_ = 42.9 µM). **C5N17**, with comparable antiviral activity to **AA‐625**, displayed antiviral activity against these three viruses with EC_50_ values ranging from 0.441 to 1.47 µM. Notably, **C5N17B** exhibited highly potent activity against the wild‐type recombinant virus (EC_50_ < 0.046 µM) and the mutant viruses (EC_50_s, 0.260, and 0.150 µM, respectively) (Table 3). The antiviral assays with the reverse genetically modified viruses strongly suggest that **C5N17B** can efficiently inhibit both wild‐type and nirmatrelvir‐resistant SARS‐CoV‐2, making it a promising candidate for pandemic preparedness in the event of circulation of nirmatrelvir‐resistant strains.

**Figure 7:**
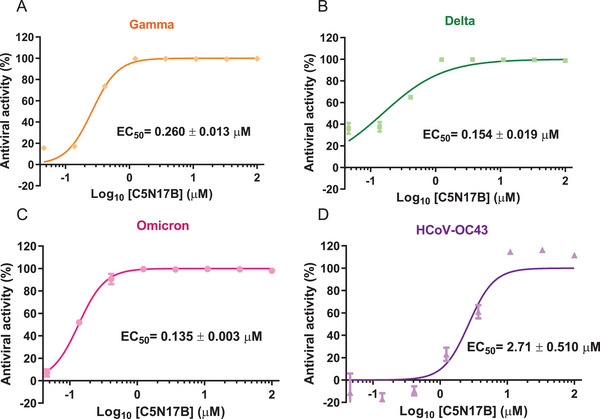
A–C) Antiviral activity of **C5N17B** against SARS‐CoV‐2 variants and D) HCoV‐OC43.

High mutation rates of the SARS‐CoV‐2 genome highlight the importance of discovering broad‐spectrum anti‐coronaviral agents. We therefore assessed the antiviral efficacy of **C5N17B** against various clinically isolated variants of SARS‐CoV‐2, as well as other *beta‐coronaviruses* (MERS‐CoV and HCoV‐OC43) and *alpha‐coronaviruses* (HCoV‐229E and HCoV‐NL63) (**Figure** **7**, **Table** **4**).^[^ ^33^ ^]^ **C5N17B** exhibited excellent antiviral activity against the original wide‐type strain and its circulating gamma, delta, and omicron variants, with EC_50_ values ranging from 0.119 to 0.260 µM. Its antiviral efficacy was ≈20‐fold higher than that of nirmatrelvir on average. However, **C5N17B** failed to inhibit infection of MERS‐CoV or HCoV‐229E, in contrast to nirmatrelvir, which inhibited those viruses with EC_50_ values of 0.0580 and 0.378 µM, respectively. Interestingly, HCoV‐OC43 was sensitive to both compounds, more significantly to nirmatrelvir (EC_50_ < 0.046 µM) than to **C5N17B** (EC_50_ = 2.71 µM), while HCoV‐NL63 was not sensitive to either compound (EC_50_ > 100 µM). Taken together, the broad‐spectrum antiviral evaluation suggests that our compounds have pan‐inhibition activity toward different SARS‐CoV‐2 strains while having reduced inhibitory efficacy against other CoVs. Our next challenge will be to enhance their antiviral effectiveness against a larger variety of human CoVs by synthesizing derivatives of **C5N17B**.

**Table 4:** Broad‐spectrum antiviral activities of **C5N17B**.

Genus	Pathogens	Isolates	Cell lines	C5N17B		Nirmatrelvir	
				EC_50_ ± SD [µM]^a)^ [S.I.^c)^]	CC_50_ (µM)^b)^	EC_50_ ± SD [µM] [S.I.]	CC_50_ [µM]
*Beta Coronavirus*	SARS‐CoV‐2	Wild‐type	Calu‐3	0.119 ± 0.011 (>837)	>100	2.78 ± 0.033 (>36.1)	>100
		Gamma		0.260 ± 0.013 (>385)		3.43 ± 0.519 (>29.1)	
		Delta		0.154 ± 0.019 (>649)		3.70 ± 0.598 (>27.0)	
		Omicron		0.135 ± 0.003 (>741)		3.39 ± 0.245 (>29.4)	
	MERS‐CoV	–	Huh7	>100 (n.d.^d)^)	>100	0.058 ± 0.010 (>29.4)	>100
	HCoV‐OC43	–	MRC‐5	2.71 ± 0.510 (>36.9)	>100	<0.046 (>2174)	>100
*Alpha* *Coronavirus*	HCoV‐229E	–	MRC‐5	>100 (n.d.^d)^)	>100	0.378 ± 0.020 (>29.4)	>100
	HCoV‐NL63	–	LLC‐MK2	>100 (n.d.^d)^)	>100	>100 (n.d.^d)^)	>100

^a)^ 50% effective concentration;

^b)^ 50% cytotoxicity concentration to Calu‐3 cells;

^c)^ Selectivity index, the ratio of CC_50_ to EC_50_;

^d)^ Not determined.

**Figure 8:**
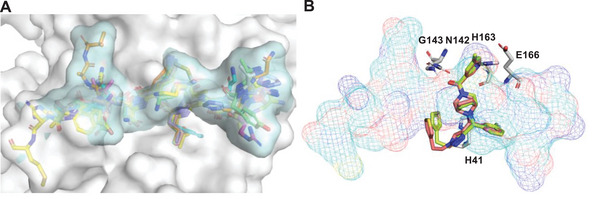
Comparison of the location of **C5N17B** and **C5N17A** in the substrate envelope of SARS‐CoV‐2 M^pro^. A) The 3D shape of the substrate envelope of SARS‐CoV‐2 M^pro^. B) The fitting of **C5N17B** (green) and **C5N17A** (brick red) within the substrate envelope.

The substrate envelope functions as the fundamental basis for molecular recognition by viral enzymes and comprehensively elucidates the selection of resistance mutations in the active site. This site of SARS‐CoV‐2 M^pro^ has also been defined (**Figure** **8**A).^[^ ^34^ ^]^ Interestingly, **C5N17B** is positioned favorably within the substrate envelope. However, the thiophene ring of **C5N17A** extends out of the envelope compared with **C5N17B** (Figure 8B). It was reported that the residues Met49, Asn142, Met165, Glu166, and Gln189 are prone to resistance mutations.^[^ ^34^, ^35^ ^]^ Especially, Glu166 is located at the S1 pocket and is a critical residue for drug binding. Notably, **C5N17B** does not seem to interact with these amino acid residues, thereby providing an explanation of why **C5N17B** is not affected by the nirmatrelvir‐resistant M^pro^ mutation E166V (Table 3).

The non‐peptide M^pro^ inhibitor ensitrelvir, exclusively forms hydrogen bonds with the main chain*N*H of Glu166, while lacking any interactions with Glu166 side chain, as revealed by co‐crystal structures with M^pro^.^[^ ^12^ ^]^ Notably, ensitrelvir has been reported to show moderate or low resistance against the E166V mutants. This specific mutation induces a conformational change in the S1 subsite, resulting in reduced binding affinity between ensitrelvir and M^pro^ and subsequently diminishing its antiviral efficacy. Among the identified mutations, E166V, T21I/E166V, and L50F/E166V exhibit the strongest resistance. Single mutation E166V significantly diminishes the binding ability of inhibitors, including nirmatrelvir and ensitrelvir, resulting in 300‐fold and 78‐fold increases in resistance respectively.^[^ ^32^, ^36^ ^]^ Therefore, minimizing interactions with amino acid residues prone to developing drug‐resistant mutations is a plausible strategy for combating the emergence of drug‐resistance variants.

### 2.8 In Vivo Pharmacokinetic Study of C5N17B

The pharmacokinetics profile of the newly developed antiviral compound **C5N17B** was subsequently evaluated in male ICR mice that were free from specific pathogens, aiming to assess its potential for therapeutic application in vivo. The PK parameters obtained from this study are summarized in **Table** **5**. Following intravenous administration at a dose of 2 mg kg^−1^ in mice (*n =* 3), the clearance rate (*CL*) and half‐life (*t_1/2_*) were determined to be 31 427 mL^−1^ h^−1^ kg^−1^ and 0.11 h, respectively. After oral dosing with 10 mg kg^−1^, the compound exhibited rapid absorption with a time‐to‐maximum concentration (*T_max_*) of 0.08 h, a favorable *t_1/2_* (3.55 h), a maximum concentration (*C_max_*) of 40.6 ng mL^−1^ (0.0786 µM), and an area under the curve (AUC_0‐t_) of 41.3 ng·h mL^−1^. It is worth noting that compared to the lead compound **GC‐14**, **C5N17B** exhibited significantly increased oral bioavailability reaching up to 13%, whereas **GC‐14** only achieved 7.2%.^[^ ^13^ ^]^ Notably, **C5N17B** displayed a substantially longer half‐life than **GC‐14** (1.72 h). These results indicate that **C5N17B** exhibits satisfactory oral half‐life and bioavailability which support our design concept for developing effective antiviral therapy using diazabicyclooctane derivatives bearing triazole groups.

**Table 5:** Pharmacokinetic parameters of **C5N17B**.

Parameters^a)^	Unit	*i.v*.^b)^ [C5N17B]	*p.o*.^c)^ [C5N17B]
		Mean ± SD	Mean ± SD
*t_1/2_*	h	0.106 ± 0.00569	3.55 ± 1.2
*T_max_*	h	0.083 ± 0.000	0.0833
*C_max_*	ng mL^−1^	237 ± 53.6	40.6 ± 24.1
*C_0_*	ng mL^−1^	514 ± 164.7	–
AUC_0‐t_	hr·ng mL^−1^	63.6 ± 14.2	41.3 ± 9.50
AUC_0‐∞_	hr·ng mL^−1^	65.9 ± 14.9	71.9 ± 26
MRT_0‐t_	h	0.0969 ± 14.9	1.79 ± 0.145
MRT_0‐∞_	h	0.116 ± 0.00652	5.00 ± 1.8
*CL*	mL^−1^ hR^−1^ kg^−1^	31 427 ± 7161	–
*F*	%	–	13

^a)^ PK parameters (mean ± SD, *n =* 3);

^b)^ Dosed intravenously at 2 mg kg^−1^;

^c)^ Dosed orally at 10 mg kg^−1^.

**Figure 9:**
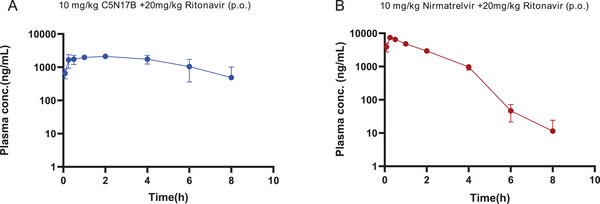
A) Plasma concentration−time curve of **C5N17B** following oral administration (p.o., 10 mg kg^−1^ in combination with 20 mg kg^−1^ Ritonavir) in ICR mice. B) Plasma concentration−time curve of **Nirmatrelvir** following oral administration (p.o., 10 mg kg^−1^ in combination with 20 mg kg^−1^ Ritonavir) in ICR mice.

Furthermore, in order to comprehensively investigate the pharmacokinetic properties of **C5N17B**, we conducted a comparative analysis between **C5N17B** and the FDA‐approved drug nirmatrelvir (**Table** **6**). Considering that nirmatrelvir requires co‐administration with the CYP3A4 enzyme inhibitor ritonavir to reduce its extensive metabolism, we compared the PK properties of **C5N17B** and nirmatrelvir at equivalent dose levels following co‐administration with ritonavir. After oral administration of either 10 mg kg^−1^ **C5N17B** or nirmatrelvir combined with 20 mg kg^−1^ ritonavir, their plasma concentration curves are depicted in **Figure** **9**. The use of a pharmacokinetic stabilizer resulted in a significant increase in plasma exposure for **C5N17B** (AUC_0‐t_ = 11542 ng·h mL^−1^), nearly 300‐fold higher than when administered alone (AUC_0‐t_ = 41.3 ng·h mL^−1^), and comparable to nirmatrelvir (AUC_0‐t_ = 14565 ng·h mL^−1^). It is worth highlighting that the half‐life of **C5N17B** (*t_1/2_* = 3.14 h) exceeds that of nirmatrelvir (*t_1/2_* = 0.692 h), indicating slower metabolism and elimination in vivo, and thereby suggesting possibly prolonged efficacy.

**Table 6:** Pharmacokinetic parameters of **C5N17B** and **Nirmatrelvir** in combination with ritonavir.

Parameters^a)^	Unit	*p.o*.^b)^ [C5N17B + ritonavir]	*p.o*.^b)^ [Nirmatrelvir+ ritonavir]
		Mean ± SD	Mean ± SD
*t_1/2_*	h	3.14 ± 2.06	0.692 ± 0.204
*T_max_*	h	2.08 ± 1.88	0.250 ± 0.000
*C_max_*	ng mL^−1^	2297 ± 162	7450 ± 715
AUC_0‐t_	hr·ng mL^−1^	11 542 ± 1728	14 565 ± 1008
AUC_0‐∞_	hr·ng mL^−1^	14 170 ± 4174	14 579 ± 1006
MRT_0‐t_	h	3.15 ± 0.456	1.55 ± 0.051
MRT_0‐∞_	h	4.88 ± 2.70	1.55 ± 0.055

^a)^ PK parameters (mean ± SD, *n =* 3);

^b)^ Dosed orally at 10 mg kg^−1^ **C5N17B** and **Nirmatrelvir** in combination with 20 mg kg^−1^ ritonavir.

In future studies, enhancing oral bioavailability can be achieved through suitable formulation strategies. The favorable pharmacokinetic properties of **C5N17B** strongly support its potential as an orally administered antiviral therapy, warranting further development.

### 2.9 Assessment of Acute and Subacute Toxicity

**Figure 10:**
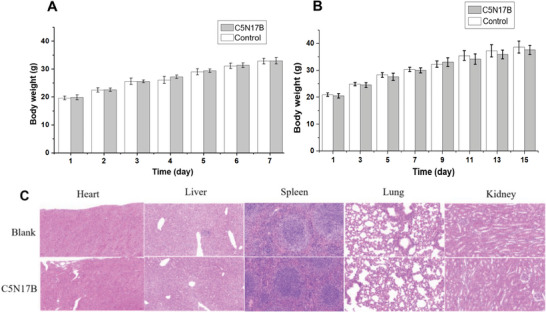
Visual presentation of in vivo toxicity experiment results for **C5N17B**. A) Time courses of body weight in the 7‐day acute toxicity experiment. B) Time courses of body weight in the 15‐day subacute toxicity experiment. C) Microscopic images of organ slices from mice treated in subacute toxicity study. The heart, liver, spleen, lung, and kidney were sectioned and stained with hematoxylin and eosin.

The acute and subacute toxicity of **C5N17B** were assessed in Kunming mice. In the acute toxicity study, one group received an oral dose of 500 mg kg^−1^ of **C5N17B**. In the subacute toxicity study, a different group was administered a lower dose of 50 mg kg^−1^ of **C5N17B**. Each experimental set included a control group that received only the vehicle treatment. No fatalities or abnormal behavior were observed in either the acute or subacute toxicity experiments with **C5N17B**. Additionally, there were no significant changes in body weight compared to the control group during 7 or 15 days of administration (**Figure** **10**A,B). Examination of vital organs using hematoxylin and eosin‐stained slices revealed no significant pathological abnormalities following the administration of **C5N17B** when compared to the vehicle control group (Figure 10C). This finding is consistent with the absence of cytotoxicity observed in vitro for **C5N17B** (CC_50_ >100 µM; Table 4), highlighting a favorable advantage in the compound's further progress toward clinical development.

## 3 Summary and Prospect

The global spread of SARS‐CoV‐2 and its variants underscores the urgent need for effective broad‐spectrum agents. In the context of antiviral drug development targeting M^pro^, several small‐molecule drugs have been developed, but only nirmatrelvir is currently FDA‐approved. Despite its approval, concerns regarding resistance and safety continue to challenge its clinical use. Although M^pro^ mutations resistant to nirmatrelvir are not yet prevalent in circulating viruses, such resistant viruses have been observed in experiments and clinical applications.^[^ ^37^ ^]^ Therefore, there remains an immediate demand for novel small molecule agents targeting SARS‐CoV‐2 M^pro^, with broad‐spectrum antiviral activity against a wider range of strains including drug‐resistant variants.

In the present study, we demonstrate the effectiveness of a modular methodology, (namely, microscale synthesis and direct screening in 96‐well plates), chemical re‐synthesis of selected molecules, and in‐depth evaluation, for discovering SARS‐CoV‐2 M^pro^ inhibitors. Using this approach, we constructed focused libraries through privileged fragment assembly and microscale synthesis technology based on CuAAC click chemistry, followed by direct biological screening. This led to the identification of 16 hit compounds featuring a novel diazabicyclooctane scaffold bearing a 1,2,3‐triazole moiety, which demonstrated remarkable enzyme inhibitory activity and antiviral efficacy at the cellular level. After chiral resolution and further evaluation, the (*R*)‐enantiomer **C5N17B** (IC_50_ = 0.120 µM, EC_50_ = 0.0780 µM) exhibited significant enzyme inhibition and antiviral activity in Calu‐3 cells, which was ≈19‐fold and 2‐fold more potent than **GC‐14** (IC_50_ = 0.800 µM, EC_50_ = 1.42 µM) and **AA‐625** (IC_50_ = 0.900 µM, EC_50_ = 0.150 µM). Importantly, **C5N17B** showed much higher activity than nirmatrelvir (IC_50_ = 0.0910 µM, EC_50_ = 1.95 µM) and was comparable to the non‐peptidic, non‐covalent M^pro^ inhibitor ensitrelvir (EC_50_ = 0.110 µM). None of the compounds in this series exhibited apparent cytotoxicity toward Vero cell and Calu‐3 cell lines(CC_50_ >100 µM). Furthermore, we demonstrated that **C5N17B** also showed significant antiviral activity against nirmatrelvir‐resistant SARS‐CoV‐2 strains in Calu‐3 cells (EC_50_ = 1.47 µM against the T21I/E166V variant and EC_50_ = 1.00 µM against the L50F/E166V variant), surpassing the efficacy of nirmatrelvir (EC_50_ > 100 µM). It is noteworthy that **C5N17B** exhibited broad‐spectrum properties against various coronaviruses, strongly inhibiting newly emerging SARS‐CoV‐2 variants as well as the human coronavirus HCoV‐OC43. In addition, **C5N17B** displayed a favorable pharmacokinetic profile, particularly in the presence of ritonavir. Overall, **C5N17B** shows great potential as a promising candidate for further development as an effective antiviral against SARS‐CoV‐2.

In conclusion, our study illuminates that the integration of miniaturized synthesis, direct screening of focused libraries, and crystallographic studies provides a powerful and universal platform for the rapid discovery of promising drug candidates. We envision that the further application of new types of click chemistry or modular reactions will open up new opportunities for this approach.

## 4 Experimental Section

### SARS‐CoV‐2 Main Protease Enzymatic Assay

The SARS‐CoV‐2 M^pro^/3CL^pro^ inhibitor screening kit utilizes the FRET method. The detection principle was as follows: Edans serves as the fluorescence donor, while Dabcyl acts as the fluorescence acceptor or quencher. These two fluorescent groups had overlapping absorption spectra, and when their distance was appropriate (typically 7 – 10 nm), fluorescence energy transferred from the donor to the acceptor, resulting in a reduction in the intensity of the donor's fluorescence signal. Edans and Dabcyl were linked to both ends of a natural substrate sequence of 2019‐nCoV M^pro^/3CL^pro^ protease, namely Dabcyl‐KTSAVLQSGFRKME‐Edans. Through fluorescence detection, it becomes feasible to sensitively measure the enzymatic activity exhibited by 2019‐nCoV M^pro^/3CL^pro^ protease.^[^ ^38^ ^]^

The SARS‐CoV‐2 M^pro^ inhibitor screening kit (Beyotime, Beijing) was utilized to assess the inhibitory potency of the target compounds on M^pro^. The concentrated M^pro^ was diluted 93‐fold with assay buffer, while the test compounds were prepared in various concentrations using DMSO and assay buffer. Initially, each well was added with 93 µL of diluted M^pro^ solution, followed by the immediate addition of 5 µL of test compound solution, and 2 µL of DMSO solution containing the fluorescent substrate (Dabcyl‐KTSAVLQSGFRKME‐Edans). Subsequently, under dark conditions, the plate was incubated for 5 min at 37 °C with a shaking speed of 90 rpm. The fluorescence intensities (RFU, Relative Fluorescence Unit) at a single time point of each well were then measured using a Spectramax iD5 plate reader (Molecular Devices) with an excitation wavelength of 340 nm and an emission wavelength at 490 nm. Simultaneously, a positive control, as well as a blank control (without enzyme), were set (Table S6, Supporting Information). According to the reading results, the inhibition rate for each sample was calculated using the following formula:

(1) $$\begin{array}{ccc} & & \mathrm{Inhibition}\;\left(\%\right)=\left(\mathrm{RF}U_{100\%\;\mathrm{enzyme}\;\mathrm{activity}\;\mathrm{control}}-\mathrm{RF}U_{\mathrm{sample}}\right)\\ & & /\left(\mathrm{RF}U_{100\%\;\mathrm{enzyme}\;\mathrm{activity}\;\mathrm{control}}-\mathrm{RF}U_{\mathrm{blank}\;\mathrm{control}}\right)\times 100\% \end{array}$$

The compounds were preliminarily screened at a concentration of 5 or 10 µM. Compounds surpassing the positive control's inhibition rates undergo secondary screening. For compounds exhibiting significant activity, inhibition rates were measured under seven concentrations (0.01, 0.05, 0.1, 0.5, 1, 5, 10 µM). IC_50_ values were calculated using GraphPad Prism 8. Each sample was tested in triplicate, from which the average value and standard deviation were calculated.

### SARS‐CoV‐2 M^pro^ Inhibitors K_i_ Assay

The Cheng–Prusoff equation *K*_i_ = IC_50_/(1+[S]/*K*_M_) was transformed into IC_50_ = *K*_i_ (1+[S]/*K*_M_), indicating a first‐order relationship between IC_50_ and substrate concentration. A Tris‐HCl solution (20 mm, pH = 7.5 at 37 °C), containing 1 mm EDTA and 0.01% BSA, was prepared as the assay buffer. Dabcyl‐KTSAVLQSGFRKME‐Edans served as the substrate. IC_50_ values were calculated at different substrate concentrations of 20, 50, 100, and 200 µM. Under a system containing a 1.5 µM final concentration of M^pro^, IC_50_s of inhibitors were measured under the above concentrations of the substrate. Under the same condition and substrate, it was reported that the *K*_M_ value for the wild‐type SARS‐CoV‐2 M^pro^ was 14.92.^[^ ^29^ ^]^ A linear regression equation was established using the measured IC_50_ values and (1+[S]/*K*_M_), in which the slope represents *K*_i_.

### Cells and Viruses

Human lung epithelial (Calu‐3) cells, human hepatocarcinoma (Huh7) cells, human lung fibroblast (MRC‐5) cells, and Rhesus monkey kidney (Vero 81) cells, and LLC‐MK2 cells were acquired from the American Type Culture Collection (ATCC). Calu‐3 cells were cultured in Eagle's minimum essential medium (EMEM; Corning) supplemented with 10% fetal bovine serum (FBS; Atlas Biologicals), while Huh7, MRC‐5, and LLC‐MK2 cells were maintained in Dulbecco's modified Eagle medium (DMEM; Cytiva, Hyclone) supplemented with 10% FBS. SARS‐CoV‐2 hCoV‐19/Korea/KCDC06/2020 (wild type), and its variants, including hCoV‐19/Korea/KDCA95637/2021 (gamma variant), hCoV‐19/Korea/KDCA119861/2021 (delta variant) and hCoV‐19/Korea/KDCA447321/2021 (omicron variant), were supplied by the Korea Disease Control and Prevention Agency (KCDA). Recombinant hCoV‐19/Korea/KCDC06/2020 expressing green fluorescent protein (GFP) fused at the N‐terminus of nucleocapsid protein, along with its nirmatrelvir‐resistant strains with T21I+E116V or L50F+E116V mutations in M^pro^, were generated using reverse genetics system based on circular polymerase extension reaction (CPER), following established methods.^[^ ^19^, ^39^, ^40^, ^41^ ^]^ This process yielded wild‐type rgSARS‐CoV‐2 and mutant rgSARS‐CoV‐2‐3CL/T21I+E166V and rgSARS‐CoV‐2‐3CL/L50F+E166V. Human isolates of Middle East respiratory syndrome coronavirus (MERS‐CoV; MERS‐CoV‐KOR/KNIH/002_02_2015) and alphacoronavirus NL63 (HCoV‐NL63) were obtained from KCDA, while human betacoronavirus OC43 (HCoV‐OC43) and alphacoronavirus 229E (HCoV‐229E) were provided by ATCC. All experiments involving infectious SARS‐CoV‐2s and MERS‐CoV were conducted in the BSL‐3 facility in KRICT.

### Immunofluorescence‐based Antiviral Assay

The antiviral assay against SARS‐CoV‐2 was performed following the previous report.^[^ ^42^ ^]^ Calu‐3 cells were seeded in 96‐well plates with a density of 1 × 10^5^ cells per well. The following day, the cells were infected with SARS‐CoV‐2 (hCoV‐19/Korea/KCDC06/2020) or its variants at a multiplicity of infection (MOl) of 0.01 while being exposed to increasing concentrations of tested compounds. On the second day after infection, the cells underwent fixation and permeabilization for immuno‐staining using an anti‐spike (S) antibody from Genetex and Alexa Fluor‐488 conjugated goat anti‐mouse IgG from Invitrogen. In the case of the antiviral assay involving reverse genetically rescued SARS‐CoV‐2s, viral infection was assessed by measuring the expression level of GFP. Nuclei staining was performed using Vectashield containing 4,6‐diamidino‐2‐phenylindole (DAPI; Vector Laboratories).^[^ ^43^ ^]^ The concentration required to reduce the number of cells expressing S or GFP by half was determined as the fifty percent effective concentration (EC_50_).

### Cytopathic Effect (CPE)‐based Antiviral Assay

The CPE inhibition assay was conducted following the previously published report.^[^ ^44^ ^]^ In brief, Huh7 cells, MRC‐5 cells, and LLC‐MK2 cells were individually seeded onto 96‐well plates at a density of ≈2 × 10^4^ cells per well. The following day, these cell lines were infected with various coronaviruses, including MERS‐CoV (MOI, 0.1 for Huh7 cells), HCoV‐OC43 (MOI, 0.01 for MRC‐5 cells), HCoV‐229E (MOI, 0.01 for MRC‐5 cells), and HCoV‐NL63 (MOI, 0.1 for LLC‐MK2 cells), in the presence of increasing concentrations of compounds. Infection‐induced CPE was quantified on day 2 (for MERS‐CoV), 5 (for HCoV‐NL63 and or HCoV‐229E) and 6 (for HCoV‐OC43) post‐infection by a cell viability assay using 3‐(4,5‐dimethylthiazol‐2‐yl)−2,5‐diphenyltetrazolium bromide (MTT; Sigma‐Aldrich). The EC_50_ values were determined by estimating the compound concentration required to improve the viability of virus‐infected cell lines by half.

### Cytotoxicity Assay

For the cytotoxicity assessment, mock‐infected Calu‐3 cells were treated with the compounds employed in the antiviral assays above. On day 2 (for Calu‐3, Vero, and Huh7 cells), 5 (for MRC‐5 and LLC‐MK2 cells), and 6 (for MRC‐5 cells), cell viability was measured using MTT after cell lysis. The fifty percent cytotoxic concentration (CC_50_) was defined as a compound concentration that reduces cell viability by half compared to mock‐treated cells. The selectivity index (S.I.) represents the ratio of CC_50_ to EC_50_ obtained from the same compound incubation period.^[^ ^42^ ^]^

### Western Blot Analysis

Calu‐3 cells were cultured in 48‐well plates at a density of 1 × 10^5^ cells per well for 2 days. Following a 2 h infection with SARS‐CoV‐2 at an MOI of 0.01 at 37 °C and subsequent removal of the unabsorbed virus using PBS washing, the cells were treated with increasing concentrations of each compound diluted in FBS‐free fresh EMEM. After 48 h post‐infection, cell lysates were collected using M‐PER buffer (Thermo Scientific). The viral proteins were detected using a primary anti‐S antibody (Genetex) or anti‐N antibody (Sino Biological), followed by secondary horseradish peroxidase (HRP)‐conjugated anti‐mouse goat IgG (Invitrogen). Cellular β‐actin was used as the loading control.^[^ ^42^ ^]^

### Quantitative RT‐PCR

Calu‐3 cells were exposed to SARS‐CoV‐2, and subsequently treated with various concentrations of each compound as mentioned just above. On day 2 post‐infection, culture supernatants were collected for viral RNA purification using the QIAamp viral RNA mini kit from Qiagen. The quantification of the SARS‐CoV‐2 RNA load was performed using a real‐time RT‐PCR kit with an N gene‐specific primer set (PCL Inc.) and a CFX96 Touch real‐time PCR instrument (Bio‐Rad).^[^ ^42^ ^]^

### Expression and Purification of SARS‐CoV‐2 M^pro^ for Crystallization

A plasmid was used for SARS‐CoV‐2 M^pro^ expression as previously described.^[^ ^45^ ^]^ *E.coli* BL21 (DE3) cells were transformed with the plasmid and single colonies were picked for pre‐culture inoculation in LB media. The cultures were grown at 37 °C in an auto‐induction medium (Table S5, Supporting Information) until the optical density value reached 0.8, and then incubated at 18 °C overnight. The cells were harvested by centrifugation and the cell pellets were stored at −80 °C.

Cells were re‐suspended in binding buffer A (20 mm Tris, 150 mm NaCl, and 10 mm imidazole, pH 7.8) and lysed by homogenization using a FastPrep‐24 5G with 0.1 mm diameter zirconia/silica beads. After centrifugation at 48 400 g for 30 min, the supernatant was applied to a HiTrap TALON crude 5 mL column. After a wash step with 150 ml buffer A, the bound protein was eluted with a linear gradient in 30 column volumes to 60% of buffer B (20 mm Tris, 150 mm NaCl, and 500 mm imidazole, pH 7.8). The His‐tag at the C‐terminus of M^pro^ was removed by HRV 3C protease (Pierce) at an enzyme‐substrate ratio of 1:50 (w/w). The solution was dialyzed at 4 °C against dialysis buffer A (20 mm Tris, 150 mm NaCl, and 1 mm DTT, pH 7.8) for 16 h and then transferred into dialysis buffer B (20 mm Tris and 150 mm NaCl, pH 7.8) for 2 h. The HRV 3C protease and M^pro^ with uncleaved His‐tag were removed by a HiTrap TALON crude 5 mL column using the same procedure as described before. Fractions containing M^pro^ were pooled and concentrated to ≈3 mL by ultrafiltration. The protein was applied to a HiLoad 16/600 Superdex 200 pg column and eluted with gel filtration buffer (20 mm Tris, 15 mm NaCl, 1 mm TCEP, and 1 mm EDTA, pH 7.8). The pooled fractions were concentrated to 5 mg mL^−1^ and immediately flash‐frozen by liquid nitrogen for storage at −80 °C.

### Crystallization of the SARS‐CoV‐2 M^pro^

Apo M^pro^ crystals were obtained by hanging drop vapor diffusion method with 23.5–24% PEG1500, 0.2 m MIB pH 7.8 (sodium malonate, imidazole, boric acid with molar ratios 2:3:3), 5% DMSO, 1 mm DTT, and 0.25 mm EDTA as the reservoir solution. The crystallization droplet consisted of 2 µL protein solution (5 mg mL^−1^), 1 µL reservoir, and 0.5 µL crystal seeds. The seed stock of M^pro^ crystal in the desired P2_1_2_1_2_1_ space group described below was kindly provided by Laila Benz and Manfred Weiss (Helmholtz‐Zentrum Berlin). Seed crystals were in a buffer containing 23.5% PEG1500, 0.2 m MIB pH 7.7, 5% DMSO, 1 mm DTT, and 0.25 mm EDTA. Crystals grew to their final size within a week. The crystals were soaked with 1.5 mm of the inhibitors for ≈5 min (**C5N17A**) to 24 h (**C5N17B**). The crystals were shock‐cooled in liquid nitrogen without adding further cryo‐protectants.

### Data Collection and Refinement

The crystals were soaked with 1.5 mm of the inhibitors for ≈5 min to 24 h. The crystals were shock‐cooled in liquid nitrogen without adding further cryo‐protectants. X‐ray diffraction data were collected at 100 K at EMBL beamline P13 at the DESY synchrotron in Hamburg, Germany (Table S4, Supporting Information). The diffraction data were indexed, integrated, and scaled with XDS^[^ ^46^ ^]^ and STARANISO^[^ ^47^ ^]^ as implemented in ISPyB^[^ ^48^ ^]^ at DESY. The structure 7 mbg^[^ ^49^ ^]^ was used as a starting model for refinement. In both structures, the inhibitors had bound with full occupancy to only chain A of the two protein chains in the asymmetric unit. This was due to the disorder of residues Ser46 to Leu50 in chain B of this crystal, which was caused by different crystal packing interactions. Met49 forms part of the inhibitor binding pocket. Weak difference electron density indicates low occupancy binding of **C5N17B** to chain B. Phenix^[^ ^50^ ^]^ was used for refinement and Coot^[^ ^51^ ^]^ for model building. Stereochemical restraints for ligand refinement were generated using grade 2 (https://grade.globalphasing.org). Molecular Figures were generated using PyMOL (https://pymol.org).

### In vivo Pharmacokinetic Study

A total of six male ICR mice were randomly assigned to two groups. One group received the test drug intravenously at a dosage of 2 mg kg^−1^, while the other group received oral administration at a dosage of 10 mg kg^−1^. To prepare solutions of **C5N17B**, 2.39 mg compound was dissolved in a mixture of 0.119 mL DMSO, 0.238 mL polyoxyl 15 hydroxystearate (Solutol), and 2.027 mL normal saline. For the intravenous group, blood samples were collected from the sinus jugular into heparinized centrifugation tubes at various time points after dosing: 5, 15, 30 min, 1, 2, 4, 6, 8, and 24 h. As for the oral administration group, blood samples were collected at similar time points (0.05 mL of blood each time). The collected blood samples were then centrifuged at a speed of 4000 rpm for 10 min to separate plasma, which was subsequently stored at −20 °C until further LC‐MS analysis to determine the concentration of **C5N17B**. Briefly, the analysis involved adding 20 µL of plasma, sample calibration standard, quality control, and dilution quality control, and blank samples into respective wells on a 96‐well plate. Then each sample was quenched with 200 µL of internal standard (the blank sample was quenched with 200 µL of ACN: MeOH = 1:1), followed by vortex‐mixed for 5 min, and centrifuged for 0.5 min at 4000 rpm, 4 °C. Next, a 100 µL portion of supernatant was transferred to another clean 96‐well plate and diluted with 100 µL of water, vortex‐mixed for 5 min and centrifuged for 10 min at 4000 rpm, 4 °C, then the sample was injected for LC‐MS/MS analysis. All samples were quantified using an LC‐MS/MS‐AR Triple Quad 5500+ (SCIEX, USA) and the ExionLC liquid phase system. The mobile phase was 0.1% formic acid‐water/ACN with gradient elute at a flow rate of 0.8 mL mi^−1^n (total time 2.20 min), and the detection wavelength was 225 nm. All blood samples were centrifuged using an Eppendorf 5424R centrifuge and quantified using an LC‐MS/MS‐AR Triple Quad 5500+ (SCIEX, USA). The pharmacokinetic parameters were calculated by WinNolin 8.2 software.

### Acute and Subacute Toxicity Experiment

Kunming mice were group housed both during acclimation and the study period. The animal room environment was controlled to maintain specific target conditions, including a temperature range of 20 °C to 25 °C, relative humidity levels between 40% and 70%, and an alternating schedule of artificial light for 12 h followed by 12 h of darkness. The age of Kunming mice in the acute/subacute toxicity study was 3–4 weeks, and the body weights were ≈20 g. All animal experiments related to this study were approved by the ethics committee of Cheeloo College of Medicine (approval No. 230014), Shandong University (Jinan, China) Procedures performed in the studies were guided by the ethical standards of the institution.

A group of Kunming mice (comprising three males and three females) was supplied by the Animal Experimental Center of Shandong University. The mice underwent a 12 h fasting period, followed by oral administration of a suspension containing **C5N17B** in 0.5% CMC‐Na and 3% DMSO at the concentration of 100 mg mL^−1^ to achieve an acute toxicity dose of 500 mg kg^−1^. In the subacute toxicity experiment, the test group received 50 mg kg^−1^ of **C5N17B** orally, with another group serving as a control. The experimental group and control group each consist of six mice, with an equal distribution of three males and three females.

### Statistical Analysis

The majority of the experiments were replicated biologically three times to ensure reliability. Data from these multiple runs were analyzed using GraphPad Prism 8 and were presented as the mean standard deviation (± SD). To assess the statistical significance of the findings, Student's t‐tests using Microsoft Excel's built‐in statistical tools or GraphPad Prism. P value < 0.05 was considered statistically significant.

## Conflict of Interest

The authors declare no conflict of interest.

## Author Contributions

M.Y., M.K.L., S.G., and L.S. contributed equally to this work. P.Z., M.K., X.L., M.Y., and L.S. conceived the project. M.Y. and S.G. synthesized the compounds. M.Y. and L.S. constructed the click‐based compound libraries and enzyme assays. M.Y. designed the experiments and analyzed the data. M.K.L. and C.K. performed the antiviral and cytotoxicity assays and analyzed the data. H.‐Y.J. reverse genetically generated wild‐type and nirmatrelvir‐resistant mutant SARS‐CoV‐2 strains. M.K.L. and I. J. performed molecular virological analysis. K.S., C.Y., N.S., and C.E.M. conducted X‐ray structure data collection and analysis. S.W. conducted the molecular dynamics simulation. B.Y., K.T., J.L., and M.G. assisted in confirming the structure of the compounds. M.Y. wrote the manuscript and everybody contributed to revising it.

## Supporting information



Supporting Information

Supporting Information

## Data Availability

The data that support the findings of this study are available in the supplementary material of this article.
