# Supplementary material for: Miniaturized Modular Click Chemistry‐enabled Rapid Discovery of Unique SARS‐CoV‐2 Mpro Inhibitors With Robust Potency and Drug‐like Profile
Source: Adv Sci (Weinh). 2024 Sep 25;11(43):2404884. doi: 10.1002/advs.202404884 (PMC11578313; doi:10.1002/advs.202404884)

**Supporting Information**

**Miniaturized Modular Click Chemistry-Enabled Rapid Discovery of Unique SARS-CoV-2 M^pro^ Inhibitors with Robust Potency and Drug-like Profile**

*Mianling Yang^+^,* *Myoung Kyu Lee^+^, Shenghua Gao^+^, Letian Song^+^, Hye-Yeon Jang, Inseong Jo, Chun-Chiao Yang, Katharina Sylvester, Chunkyu Ko, Shuo Wang, Bing Ye, Kai Tang, Junyi Li, Manyu Gu, Christa E Müller^*^, Norbert Sträter^*^, Xinyong Liu^*^, Meehyein Kim^*^, Peng Zhan^*^*

M. Yang, S. Gao, L. Song, S. Wang, B. Ye, K. Tang, J. Li, M. Gu, Prof. X. Liu, Prof. P. Zhan.

Department of Medicinal Chemistry, Key Laboratory of Chemical Biology, Ministry of Education, School of Pharmaceutical Sciences, Shandong University, Ji'nan, 250012, China.

E-mail address: zhanpeng1982@sdu.edu.cn (Zhan P.); xinyongl@sdu.edu.cn (Liu X.Y.).

M. K. Lee, H. -Y Jang, I. Jo, C. Ko, Dr. M. Kim.

Infectious Diseases Therapeutic Research Center, Korea Research Institute of Chemical Technology (KRICT), Daejeon, 34114, Republic of Korea.

E-mail address: mkim@krict.re.kr (Kim M.).

K.Sylvester, Prof. C. E. Müller.

PharmaCenter Bonn & Pharmaceutical Institute, Department of Pharmaceutical & Medicinal Chemistry, University of Bonn, An der Immenburg 4, 53113 Bonn, Germany.

E-mail address: christa.mueller@uni-bonn.de (Müller C.E.).

C.-C. Yang, Prof. N. Sträter.

Institute of Bioanalytical Chemistry, Leipzig University, Deutscher Platz 5, 04103 Leipzig, Germany.

E-mail address: strater@bbz.uni-leipzig.de (Sträter N.).

[*] Corresponding authors.

[+] These authors equally contribute to this study.

**Table of Contents**

1. Supplementary Figures and Tables...............................................................S4

Table S1. The detailed conditions of CuAAC reaction ............................................S4

Table S2. The IC_50_ values of **C5N17B**, nirmatrelvir and ensitrelvir under different concentrations of substrate ..........................................................................................S5

Table S3. The equation representing the fitted standard curve of **C5N17B**, nirmatrelvir and ensitrelvir...............................................................................................................S5

Table S4. Diffraction data and refinement statistics ...................................................S6

Table S5. Composition of the auto-induction medium used for protein expression ...S8

Table S6. Pipetting scheme for M^pro^ assays ................................................................S9

Figure S1. LC-MS result of crude **C5N5** ..................................................................S10

Figure S2. LC-MS result of crude **C5N6** ..................................................................S10

Figure S3. LC-MS result of crude **C5N16** ................................................................S11

Figure S4. LC-MS result of crude **C5N17** ................................................................S11

Figure S5. LC-MS result of crude **C5N21** ................................................................S12

Figure S6. LC-MS result of crude **C5N39** ................................................................S12

Figure S7. LC-MS result of crude **C5N58** ................................................................S13

Figure S8. Inhibition percentage of lib-C3 series at 5 μM ........................................S14

Figure S9. Inhibition percentage of lib-C4 series at 10 μM ......................................S14

Figure S10. Inhibition percentage of lib-C5 series at 10 μM ....................................S14

Figure S11. Inhibition percentage of lib-C6 series at 5 μM ......................................S14

Figure S12. The linear regression between the IC_50_ values and 1+[S]/*K*_M_. of three compounds (**C5N17B**, Nirmatrelvir, and Ensitrelvir). ..............................................S15

Figure S13. Binding modes of **C5N17** diastereomers ..............................................S15

Figure S14. RMSD-time profiles of protein-ligand complexes ................................S16

Figure S15. 3D structural analysis of SARS-CoV-2 M^pro^ complexed with **JZD-07** ...............................................................................................................................S18

Abbreviations ............................................................................................................S19

2. In Silico Study...................................................................................................S20

2.1 Molecular docking ..............................................................................................S20

2.2 Molecular dynamics simulation ..........................................................................S20

3. Experimental Section—Chemistry...............................................................S21

3.1 General information.............................................................................................S21

3.2 Supplementary schemes.......................................................................................S21

3.3 Chiral separation..................................................................................................S38

3.4 Experimental procedures and characterization data.............................................S39

3.4.1 NMR spectra of final compounds.....................................................................S39

3.4.2 HRMS spectra of final compounds...................................................................S59

3.4.3 HPLC chromatograms of final compounds......................................................S65

1. Supplementary Figures and Tables

**Table S1**. The Detailed Conditions of CuAAC Reaction.

| Reagents | Concentration | Volume | Final Concentration |
| --- | --- | --- | --- |
| Alkyne-unit(CX) | 25nM DMSO | 20 μL | 5 mM |
| Azide-unit(NX) | 35 nM DMSO | 20 μL | 7 mM |
| TBTA | 10 nM DMSO | 10 μL | 1mM |
| CuSO_4_·5H_2_O | 4 nM DMSO | 25 μL | 1 mM |
| Sodium ascorbate | 20 nM DMSO | 25 μL | 5 mM |
| Total Volume | 100 μL/well | | |

**Table S2**. The IC_50_ values of **C5N17B**, nirmatrelvir and ensitrelvir under different concentrations of substrate.

| **Compounds** | **Substrate concentration [S] (μM)** | **1+[S]/*K*_M_** | **IC_50_ (μM)** |
| --- | --- | --- | --- |
| C5N17B | 20 | 2.34 | 0.0668 ± 0.0080 |
|  | 50 | 4.35 | 0.115 ± 0.0090 |
|  | 100 | 7.70 | 0.209 ± 0.0060 |
|  | 200 | 14.4 | 0.386 ± 0.010 |
| Nirmatrelvir | 20 | 2.34 | 0.0396 ± 0.0040 |
|  | 50 | 4.35 | 0.0677 ± 0.0060 |
|  | 100 | 7.70 | 0.124 ± 0.0050 |
|  | 200 | 14.4 | 0.235 ± 0.0090 |
| Ensitrelvir | 20 | 2.34 | 0.0363 ± 0.0040 |
|  | 50 | 4.35 | 0.0728 ± 0.0028 |
|  | 100 | 7.70 | 0.143 ± 0.012 |
|  | 200 | 14.4 | 0.237 ± 0.0090 |

**Table S3**. The equation representing the fitted standard curve of **C5N17B**, nirmatrelvir and ensitrelvir.

| **Cpds.** | **Linear regression** | **R^2^** | ***K*_i_** |
| --- | --- | --- | --- |
| C5N17B | y = 0.0266x + 0.0025 | 0.9997 | 0.0266 |
| Nirmatrelvir | y = 0.0163x - 0.001 | 0.9995 | 0.0163 |
| Ensitrelvir | y = 0.0166x + 0.0025 | 0.9906 | 0.0166 |

**Table S4**. Diffraction data and refinement statistics

| **Compound** | **C5N17A** | **C5N17B** |
| --- | --- | --- |
| PDB entry ID | 9G0H | 9G0I |
| Final buffer | 1.5 mM inhibitor, 24% PEG1500, 0.05 M sodium malonate, 0.075 M imidazole, 0.075 M boric acid, 5 % DMSO, 1 mM DTT, 1.58 mM EDTA, 26.67 mM Tris, 20 mM NaCl, 1.33 mM TCEP, pH 7.8 | |
| ***Data collection*** |  |  |
| Source | DESY EMBL P13 | DESY EMBL P13 |
| Wavelength (Å) | 0.9762 | 0.9762 |
| Resolution (Å) | 72.08-1.65 (1.84-1.65) | 72.30-1.67 (1.80-1.67) |
| Resolution aniso (Å) | 1.635, 2.245, 1.831 | 1.630, 2.074, 1.766 |
| Space group | P2_1_2_1_2_1_ | P2_1_2_1_2_1_ |
| Unit cell dimensions (Å;°) | 67.89, 99.89, 104.10; 90.0, 90.0 90.0 | 67.83, 100.02, 104.64; 90.0, 90.0 90.0 |
| Unique reflections | 55044 (2752) | 61110 (3057) |
| Multiplicity | 13.5 (13.4) | 13.8 (13.4) |
| Completeness (%)*  spherical/ellipsoidal | 64.1 (11.7) / 95.4 (72.1) | 72.9 (17.3) / 93.8 (65.1) |
| Mean I/s(I) | 17.5 (1.7) | 14.9 (1.5) |
| R-meas | 0.092 (1.713) | 0.096 (1.691) |
| R-merge | 0.089 (1.648) | 0.093 (1.626) |
| R-pim | 0.025 (0.466) | 0.026 (0.454) |
| CC_1/2_ | 0.999 (0.659) | 0.999 (0.611) |
| Wilson B (Å^2^) |  |  |
| ***Refinement*** |  |  |
| Resolution (Å) | 46.16-1.65 (1.68-1.65) | 40.25-1.67 (1.69-1.67) |
| R-work | 0.1849 (0.2536) | 0.1856 (0.2600) |
| R-free | 0.2217 (0.3594) | 0.2327 (0.1781) |
| Number of non-hydrogen atoms, B-value (Å^2^) |  |  |
| Protein | 4744, 34.55 | 4769, 31.37 |
| Heterogen | 55, 55.53 | 62, 36.98 |
| Solvent | 367, 36.87 | 425, 36.72 |
| Rmsd bonds (Å), angles (°) | 0.005, 0.759 | 0.011, 1.085 |
| Ramachandran favored, allowed, outliers (%) | 98.15, 1.68, 0.17 | 98.99, 1.01, 0.00 |
| Rotamer outliers (%) | 1.69 | 2.04 |
| MolProbity clashscore | 4.10 | 4.48 |

*Anisotropic truncation has been used. The first line refers to the spherical and the second line to the ellipsoidal completeness.

**Table S5**. Composition of the auto-induction medium used for protein expression

| **Auto-induction medium** | **Compositions** | | |
| --- | --- | --- | --- |
| Medium | 1  0.5 | %  % | Tryptone  yeast extract |
| Carbon sources | 0.5  0.05  0.2 | %  %  % | Glycerol (v/v)  Glucose (w/v)  α-Lactose (w/v) |
| Additional salts | 25  25  50  5  2 | mM  mM  mM  mM  mM | Na_2_HPO_4_  KH_2_PO_4_  NH_4_Cl  Na_2_SO_4_  MgSO_4_ |
| Trace metals | 10  4  2  2  0.4  0.4  0.4  0.4  0.4 | µM  µM  µM  µM  µM  µM  µM  µM  µM | FeCl_3_  CaCl_2_  MnCl_2_  ZnSO_4_  CoCl_2_  NiCl_2_  Na_2_MoO_4_  Na_2_SeO_3_  H_3_BO_3_ |

**Table S6**. Pipetting scheme for M^pro^ assays

|  | Assay buffer | M^pro^ dilution solution | Inhibitor | Fluorescent substrate |
| --- | --- | --- | --- | --- |
| Test well | - | 93 μL | 5 μL | 2 μL |
| Blank well | 98 μL | - | - | 2 μL |
| Control well | 5 μL | 93 μL | - | 2 μL |

LC-MS results of crude products **C5N5**, **C5N6**, **C5N16**, **C5N17**, **C5N21**, **C5N39** and **C5N58**.

LC-MS trace of representative selected crude compounds showed that the limiting starting materials were almost completely consumed and the desired triazole products were quantitatively formed. Thus, we concluded that the click library was sufficient pure and the preliminary screening results were credible.

**Figure S1**. LC-MS result of crude **C5N5**.

**Figure S2**. LC-MS result of crude **C5N6**.

**Figure S3**. LC-MS result of crude **C5N16**.

**Figure S4**. LC-MS result of crude **C5N17**.

**Figure S5**. LC-MS result of crude **C5N21**.

**Figure S6**. LC-MS result of crude **C5N39**.

**Figure S7**. LC-MS result of crude **C5N58**.

Direct screening of crude product against the SARS-CoV-2 M^pro^


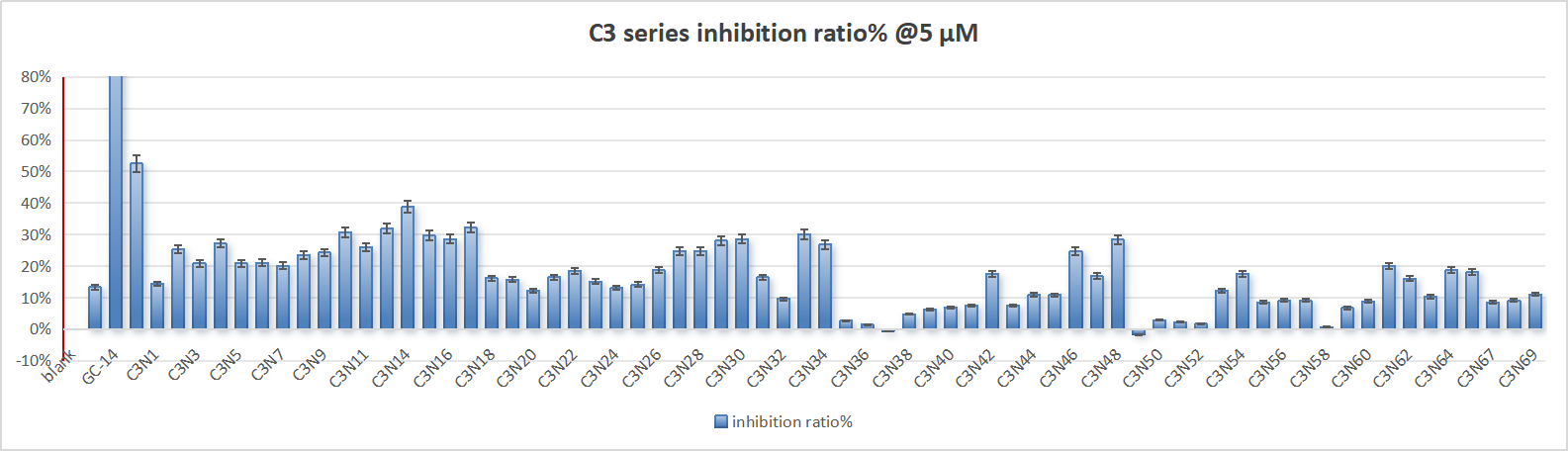


**Figure S8**. Inhibition percentage of lib-C3 series at 5 μM.


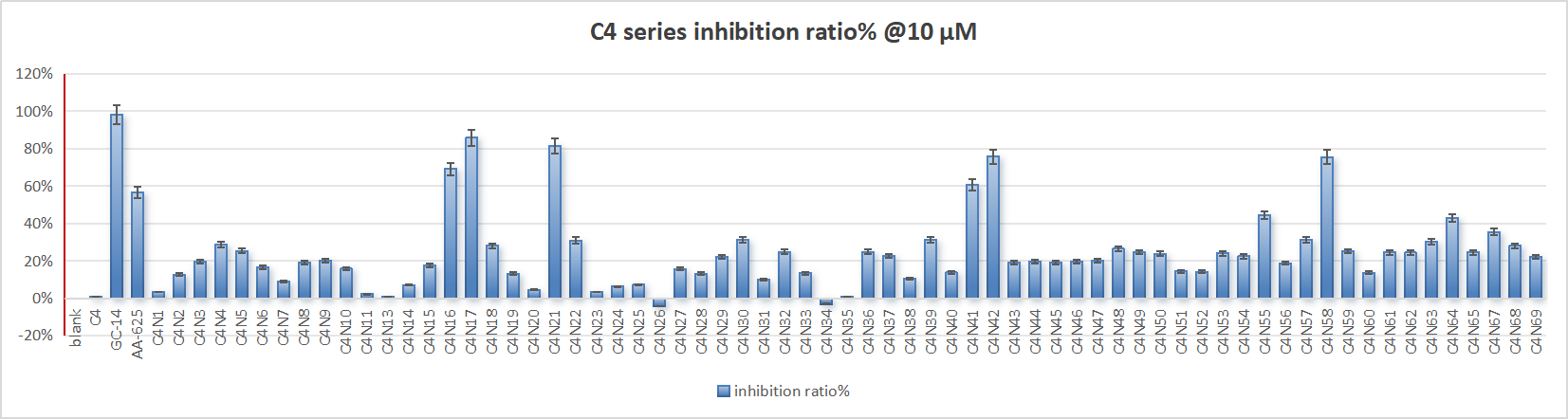


**Figure S9**. Inhibition percentage of lib-C4 series at 10 μM.


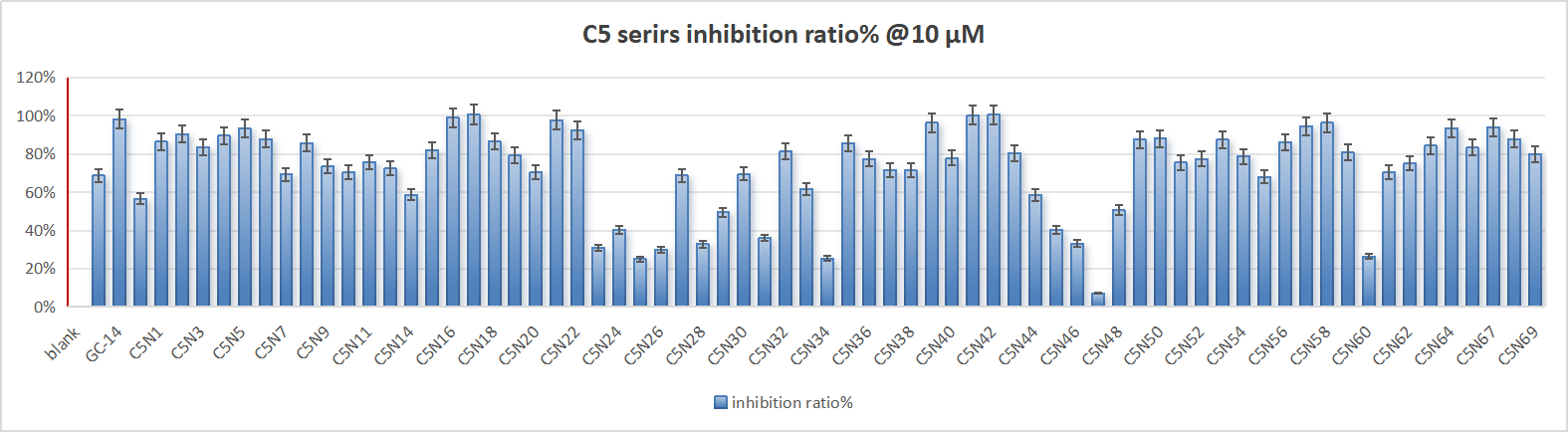


**Figure S10**. Inhibition percentage of lib-C5 series at 10 μM.


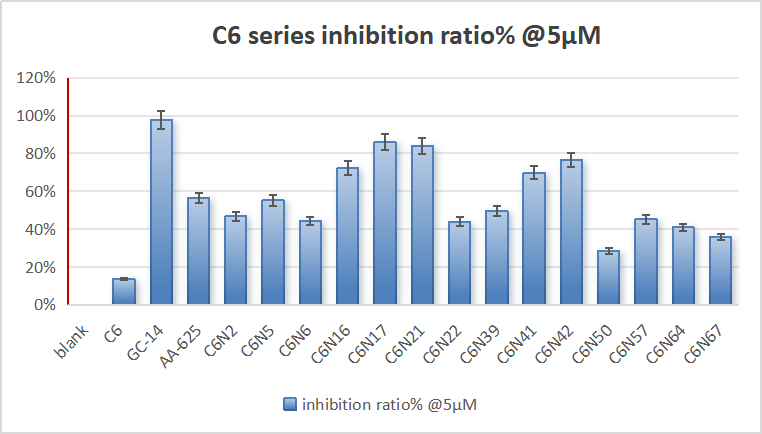


**Figure S11**. Inhibition percentage of lib-C6 series at 5 μM.


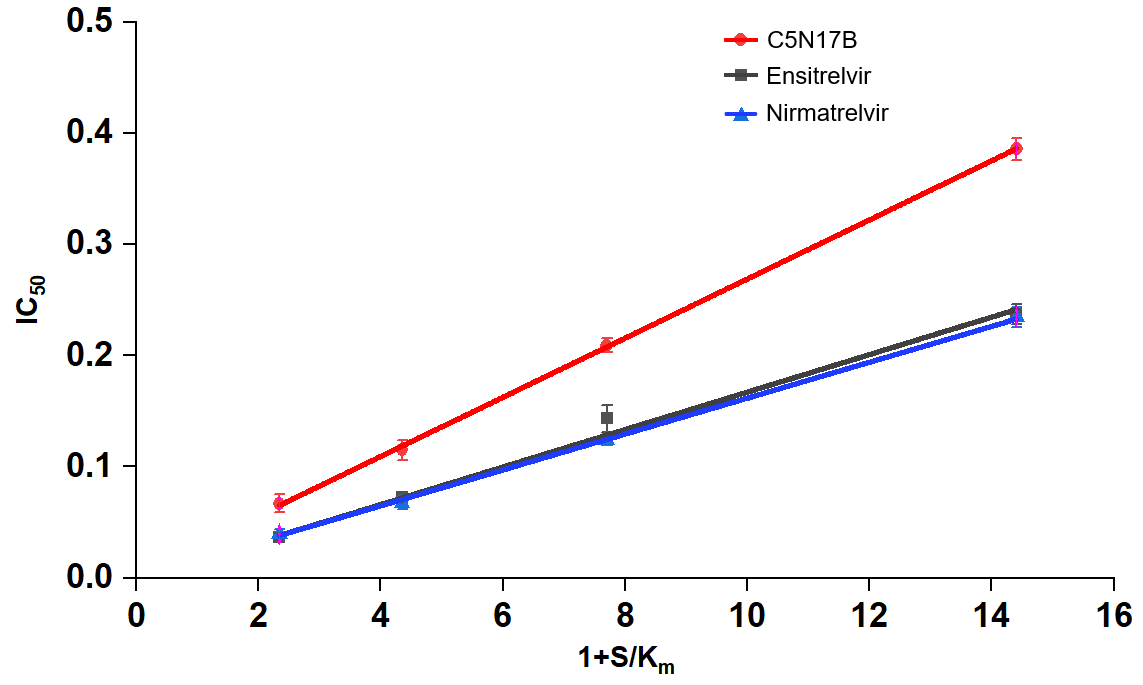


**Figure S12.** The linear regression between the IC_50_ values and 1+[S]/*K*_M_. of three compounds (**C5N17B**, Nirmatrelvir, and Ensitrelvir). [S] denotes the substrate concentration and K_M_ is the Michaelis constant.


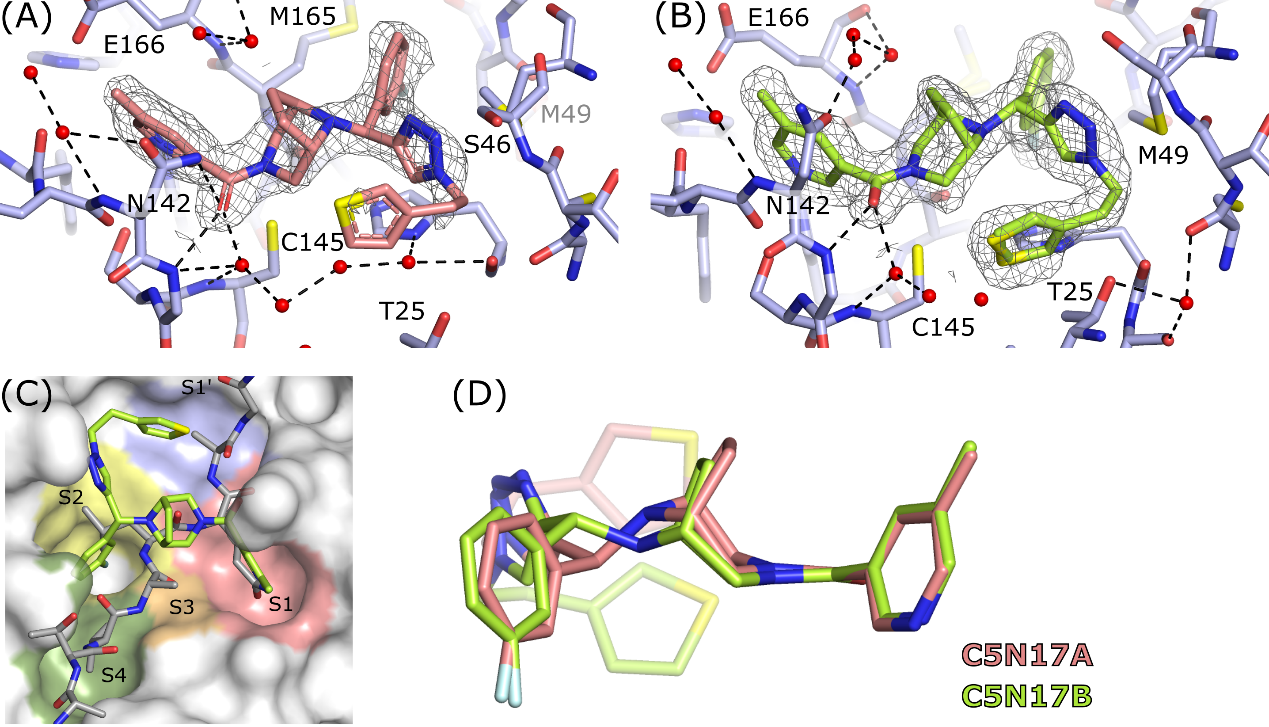


**Figure S13**. Binding modes of C5N17 diastereomers. The (2Fo-Fc)-type electron density map of **C5N17A** (A) and **C5N17B** (B) are shown at a contour level of 1 σ_rms_. The thiophene ring was refined in two alternative conformations. (C) Superposition of the binding modes of **C5N17B** (green) and the nsp9-nsp10 natural substrate peptide (grey, PDB ID: 7TA4)^S1^. The substrate binding pockets are presented in different colors. (D) Side-view of the superposition of **C5N17A** and **C5N17B**.

**Figure S14**. RMSD-time profiles of protein-ligand complexes with reference to the first frame for the **C5N17A**/SARS-CoV-2 M^pro^ complex (A) and **C5N17B**/SARS-CoV-2 M^pro^ complex (B) during the 250 ns MD simulations.

**Figure S15**. (A) The structure of another multi-substituted piperazine derivative, **JZD-07**. (B) X-ray structure of the complex of **JZD-07** and SARS-CoV-2 M^pro^ (PDB code: 8GTV). This figure was shown in Pymol 2.5

**Abbreviations**

AUC, area under curve;

AUC_0-t_, area under the concentration-time curve to the last measurable time point;

AUC_0-∞_, area under the concentration-time curve extrapolated to infinity;

COVID-19, coronavirus disease 2019;

*CL*, clearance rate;

*C_0_*, initial plasma concentration;

*C_max_*, maximum concentration;

DCM, dichloromethane;

DIPEA, *N*,*N*-diisopropanyl ethylamine;

FRET, fluorescence resonance energy transfer;

*F*, oral bioavailability;

HATU,*O*-(7-azabenzotriazol-1-yl)-*N*,*N*,*N*',*N*'-tetramethyluroniumhexafluorophosphate; HPLC, high performance liquid chromatography;

M^pro^, main protease;

MTT, methyl thiazolyl tetrazolium;

MRT_0-t_, mean residence time within the detection period;

MRT_0-∞_, mean residence time extrapolated to infinity;

SAR, Structure-Activity Relationship;

SARS-CoV-2, Severe Acute Respiratory Syndrome Coronavirus 2;

THF, tetrahydrofuran;

TLC, thin-layer chromatography;

*t_1/2_*, terminal half-life;

*T_max_*, time-to-maximum concentration;

TMS, tetramethyl silane.

1. In Silico Study

2.1 Molecular docking

The protein structures for computational simulation were downloaded from the Protein Database Bank (PDB). SARS-CoV-2 M^pro^ complex (PDB ID: 8ACL).

Molecular docking procedure: all the calculation processes were supported by the corresponding modules of Schrodinger 2021-4 suite (www.schrodinger.com accessed on 13 November 2022) and was performed on DELL Precision T5500 workstation. Firstly, compounds were optimized with the Ligprep model with default parameters, and a pair of chiral isomers are generated for each compound. The ionic state under the physiological condition of ligand (pH = 7.0) was added; OPLS4 force filed was selected to optimize and obtain the ligand molecules required for screening. The preparation of the protein was completed by the protein preparation wizard module. A series of processes such as hydrogenation, charging, elimination of conflicting amino acid residues and energy minimization of the protein crystal structure were carried out with default parameters Finally, the Glide module was used to dock the optimized ligands with the receptor protein with extra precision (XP)., According to the established docking model, Schrodinger 2021-4 Glide XP module was used for molecular docking of the compounds. The docking poses were visualized by Pymol (Schrödinger, LLC. DeLano Scientific, San Francisco, CA, USA, https://pymol.org accessed on 13 November 2022).

2.2 Molecular dynamics simulation

To further evaluate the binding stability of **C5N17A** and **C5N17B** towards SARS-CoV-2 M^pro^, RMSD and total energy (MM-GBSA) were predicted through MD simulation systems. The average position fluctuations of ligand and protein atoms, represented by RMSD value, were shown in Figure S13.

MD simulations were performed to further investigate the dynamic interactions between M^pro^, **C5N17A** and **C5N17B**. All simulations were conducted by using Schrodinger version 2021-4, and employed OPLS-4 force field. The co-crystal structure were employed as the starting coordinates, which was then filled into a proper box and solvated with water (TIP3P). The whole system was then added corresponding Na^+^ or Cl^-^ to neutralize all charges. Then 0.15 M NaCl was additionally added to simulate salt concentration under physiological condition. The whole system was relaxed with default set and the productive simulation was then performed for 250 ns under standard state (300 K, 1 bar). The result trajectories were then analyzed and the RMSD of the ligand was calculated.

1. Experimental Section—Chemistry

3.1 General information

All chemical reagents and solvents were purchased from commercial vendors and used as received. The reactions were routinely monitored by thin layer chromatography (TLC) on silica gel GF254. The purification of crude product were performed on a Biotage Isolera One system with prepacked columns. The melting points (m.p.) were determined on a micro melting point apparatus (RY-1G, Tianjin TianGuang Optical Instruments). ^1^H NMR and ^13^C NMR spectra were recorded in DMSO-*d_6_* /CDCl_3_ on a Bruker AV-600 or AV-400 spectrometer, with tetramethyl silane (TMS) as the internal standard. Coupling constants were given in hertz, and chemical shifts were reported in δ values (ppm) from TMS. The high resolution mass spectrometry (HRMS) spectra of representative compounds were performed on the LTQ Orbitrap XL (Thermo Fisher). The purity of representative final compounds was assayed on a Agilent 1260 prim HPLC system. HPLC conditions: Agilent ZORBAX, SB-C18 column (250 mm × 4.6 mm × 5 μm). Mobile phase A was methanol, while mobile phase B was 0.1% formic acid in water; The HPLC gradient elution was set as follows: Time (min)/% of solvent B: 0/30, 2/30, 10/95, 13/90, 13.1/30, 15/30; flow rate 1.0 mL/min; wavelength: 254 nM , temperature, 30 °C; injection volume, 10 μL.

3.2 Supplementary schemes

The general synthetic steps adopted to obtain the alkynes C4 ~ C6 are outlined in **Schemes 1-2**. As depicted in **Scheme 1**, starting with raw materials 3-fluorobenzaldehyde **1** and ethynylmagnesium chloride **2**, the intermediate **3** was obtained through the Grignard reaction. Then, intermediate **4** was formed by the treatment of thionyl chloride. Intermediate **7a** or **7b** were synthesized from commercially available *tert*-butyl (1*R*,5*S*)-3,8-diazabicyclo[3.2.1]octane-8-carboxylate **5** and substituted nicotinic acid **6a** or **6b** through amide condensation, followed by the deprotection reaction to obtain intermediate **8a** or **8b**. The key fragments C6 and C5 were obtained from a substitution reaction between **4** and **8a** or **8b**.

The intermediate **10** was obtained through amide condensation reaction using 2-(7-diazabenzotriazole)-*N*,*N*,*N*',*N*'-tetramethyluronium hexafluorophosphate (HATU) as the coupling reagent in dichloromethane solvent from commercially available 1-Boc-piperazine **9** and nicotinic acid **6a**. Subsequently, intermediate **11** was obtained by removing the *t*-butyloxyl carbonyl (Boc) protecting group. Finally, intermediate **11** was combined with intermediate 4 through substitution reaction to form the key alkyne fragment C4.

**Scheme 1**. Synthetic Route to the Intermediates and Alkyne fragments C4, C5 and C6

Reagents and conditions : (i) THF, -20°C, N_2_ ; (ii) dichlorosulfoxide, dichloromethane (DCM), ice bath; (iii) 3-(ethyliminomethylideneamino)-*N*,*N*- dimethylpropan-1-amine,hydrochloride (EDCI), 1-Hydroxybenzotriazole (HOBt), *N*-methylmorpholine (NMM), DCM, r.t.; (iv) hydrogen chloride dioxane solution (4M), DCM, r.t.; (v) CH_3_COOK, methanol (MeOH), 70°C; (vi) *O*-(7-azabenzotriazol-1-yl)-*N,N,N',N'*-tetramethyluronium hexafluorophosphate (HATU), *N,N*-diisopropylethylamine (DIPEA), DCM, r.t.

**Scheme 2** illustrates the synthesis the alkyne C3, the intermediate **13** was obtained through amide condensation reaction from commercially available 4-Boc-aminopyridine **12** and nicotinic acid **6a**. Then, followed by the deprotecting reaction to obtain intermediate **14**. Intermediate **15** was synthesized through the Cham-Lam coupling reaction between **14** and 3-fluorophenylboronic acid in the presence of copper acetate as a catalyst. Finally, the key alkyne fragment C3 was formed by substitution reaction between **15** and 3-bromopropyne.

**Scheme 2**. Synthetic Route to the Intermediates and Alkyne fragment C3

Reagents and conditions: (i) HATU, DIPEA, niacin, DCM, r.t.; (ii) hydrogen chloride dioxane solution (4 M), DCM, r.t.; (iii) 3-fluorophenylboronic acid, Cu(OAc)_2_, pyridine, O_2_, DCM,r.t.; (iv) 3-bromopropyne, *t*-BuOK, ethanol , 60 °C.

General Synthesis Procedure A to prepare C5 and C6.

1-(3-fluorophenyl)prop-2-yn-1-ol (3)

Under the nitrogen atmosphere, 3-fluorobenzaldehyde (**1**, 5.0 g, 40 mmol, 1.0 eq.) was added to anhydrous tetrahydrofuran (THF), followed by the gradual addition of 0.5 mol/L ethynylmagnesium bromide/THF solution (**2**, 82.5 mL, 41 mmol, 1.0 eq.). When the reaction was finished monitoring by TLC, then it was quenched with saturated NH_4_Cl solution. Subsequently, the aqueous phase was extracted with EtOAc (30 mL × 3). The resulting organic phase was washed with saturated NaCl solution, dried over anhydrous Na_2_SO_4_, and then concentrated in vacuo successively. The crude product was purified by silica gel column chromatography to afford the pure intermediate **3** (5.2 g, 39.3 mmol, 85.9%) as a light yellow oil. ESI-MS: m/z 133.1 [M+H]^+^. C_9_H_8_O (132.1).

1-(1-chloroprop-2-yn-1-yl)-3-fluorobenzene (4)

The intermediate **3** (2.0 g, 13 mmol, 1.0 eq.) and catalytic amounts of dimethylformamide (DMF) was dissolved in DCM and then a solution of sulfonyl chloride (2.38 g, 20 mmol, 1.5 eq.) in DCM was added slowly under ice bath conditions. The reaction mixture was stirred for 3 hours. After quenching the reaction with saturated NaHCO_3_ solution, the water phase was extracted with DCM (10 mL × 3), and the combined organic layers were dried over Na_2_SO_4_ and concentrated in vacuo to give intermediate **4** (1.7 g, 10.1 mmol, 76%) as a yellow-brown oily liquid. ESI-MS : m/z 169.01 [M+H]^+^. C_9_H_6_ClF (168.01).

*tert*-butyl (1*R*,5*S*)-3-nicotinoyl-3,8-diazabicyclo[3.2.1]octane-8-carboxylate (7a)

*tert*-butyl-(1*R*,5*S*)-3-(5-methylnicotinoyl)-3,8-diazabicyclo[3.2.1]octane-8 carboxylate (7b)

To a mixture of **6a** (2.13 g, 15.5 mmol, 1.1 eq.), EDCI (2.0 g, 10.4 mmol, 1.5 eq.), HOBt (0.95 g, 7.0 mmol, 0.5 eq.) and NMM (4.3 g, 42.5 mmol, 3.0 eq.) and DCM (50 mL) was stirred under ice bath for 15 minutes. Then, commercially available raw material *tert*-butyl-3,8-diazabicyclo[3.2.1]octane-8-carboxylate (**5**, 3.0 g, 14 mmol,1.0 eq.) was added to the system and stirred for 20 hours at room temperature. Upon completion of the reaction, the reaction mixture was washed with water and brine successively, dried over anhydrous Na_2_SO_4_, and then concentrated under reduced pressure. The crude product was purified by on silica gel column chromatography to afford pure **7a** (3.6 g, 11.3 mmol, 76.9%) as a yellow solid. ESI-MS: m/z 318.2 [M+H]^+^. C_17_H_23_N_3_O_3_ (317.2). Compound **6b** underwent similar experimental procedures yielding intermediate compound **7b** (3.8 g, 11.4 mmol, 78.9%) as a yellow solid. ESI-MS: m/z 332.2 [M + H]^＋^.C_18_H_25_N_3_O_3_ (331.2).

((1*R*,5*S*)-3,8-diazabicyclo[3.2.1]octan-3-yl)(pyridin-3-yl)methanone (8a)

((1*R*,5*S*)-3,8-diazabicyclo[3.2.1]octan-3-yl)(5-methylpyridin-3-yl)methanone (8b)

A solution of the intermediate **7a** (2.0 g, 6 mmol, 1.0 eq.) in DCM was slowly added with 4 mol/L HCl/dioxane (10.5 mL, 42 mmol, 6-7 eq.). After 6 hours, the reaction mixture was concentrated under reduced pressure to obtain a white solid. The solid was then dissolved in water. Then, the solution was adjusted pH to 8 - 9 with saturated NaHCO_3_ and was further concentrated in vacuo to yield a white solid. Subsequently, the resulting solid product was stirred in THF, filtered, and concentrated under reduced pressure and dried to afford intermediate **8a** (1.8 g, 8.3 mmol, 89%) as a light-yellow solid. ESI-MS: m/z 218.1 [M+H]^+^. C_12_H_15_N_3_O (217.1).

Compound **7b** underwent similar experimental procedures yielding intermediate compound **8b** (1.3 g, 5.6 mmol, 92%) as a yellow solid. ESI-MS: m/z 232.1 [M+H]^＋^. C_13_H_17_N_3_O (231.1)

((1*R*,5*S*)-8-(1-(3-fluorophenyl)prop-2-yn-1-yl)-3,8-diazabicyclo[3.2.1]octan-3-yl)(5-methylpyridin-3-yl)methanone (C5)

((1*R*,5*S*)-8-(1-(3-fluorophenyl)prop-2-yn-1-yl)-3,8-diazabicyclo[3.2.1]octan-3-yl)(pyridin-3-yl)methanone (C6)

Intermediate **8b** (1.5 g, 6.5 mmol, 1.1 eq.) was dissolved in MeOH, followed by the addition of CH_3_COOK (1.2 g, 12 mmol, 2.0 eq.), and the mixture was stirred for 15 minutes. Intermediate **4** (1.0 g, 6 mmol, 1.0 eq.) was then added to the reaction mixture, which was allowed to warm up to 70°C. Upon completion of the reaction, the reaction mixture followed by adding with DCM (10 mL) and water (10 mL). The organic phase was extracted with DCM (10 mL × 3), then washed with saturated NaCl solution. The combined organic phases were dried over anhydrous Na_2_SO_4_, then filtered and concentrated in vacuo to obtain crude product, which was purified by silica gel column chromatography to afford pure intermediate **C5** (0.7 g, 1.9 mmol, 40.5%) as a yellow solid. ESI-MS: m/z 364.2 [M+H]^＋^, C_22_H_22_FN_3_O (363.2).

The same operation for **8a** to obtain intermediate **C6** (0.75 g, 1.9 mmol, 48.8%) as a yellow solid. ESI-MS: m/z 350.2 [M+H]^+^. C_21_H_20_FN_3_O (349.2).

General procedure for the target compounds in series I (C5N5 to C5N64, C6N17 to C6N21).

Sequentially add intermediate **C5** (0.15 g, 0.41 mmol, 1.0 eq) and the selected azide derivatives (0.08 g, 0.5 mmol, 1.2 eq) into a mixture of THF/H_2_O (15 mL, v/v = 1:1). Then CuSO_4_**·**5H_2_O (0.01g, 0.05 mmol, 0.1 eq) and sodium ascorbate (0.041 g, 0.2 mmol, 0.5 eq) was added. The reaction proceed at 55°C for 24 hours. Upon completion the reaction mixture was concentrated under reduced pressure, and extracted with EtOAc (10 mL × 3). The organic layer was collected and washed with brine (10 mL × 3), then dried over anhydrous Na_2_SO_4_, filtered, and concentrated in vacuo. Purification was conducted on a Biotage Isolera system to yield the target compounds **C5N5** ~ **C5N64**. The same procedure for intermediate **C6** to afford the target compounds **C6N17** ~ **C6N21**.

((1*R*,5*S*)-8-(1-(3-fluorophenyl)prop-2-yn-1-yl)-3,8-diazabicyclo[3.2.1]octan-3-yl)(5-methylpyridin-3-yl)methanone (C5)

^1^H NMR (400 MHz, DMSO-*d*_6_): *δ* 8.45 (d, *J* = 11.8 Hz, 1H, ), 8.37 (d, *J* = 8.9 Hz, 1H), 7.62 (d, *J* = 10.3 Hz, 1H), 7.48 – 7.24 (m, 3H), 7.15 (d, *J* = 8.5 Hz, 1H), 4.40 – 4.26 (m, 1H), 3.85 – 3.55 (m, 1H), 3.44 (d, *J* = 13.6 Hz, 2H), 3.31 – 3.08 (m, 2H), 3.08 – 2.76 (m, 2H), 2.32 (d, *J* = 12.0 Hz, 3H), 1.92 (s, 2H), 1.70 – 1.29 (m, 2H). ^13^C NMR (150 MHz, DMSO-*d*_6_): *δ* 168.81, 163.54, 161.93, 151.05, 149.37, 145.33, 145.09, 142.19, 140.92, 135.31, 133.40, 132.03, 130.93, 129.01, 127.96 (2×C), 124.66, 124.00, 115.08, 114.82, 60.23, 57.21, 56.90, 55.37, 52.60, 49.07, 43.90, 25.58, 24.96, 18.19. ESI-MS: m/z 364.2 [M + H]^＋^. C_22_H_22_FN_3_O (363.2)

^^

((1*R*,5*S*)-8-(bis(3-fluorophenyl)methyl)-3,8-diazabicyclo[3.2.1]octan-3-yl)(5-methylpyridin-3-yl)methanone (AA-625)

Off-white solid, 35% yield. mp 105 -106 ℃. ^1^H NMR (600 MHz, DMSO-*d*_6_): *δ* 8.45 (s, 1H), 8.38 (s, 1H), 7.63 (s, 1H), 7.44 – 7.30 (m, 6H), 7.09 – 6.97 (m, 2H), 4.65 (s, 1H), 4.21 (d, *J* = 12.5 Hz, 1H), 3.51 (d, *J* = 12.0 Hz, 1H), 3.19 (d, *J* = 12.5 Hz, 1H), 3.09 (s, 1H), 3.05 (d, *J* = 12.8 Hz, 1H), 2.94 (s, 1H), 2.32 (s, 3H), 1.99 – 1.89 (m, 2H), 1.58 (d, *J* = 12.4 Hz, 1H), 1.42 (d, *J* = 9.2 Hz, 1H). ^13^C NMR (150 MHz, DMSO-*d*_6_) *δ* 168.83, 163.55, 161.94, 151.08 (2×C), 146.44, 145.15 (2×C), 135.36 (2×C), 133.43, 132.04, 131.14, 123.99 (2×C), 114.54, 114.41 (2×C), 67.05, 57.09 (2×C), 54.12, 48.31, 25.20 (2×C), 18.19. ESI-MS: m/z 434.2 [M + H]^＋^. C_26_H_25_F_2_N_3_O (433.2)

^^

((1*R*,5*S*)-8-((3-fluorophenyl)(1-(3-nitrobenzyl)-1H-1,2,3-triazol-4-yl)methyl)-3,8-diazabicyclo[3.2.1]octan-3-yl)(5-methylpyridin-3-yl)methanone (C5N5)

Orange solid, 80% yield. mp 95 - 96 ℃. ^1^H NMR (400 MHz, DMSO-*d*_6_): *δ* 8.45 (s, 1H), 8.36 (s, 1H), 8.26 (s, 1H), 8.18 (d, *J* = 7.9 Hz, 1H), 8.07 (s, 1H), 7.78 – 7.63 (m, 2H), 7.61 (s, 1H), 7.38 (dd, *J* = 17.6, 9.8 Hz, 3H), 7.07 (s, 1H), 5.76 (s, 1H), 5.72 (d, *J* = 11.3 Hz, 2H), 4.18 (t, *J* = 12.7 Hz, 1H), 3.42 (t, *J* = 12.5 Hz, 1H), 3.23 – 3.06 (m, 2H), 3.06 – 2.91 (m, 2H), 2.31 (s, 3H, pyridine-CH_3_), 1.96 (m, 2H, CH_2_), 1.57 (d, *J* = 11.0 Hz, 1H), 1.41 (d, *J* = 15.2 Hz, 1H). ^13^C NMR (100 MHz, DMSO-*d_6_*) δ 168.78, 163.95, 161.52, 151.06, 148.33, 145.12, 138.68, 135.32, 134.97, 130.87, 124.66, 123.51, 122.89, 114.88, 114.65, 60.25, 57.21, 56.93, 55.39, 52.25, 25.00, 18.19. HRMS (ESI) m/z [M+H]^+^ calcd for C_29_H_28_FN_7_O_3_ 541.5874; found 542.231. HPLC purity: 96.25%.

^^

((1*R*,5*S*)-8-((3-fluorophenyl)(1-(4-nitrobenzyl)-1H-1,2,3-triazol-4-yl)methyl)-3,8-diazabicyclo[3.2.1]octan-3-yl)(5-methylpyridin-3-yl)methanone (C5N6)

Orange solid, 82% yield. mp 96.5 - 98 ℃. ^1^H NMR (600 MHz, DMSO-*d*_6_): *δ* 8.45 (s, 1H), 8.37 (s, 1H), 8.29 – 8.13 (m, 3H), 7.62 (s, 1H), 7.48 (q, *J* = 8.3 Hz, 2H), 7.43 – 7.30 (m, 3H), 7.07 (s, 1H), 5.73 (d, *J* = 16.7 Hz, 2H), 4.80 (s, 1H), 4.25 – 4.13 (m, 1H), 3.43 (t, *J* = 12.1 Hz, 1H), 3.18 (d, *J* = 5.2 Hz, 1H), 3.15 (d, *J* = 20.2 Hz, 1H), 2.99 (q, *J* = 17.3 Hz, 2H), 2.31 (s, 3H), 2.05 – 1.89 (m, 2H), 1.66 – 1.37 (m, 2H). ^13^C NMR (150 MHz, DMSO-*d_6_*) *δ* 168.81, 163.54, 161.93, 151.04, 149.41, 147.67, 145.27, 145.09, 143.91, 135.30, 133.43, 132.03, 130.94, 129.34, 127.71, 124.66, 124.38 (2×C), 124.03, 114.82, 60.24, 57.21, 56.91, 53.90, 52.42, 48.20, 47.78, 25.57, 24.96, 18.18. HRMS (ESI) m/z [M+H]^+^ calcd for C_29_H_28_FN_7_O_3_ 541.5874; found 542.231. HPLC purity: 97.35%.

((1*R*,5*S*)-8-((3-fluorophenyl)(1-phenethyl-1H-1,2,3-triazol-4-yl)methyl)-3,8-diazabicyclo[3.2.1]octan-3-yl)(5-methylpyridin-3-yl)methanone(C5N16)

Off-white solid, 85% yield. mp 90 - 93 ℃. ^1^H NMR (400 MHz, DMSO-*d*_6_): *δ* 8.46 (d, *J* = 13.7 Hz, 1H), 8.37 (d, *J* = 6.4 Hz, 1H), 7.82 (s, 1H), 7.62 (d, *J* = 8.8 Hz, 1H), 7.40 – 7.26 (m, 3H), 7.24 – 7.09 (m, 3H), 7.07 (s, 3H), 4.68 (d, *J* = 6.5 Hz, 1H), 4.56 (d, *J* = 6.7 Hz, 2H), 4.15 (t, *J* = 11.6 Hz, 1H), 3.19 – 3.01 (m, 3H), 2.92 (t, *J* = 13.5 Hz, 2H), 2.83 – 2.71 (m, 1H), 2.32 (d, *J* = 11.7 Hz, 3H), 1.99 – 1.83 (m, 2H), 1.56 – 1.34 (m, 2H), 1.23 (s, 1H). ^13^C NMR (150 MHz, DMSO-*d*_6_): δ 168.79, 163.55, 161.93, 151.10, 148.86, 145.14, 138.07, 135.34, 133.43, 132.09, 130.85, 129.19, 128.69, 126.94, 126.80, 124.62, 123.46, 123.36, 60.35, 56.75, 55.38, 54.08, 51.03, 48.29, 36.09, 25.31, 24.96, 18.20. HRMS (ESI) m/z [M+H]^+^ calcd for C_30_H_31_FN_6_O 510.6174; found 511.2616. HPLC purity: 95.37%.

((1*R*,5*S*)-8-((3-fluorophenyl)(1-(2-(thiophen-3-yl)ethyl)-1H-1,2,3-triazol-4-yl)methyl)-3,8-diazabicyclo[3.2.1]octan-3-yl)(5-methylpyridin-3-yl)methanone(C5N17)

Orange solid, 70% yield. mp 89.5 - 91℃. ^1^H NMR (400 MHz, DMSO-*d*_6_): *δ* 8.61 – 8.24 (m, 2H), 7.85 (s, 1H), 7.62 (s, 1H), 7.45 – 7.26 (m, 4H), 7.07 (s, 2H), 6.90 (d, *J* = 4.9 Hz, 1H), 4.70 (d, *J* = 8.3 Hz, 1H), 4.55 (d, *J* = 7.2 Hz, 2H), 4.17 (t, *J* = 12.5 Hz, 1H), 3.13 (d, *J* = 11.0 Hz, 3H), 3.03 – 2.77 (m, 3H), 2.32 (d, *J* = 7.1 Hz, 3H), 2.00 – 1.85 (m, 2H), 1.55 (d, *J* = 7.3 Hz, 1H), 1.46 – 1.33 (m, 1H), 1.23 (s, 1H). ^13^C NMR (150 MHz, DMSO-*d*_6_): δ168.78, 163.54, 161.93, 151.08, 148.69, 145.39, 145.14, 138.27, 135.35, 133.43, 132.04, 130.87, 128.63, 126.51, 124.62, 123.42, 122.61, 114.95, 60.37, 57.05, 56.79, 53.99, 50.35, 48.11, 30.75, 25.34, 24.97, 18.20. HRMS (ESI) m/z [M+H]^+^ calcd for C_28_H_29_FN_6_OS 516.6394; found 517.218. HPLC purity: 95.97%.

2-(2-(4-((3-fluorophenyl)((1*R*,5*S*)-3-(5-methylnicotinoyl)-3,8-diazabicyclo[3.2.1] octan-8-yl)methyl)-1H-1,2,3-triazol-1-yl)ethyl)isoindoline-1,3-dione (C5N21)

Off-white solid, 80% yield. mp 119 - 120℃. ^1^H NMR (400 MHz, DMSO-*d*_6_): *δ* 8.58 – 8.43 (m, 1H), 8.37 (s, 1H), 8.12 (s, 1H), 7.89 – 7.57 (m, 5H), 7.33 (s, 1H), 7.31 – 7.25 (m, 2H), 7.04 (d, *J* = 8.5 Hz, 1H), 5.76 (s, 1H), 4.69 (s, 1H), 4.59 (s, 2H), 4.14 (t, *J* = 12.1 Hz, 1H), 3.99 (s, 2H), 3.18 – 2.77 (m, 4H), 2.42 – 2.26 (m, 3H), 1.90 (s, 2H), 1.55 (d, *J* = 9.3 Hz, 1H), 1.39 (d, *J* = 10.2 Hz, 1H). ^13^C NMR (150 MHz, DMSO) *δ* 168.82, 167.76, 163.50, 161.89, 151.12, 149.15, 145.34, 145.10, 144.95, 135.30, 135.01, 134.87, 132.07, 131.80, 130.76, 130.75, 124.62, 123.75, 123.46, 123.37, 114.95, 114.66, 60.26, 57.05, 56.69, 55.36, 53.90, 48.11, 38.45, 25.44, 24.99, 18.23. HRMS (ESI) m/z [M+H]^+^ calcd for C_32_H_30_FN_7_O_3_ 579.6364; found 580.2467. HPLC purity: 96.76%.

Methyl4-(4-((3-fluorophenyl)((1*R*,5*S*)-3-(5-methylnicotinoyl)-3,8-diazabicyclo [3.2.1]octan-8-yl)methyl)-1H-1,2,3-triazol-1-yl)butanoate (C5N39)

Orange solid, 83% yield. mp 75.5 - 78 ℃. ^1^H NMR (400 MHz, DMSO-*d*_6_) *δ* 8.44 (s, 1H), 8.36 (d, *J* = 2.1 Hz, 1H), 8.08 (s, 1H), 7.61 (s, 1H), 7.46 – 7.29 (m, 3H), 7.07 (s, 1H), 4.75 (s, 1H), 4.39 – 4.26 (m, 2H), 4.17 (t, *J* = 11.1 Hz, 1H), 3.54 (d, *J* = 8.3 Hz, 3H), 3.48 – 3.37 (m, 1H), 3.14 (d, *J* = 13.8 Hz, 1H), 2.99 (d, *J* = 17.9 Hz, 2H), 2.88 (d, *J* = 6.6 Hz, 1H), 2.29 (d, *J* = 14.5 Hz, 5H), 2.09 – 1.90 (m, 4H), 1.57 (d, *J* = 8.1 Hz, 1H), 1.41 (d, *J* = 12.5 Hz, 1H). ^13^C NMR (150 MHz, DMSO) *δ* 172.95, 168.79, 163.54, 161.93, 151.04, 148.97, 145.46, 145.14, 133.39, 132.09, 130.87, 124.68, 123.42, 115.01, 114.71, 60.37, 57.21, 56.83, 54.03, 53.91, 51.82, 49.09, 48.23, 30.62, 25.47, 24.96, 18.19. HRMS (ESI) m/z [M+H]^+^ calcd for C_27_H_31_FN_6_O_3_ 506.5824; found, 507.2514. HPLC purity: 97.15%.

((1*R*,5*S*)-8-((3-fluorophenyl)(1-((phenylsulfinyl)methyl)-1H-1,2,3-triazol-4-yl)methyl)-3,8-diazabicyclo[3.2.1]octan-3-yl)(5-methylpyridin-3-yl)methanone (C5N41)

White solid, 83% yield. mp 118 - 120 ℃. ^1^H NMR (400 MHz, DMSO-*d*_6_): *δ* 8.46 (s, 1H), 8.38 (s, 1H), 7.75 – 7.65 (m, 1H), 7.63 (d, *J* = 5.1 Hz, 1H), 7.51 (s, 1H), 7.47 – 7.35 (m, 5H), 7.31 (d, *J* = 9.9 Hz, 2H), 7.09 (d, *J* = 7.2 Hz, 1H), 5.84 (d, *J* = 12.6 Hz, 1H), 5.75 – 5.53 (m, 1H), 4.72 (d, *J* = 13.4 Hz, 1H), 4.28 – 4.09 (m, 1H), 3.39 (d, *J* = 11.4 Hz, 1H), 3.15 (d, *J* = 10.9 Hz, 1H), 3.06 – 2.90 (m, 2H), 2.85 (s, 1H), 2.32 (s, 3H), 1.93 (s, 2H), 1.57 (d, *J* = 11.9 Hz, 1H), 1.41 (d, *J* = 15.3 Hz, 1H). ^13^C NMR (100 MHz, DMSO-*d*_6_): *δ* 168.82, 163.94, 161.52, 151.09, 148.72, 145.14, 140.34, 140.32, 135.35, 133.45, 132.05, 130.96, 129.54, 129.49, 127.85, 124.88, 124.85, 124.60, 114.86 (2×C), 68.54, 59.92, 56.84, 56.09, 53.77, 48.00, 25.36, 25.01, 18.20. HRMS (ESI) m/z [M+H]^+^ calcd for C_29_H_29_FN_6_O_2_S 544.6494; found, 545.2129. HPLC purity: 97.26%.

((1*R*,5*S*)-8-((3-fluorophenyl)(1-((phenylsulfonyl)methyl)-1H-1,2,3-triazol-4-yl)methyl)-3,8-diazabicyclo[3.2.1]octan-3-yl)(5-methylpyridin-3-yl)methanone

(C5N42)

White solid, 85% yield. mp 111 - 112 ℃. ^1^H NMR (400 MHz, DMSO-*d*_6_): *δ* 8.46 (s, 1H), 8.38 (s, 1H), 7.75 – 7.65 (m, 1H), 7.65 – 7.58 (m, 1H), 7.51 (s, 1H), 7.44 (d, *J* = 13.9 Hz, 5H), 7.31 (d, *J* = 9.2 Hz, 2H), 7.09 (d, *J* = 8.8 Hz, 1H), 5.84 (d, *J* = 13.3 Hz, 1H), 5.75 (s, 2H), 4.28 – 4.09 (m, 1H), 3.39 (d, *J* = 11.9 Hz, 1H), 3.15 (d, *J* = 12.4 Hz, 1H), 3.04 – 2.90 (m, 2H), 2.85 (s, 1H), 2.32 (s, 3H), 1.93 (s, 2H), 1.57 (d, *J* = 12.8 Hz, 1H), 1.41 (d, *J* = 15.7 Hz, 1H). ^13^C NMR (100 MHz, DMSO-*d*_6_): *δ*: 168.82, 163.91, 161.52, 151.08, 145.14, 140.32, 135.34, 133.43, 132.03 (2×C), 130.92, 129.54 (2×C), 129.48 (2×C), 124.88, 124.85, 124.58, 114.87 (2×C), 68.53, 59.94, 56.82, 55.38, 54.06, 48.28, 25.02 (2×C), 18.21. HRMS (ESI) m/z [M+H]^+^ calcd for C_29_H_29_FN_6_O_3_S 560.6484; found, 561.2079. HPLC purity: 97.32%.

((1*R*,5*S*)-8-((3-fluorophenyl)(1-(4-(methylsulfonyl)benzyl)-1H-1,2,3-triazol-4-yl)methyl)-3,8-diazabicyclo[3.2.1]octan-3-yl)(5-methylpyridin-3-yl)methanone (C5N50)

White solid, 81% yield. mp 125 - 128 ℃. ^1^H NMR (600 MHz, DMSO-*d*_6_): *δ* 8.45 (s, 1H), 8.37 (s, 1H), 8.22 (d, *J* = 8.0 Hz, 1H), 7.92 (q, *J* = 7.9 Hz, 2H), 7.62 (s, 1H), 7.48 (q, *J* = 7.7 Hz, 2H), 7.39 (dd, *J* = 23.9, 13.3 Hz, 3H), 7.07 (s, 1H), 5.69 (d, *J* = 17.1 Hz, 2H), 4.79 (s, 1H), 4.19 (dd, *J* = 22.6, 11.7 Hz, 1H), 3.43 (t, *J* = 13.1 Hz, 1H), 3.18 (s, 3H), 3.17 – 3.01 (m, 2H), 3.01 – 2.85 (m, 2H), 2.31 (s, 3H), 1.94 (d, *J* = 31.2 Hz, 2H), 1.65 – 1.37 (m, 2H). ^13^C NMR (150 MHz, DMSO-*d*_6_): *δ* 168.81, 163.54, 161.93, 151.05, 149.37, 145.33, 145.09, 142.19, 140.92, 135.31, 133.40, 132.03, 130.93, 129.01, 127.96 (2×C), 124.66, 124.00, 115.08, 114.82, 60.23, 57.21, 56.90, 55.37, 52.60, 49.07, 43.90, 25.58, 24.96, 18.19. HRMS (ESI) m/z [M+H]^+^ calcd for C_30_H_31_FN_6_O_3_S 574.6754; found 575.2235. HPLC purity: 95.86%.

2-(4-((3-fluorophenyl)((1*R*,5*S*)-3-(5-methylnicotinoyl)-3,8-diazabicyclo[3.2.1]octan-8-yl)methyl)-1H-1,2,3-triazol-1-yl)-N-methylacetamide (C5N57)

White solid, 82% yield. mp 106 - 107 ℃. ^1^H NMR (400 MHz, DMSO-*d*_6_): *δ* 8.45 (s, 1H), 8.36 (s, 1H), 8.21 (s, 1H), 8.00 (s, 1H), 7.62 (s, 1H), 7.46 – 7.31 (m, 3H), 7.08 (s, 1H), 5.00 (d, *J* = 10.0 Hz, 2H), 4.77 (d, *J* = 4.0 Hz, 1H), 4.17 (t, *J* = 12.0 Hz, 1H), 3.51 – 3.39 (m, 1H), 3.25 – 3.03 (m, 2H), 3.01 – 2.82 (m, 2H), 2.61 (s, 3H), 2.31 (s, 3H), 2.02 – 1.90 (m, 2H), 1.57 (d, *J* = 9.4 Hz, 1H), 1.41 (d, *J* = 13.8 Hz, 1H). ^13^C NMR (100 MHz, DMSO-*d*_6_): *δ* 168.79, 166.20, 163.94, 161.52, 151.05, 148.46, 145.13, 138.75, 135.32, 133.40, 132.04, 130.90, 124.89, 124.68, 114.80, 60.20, 57.12, 56.78, 55.38, 52.07, 48.05, 26.07, 25.01, 25.01, 18.19. HRMS (ESI) m/z [M+H]^+^ calcd for C_25_H_28_FN_7_O_2_ 477.5444; found 478.2361. HPLC purity: 96.27%.

2-(4-((3-fluorophenyl)((1*R*,5*S*)-3-(5-methylnicotinoyl)-3,8-diazabicyclo[3.2.1]octan-8-yl)methyl)-1H-1,2,3-triazol-1-yl)acetamide (C5N58)

Yellow solid, 83% yield. mp 96.5 - 97 ℃. ^1^H NMR (600 MHz, DMSO-*d*_6_) *δ* 8.45 (s, 1H), 8.37 (s, 1H), 7.99 (d, *J* = 3.8 Hz, 1H), 7.75 – 7.57 (m, 2H), 7.45 – 7.26 (m, 4H), 7.08 (s, 1H), 5.00 (d, *J* = 16.3 Hz, 2H), 4.78 (d, *J* = 11.5 Hz, 1H), 4.18 (t, *J* = 15.4 Hz, 1H), 3.44 (dd, *J* = 19.8, 12.5 Hz, 1H), 3.21 – 2.85 (m, 4H), 2.31 (s, 3H), 1.94 (d, *J* = 33.3 Hz, 2H), 1.67 – 1.36 (m, 2H). ^13^C NMR (150 MHz, DMSO-*d*_6_) *δ* 168.80, 167.70, 163.54, 161.93, 151.04, 148.40, 145.46, 145.11, 135.31, 133.40, 132.05, 130.86, 125.02, 124.69, 114.72, 60.22, 57.24, 56.77, 55.37, 51.92, 48.23, 25.57, 25.01, 18.19. HRMS (ESI) m/z [M+H]^+^ calcd for C_24_H_26_FN_7_O_2_, 463.5174; (M+H)^+^,464.2205. HPLC purity: 95.51%.

((1*R*,5*S*)-8-((1-(3-fluorobenzyl)-1H-1,2,3-triazol-4-yl)(3-fluorophenyl)methyl)-3,8-diazabicyclo[3.2.1]octan-3-yl)(5-methylpyridin-3-yl)methanone (C5N64)

Off-white solid, 85% yield. mp 90 - 93 ℃. ^1^H NMR (600 MHz, DMSO-*d*_6_): *δ* 8.45 (s, 1H), 8.37 (s, 1H), 8.19 (d, *J* = 7.6 Hz, 1H), 7.61 (s, 1H), 7.45 – 7.28 (m, 4H), 7.22 – 7.03 (m, 4H), 5.57 (d, *J* = 17.5 Hz, 2H), 4.78 (d, *J* = 7.1 Hz, 1H), 4.18 (t, *J* = 12.9 Hz, 1H), 3.43 (t, *J* = 13.5 Hz, 1H), 3.18 (d, *J* = 5.2 Hz, 1H), 3.12 (d, *J* = 26.5 Hz, 1H), 3.04 – 2.86 (m, 2H), 2.31 (s, 3H), 2.05 – 1.88 (m, 2H), 1.64 – 1.38 (m, 2H). ^13^C NMR (150 MHz, DMSO-*d*_6_): *δ* 168.80, 163.54, 161.92, 151.04, 149.36, 145.31, 145.08, 139.14, 135.31, 133.43, 132.04, 131.27, 130.91, 124.64, 124.35, 123.84, 115.48, 115.23, 115.09, 114.78, 60.27, 57.21, 56.89, 54.01, 52.63, 48.19, 25.57, 24.97, 18.18. HRMS (ESI) m/z [M+H]^+^ calcd for C_29_H_28_F_2_N_6_O 514.5808; found 515.2365. HPLC purity: 95.04%.

((1*R*,5*S*)-8-((3-fluorophenyl)(1-(2-(thiophen-3-yl)ethyl)-1H-1,2,3-triazol-4-yl)methyl)-3,8-diazabicyclo[3.2.1]octan-3-yl)(pyridin-3-yl)methanone (C6N17)

Orange solid, 85% yield. mp 115 - 116 ℃. ^1^H NMR (400 MHz, DMSO-*d*_6_): *δ* 8.59 (d, *J* = 14.7 Hz, 2H), 7.85 (s, 1H), 7.80 (s, 1H), 7.50 – 7.24 (m, 5H), 7.08 (d, *J* = 9.1 Hz, 2H), 6.90 (s, 1H), 4.71 (d, *J* = 9.9 Hz, 1H), 4.55 (d, *J* = 8.0 Hz, 2H), 4.16 (t, *J* = 12.9 Hz, 1H), 3.32 (s, 1H), 3.13 (d, *J* = 10.3 Hz, 3H), 2.94 (d, *J* = 12.8 Hz, 2H), 2.82 (s, 1H), 1.92 (s, 2H), 1.56 (d, *J* = 8.7 Hz, 1H), 1.39 (d, *J* = 9.9 Hz, 1H). ^13^C NMR (100 MHz, DMSO-*d*_6_): *δ* 168.69, 163.94, 161.51, 150.75, 147.99, 145.47, 138.26, 135.16, 132.55, 130.87, 130.12, 128.62, 126.50, 124.61, 123.99, 123.40, 122.61, 114.71, 60.34, 57.20, 56.76 (2×C), 50.35 (2×C), 30.75, 25.37, 24.99. HRMS (ESI) m/z [M+H]^+^ calcd for C_27_H_27_FN_6_OS 502.6124; found, 503.2024. HPLC purity: 98.82 %.

2-(2-(4-((3-fluorophenyl)((1*R*,5*S*)-3-nicotinoyl-3,8-diazabicyclo[3.2.1]octan-8-yl)methyl)-1H-1,2,3-triazol-1-yl)ethyl)isoindoline-1,3-dione (C6N21)

White solid, 81% yield. mp 115 - 116 ℃. ^1^H NMR (400 MHz, DMSO-*d*_6_): *δ* 8.72 – 8.59 (m, 1H), 8.58 (s, 1H), 8.11 (s, 1H), 7.83 (s, 1H), 7.80 (d, *J* = 5.0 Hz, 2H), 7.73 (s, 1H), 7.67 (s, 1H), 7.56 – 7.41 (m, 1H), 7.32 (s, 1H), 7.29 (d, *J* = 8.0 Hz, 2H), 7.04 (d, *J* = 8.7 Hz, 1H), 4.68 (s, 1H), 4.59 (s, 2H), 4.12 (d, *J* = 11.8 Hz, 1H), 3.98 (s, 2H), 3.29 (s, 1H), 3.14 – 2.74 (m, 4H), 1.90 (s, 2H), 1.54 (s, 1H), 1.39 (s, 1H). ^13^C NMR (150 MHz, DMSO) *δ* 167.83, 167.06, 163.47, 161.85, 150.90, 148.07, 145.90, 143.52, 135.26, 134.98, 132.18, 131.84, 130.61, 124.71, 124.03, 123.51, 115.27, 114.57, 64.99, 50.57, 50.05, 48.16, 47.80, 42.15, 38.49. HRMS (ESI) m/z [M+H]^+^ calcd for C_31_H_28_FN_7_O_3_ 565.6094; found 566.231. HPLC purity: 99.34 %.

General Synthesis Procedure of C4.

*tert*-butyl 4-nicotinoylpiperazine-1-carboxylate(10)

To a suspension of raw material nicotinic acid **6a** (1.8 g, 14.6 mmol, 1.1 eq.) in 15 mL DCM, HATU (7.6 g, 20 mmol, 1.5 eq.) was added under ice bath. The mixture was stirred for 15 minutes and then added with DIPEA (5.1 g , 39.5 mmol, 3.0 eq.) and commercially available raw material **9** (2.47 g, 13.3 mmol, 1.0 eq.). The solution turned light yellow. When the reaction was finished monitoring by TLC, the mixture was washed with water (10 mL) and saturated NaCl solution successively. The organic phase was dried over anhydrous Na_2_SO_4_, filtered, and concentrated. And the resulting crude product was isolated and purified by silica gel column chromatography to give intermediate **10** (3.1 g, 10.6 mmol, 80%) as a yellow solid. ESI-MS: m/z 292.2 [M + H]^+^. C_15_H_21_N_3_O_3_ (291.2).

piperazin-1-yl(pyridin-3-yl)methanone(11)

To a solution of the intermediate **10** (2.0 g, 6.9 mmol, 1.0 eq) in DCM was slowly added a solution of HCl/dioxane (4 mol/L) (4.8 mL, 6.8 mmol, 6-7 eq.) dropwise. After about 6 hours, the reaction mixture was concentrated under reduced pressure to obtain a white solid. The solid was then dissolved in water. Then the solution was treated dropwise with saturated NaHCO_3_ to pH 8-9 and then concentrated in vacuo to yield a white solid product. Subsequently, the resulting solid product was then stirred in THF, filtered, washed, concentrated in vacuo and dried to afford intermediate **11**. (1.15 g, 6 mmol, 87.8%) as a yellow solid. ESI-MS: m/z 192.1 [M+H]^＋^. C_10_H_13_N_3_O (191.1)

(4-(1-(3-fluorophenyl)prop-2-yn-1-yl)piperazin-1-yl)(pyridin-3-yl)methanone(12)

Intermediate **11** (1.0 g, 5.2 mmol, 1.1 eq.) was dissolved in MeOH and subsequently CH_3_COOK (1.2 g, 10.2 mmol, 2.0 eq.) was added with stirring for 15 minutes. Further, Intermediate **4** (0.8 g, 4.8 mmol, 1.0 eq.) was added in the reaction mixture, which was heated to 70°C. After the reaction finished, the reaction mixture followed by adding with DCM (10 mL) and water (10 mL). The organic phase was extracted with DCM (10 mL × 3), then washed three times with brine. The combined organic phases were dried over anhydrous Na_2_SO_4_, then filtered and concentrated in vacuo to obtain crude product, which was purified by silica gel column chromatography to afford pure intermediate **C4** (0.56 g, 1.7 mmol, 33.1%) as a yellow solid. ESI-MS: m/z 324.1 [M+H]^＋^.C_22_H_22_FN_3_O (323.1).

General procedure for the compounds in series II (C4N17 and C4N21).

Sequentially add intermediate **C4** (0.15 g, 0.41 mmol, 1.0 eq) and the selected diazonium derivative (0.075 g, 0.5 mmol, 1.2 eq), followed by dissolution in a mixture of THF/H_2_O (15 mL, v/v = 1:1). Then introduce CuSO_4_**·**5H_2_O (0.01g, 0.05 mmol, 0.1 eq) and sodium ascorbate (0.041 g, 0.2 mmol, 0.5 eq), and allow the reaction to proceed at 55°C for 24 hours. Monitor the progress of the reaction using TLC. Concentrate the reaction mixture under reduced pressure, extract it with EtOAc (10 mL × 3), collect the organic phase, wash it with saturated NaCl solution. The organic layer was dried over anhydrous Na_2_SO_4_, filtered, and concentrated in vacuo. Purification was conducted on a Biotage Isolera One flash chromatography to afford the target compounds **C4N17** ~ **C4N21**.

(4-((3-fluorophenyl)(1-(2-(thiophen-3-yl)ethyl)-1H-1,2,3-triazol-4-yl)methyl)piperazin-1-yl)(pyridin-3-yl)methanone (C4N17)

Orange solid, 82% yield. mp 163 - 165 ℃. ^1^H NMR (400 MHz, DMSO-*d*_6_): *δ* 8.78 – 8.42 (m, 2H), 7.87 (s, 1H), 7.79 (d, *J* = 7.8 Hz, 1H), 7.49 – 7.43 (m, 1H), 7.40 (dd, *J* = 4.9, 3.0 Hz, 1H), 7.38 – 7.31 (m, 1H), 7.19 (dd, *J* = 12.7, 8.9 Hz, 2H), 7.09 (dd, *J* = 8.5, 2.7 Hz, 2H), 6.92 (d, *J* = 5.0 Hz, 1H), 4.78 (s, 1H), 4.59 (t, *J* = 7.0 Hz, 2H), 3.62 (s, 4H), 3.16 (t, *J* = 7.0 Hz, 2H), 2.30 (s, 4H). ^13^C NMR (100 MHz, DMSO-*d*_6_): *δ* 167.05, 163.89, 161.47, 150.90, 148.09, 146.01, 143.75, 138.25, 135.28, 132.15, 130.76, 130.68, 128.66, 126.51, 124.77, 124.12, 122.64, 115.27, 114.68, 70.39, 65.52, 50.35 (2×C), 47.69, 30.74. HRMS (ESI) m/z [M+H]^+^ calcd for C_25_H_25_FN_6_OS 476.5744; found 477.1867. HPLC purity: 95.51%.

2-(2-(4-((3-fluorophenyl)(4-nicotinoylpiperazin-1-yl)methyl)-1H-1,2,3-triazol-1-yl)ethyl)isoindoline-1,3-dione (C4N21)

White solid, 84% yield. mp 113.5 - 115 ℃. ^1^H NMR (400 MHz, DMSO-*d*_6_): *δ* 8.65 (d, *J* = 4.8 Hz, 1H), 8.59 (s, 1H), 8.13 (s, 1H), 7.85 – 7.73 (m, 5H), 7.47 (dd, *J* = 7.8, 4.9 Hz, 1H), 7.34 (td, *J* = 8.0, 6.1 Hz, 1H), 7.22 – 7.13 (m, 2H), 7.11 – 7.03 (m, 1H), 4.84 (s, 1H), 4.63 (t, *J* = 5.4 Hz, 2H), 4.06 – 3.96 (m, 2H), 3.62 (s, 2H), 3.27 (s, 2H), 2.38 – 2.20 (m, 4H). ^13^C NMR (150 MHz, DMSO-*d*_6_) *δ* 167.83 (2×C), 167.06, 163.47, 161.85, 150.90, 148.07, 145.90, 143.52, 135.26, 134.98 (2×C), 132.18, 131.84 (2×C), 130.61, 124.71, 124.03, 123.51 (2×C), 115.27, 114.57, 64.99, 50.57, 50.05, 48.16, 47.80, 42.15, 38.49. HRMS (ESI) m/z [M+H]^+^ calcd for C_29_H_26_FN_7_O_3_ 539.5714; found 540.2154. HPLC purity: 96.66%.

3.3 Chiral separation of the target compound **C5N17**

The enantiomer C5N17 was separated in chiral HPLC system Shimadzu LC-20AT. For this purpose, the CHIRALPAK AD-H (ADH0CE-CW056) chiral column with dimensions of 0.46 cm I.D. × 25 cm L was selected. The injection volume was set at 10 μL, and a gradient elution with a mobile phase consisting of n-hexane:ethanol in a ratio of 20:80 (v/v) was utilized. The flow rate was maintained at 1.0 mL/min, and the temperature controlled at 35°C. Component C5N17A was collected when its retention time reached 6.792 min, while component C5N17B was collected at a retention time of 8.041 min. Optically pure isomers were obtained through solvent removal via rotary evaporation.

3.4 Experimental procedures and characterization data

3.4.1 NMR spectra of final compounds

^1^H NMR (400 MHz, DMSO-*d*_6_) of **C5**


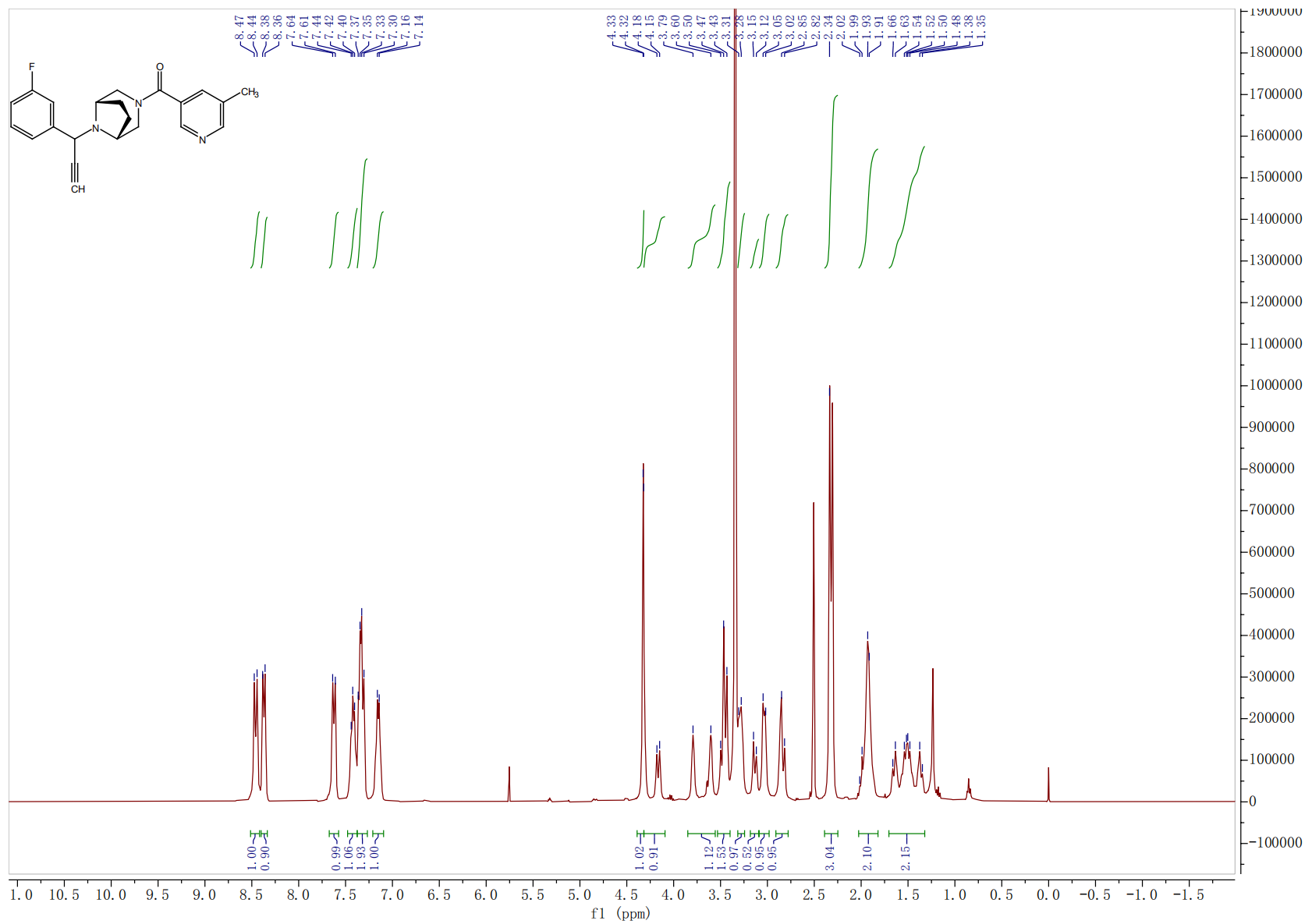


^1^H NMR (100 MHz, DMSO-*d*_6_) of **C5**


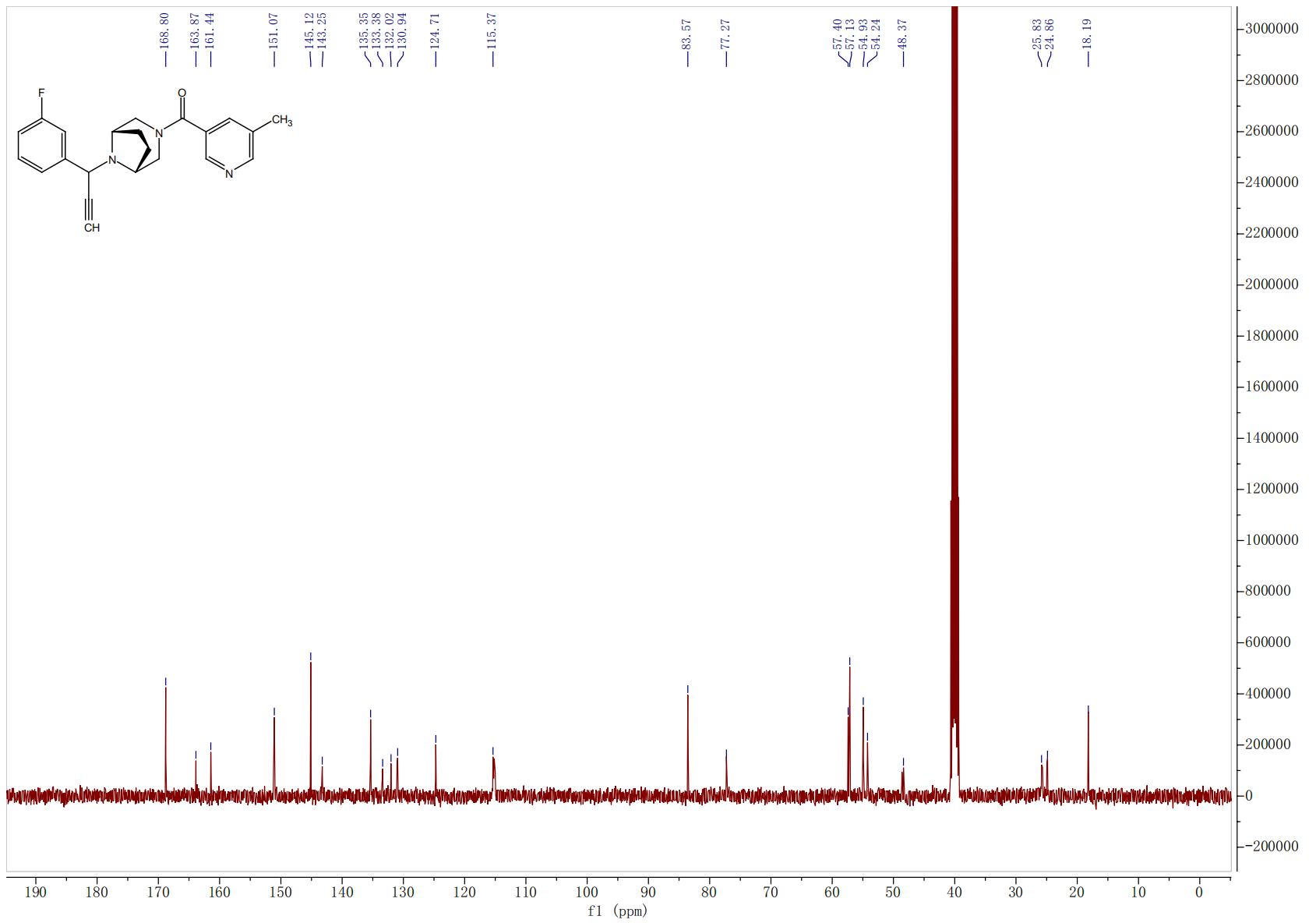


^1^H NMR (600 MHz, DMSO-*d*_6_) of **AA-625**


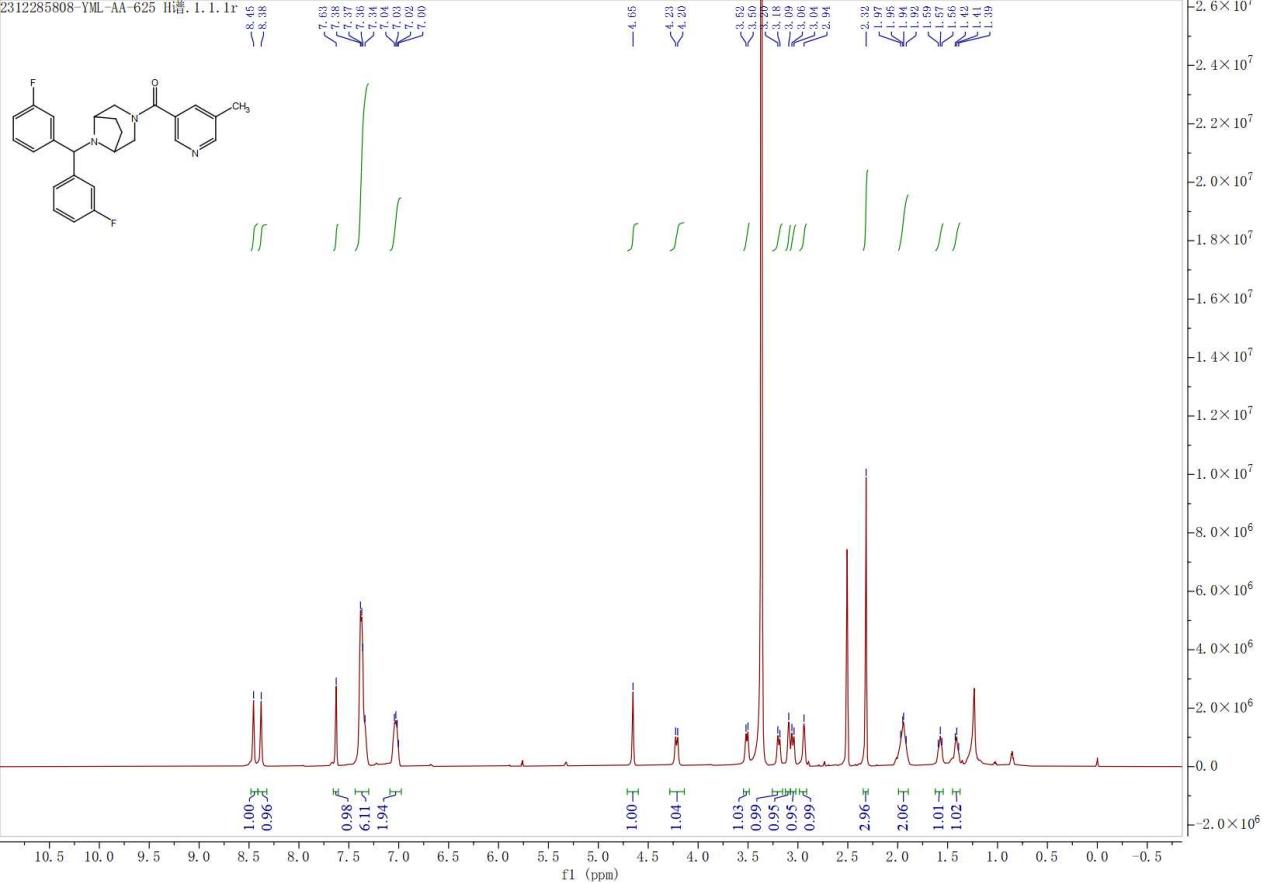


^13^C NMR (150 MHz, DMSO-*d*_6_) of **AA-625**


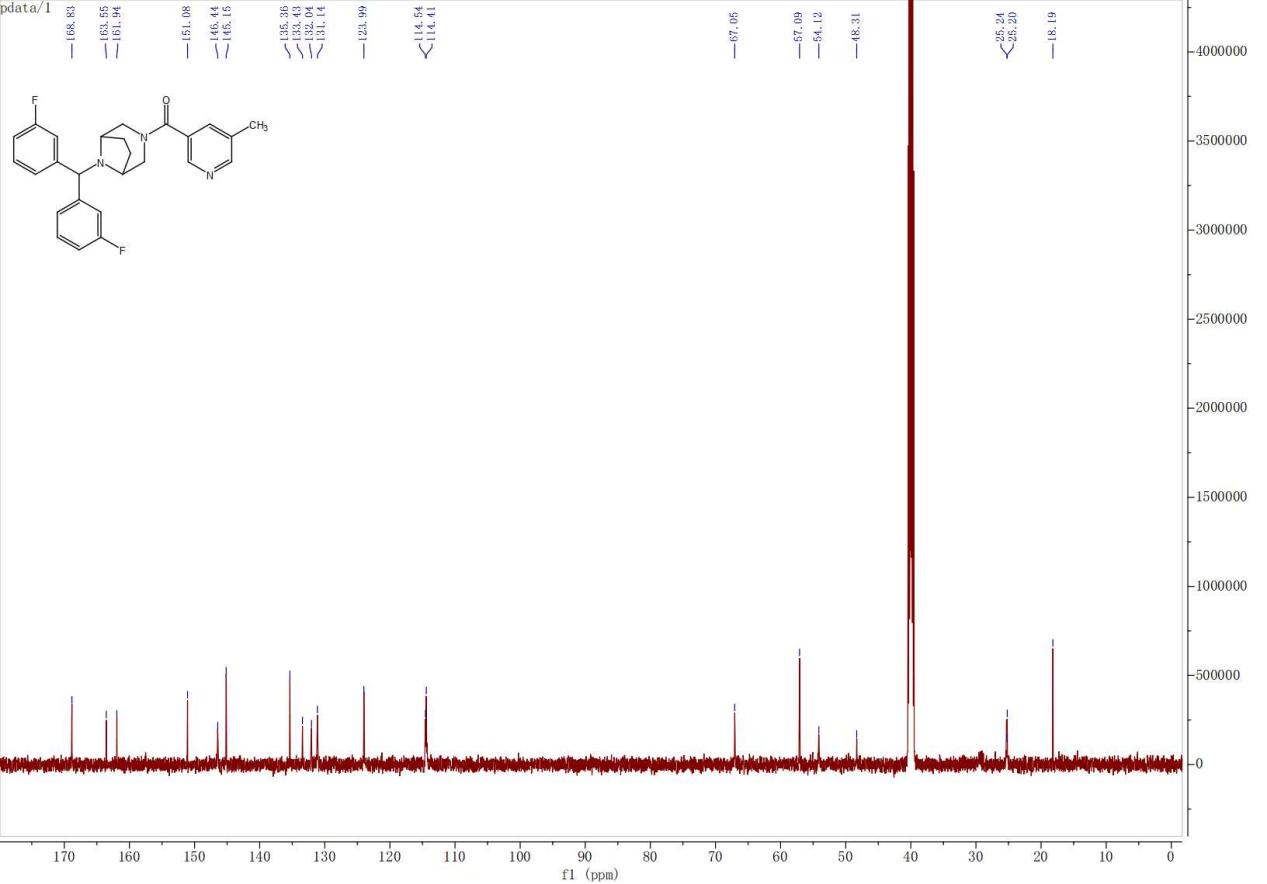


^1^H NMR (400 MHz, DMSO-*d*_6_) of **C5N5**


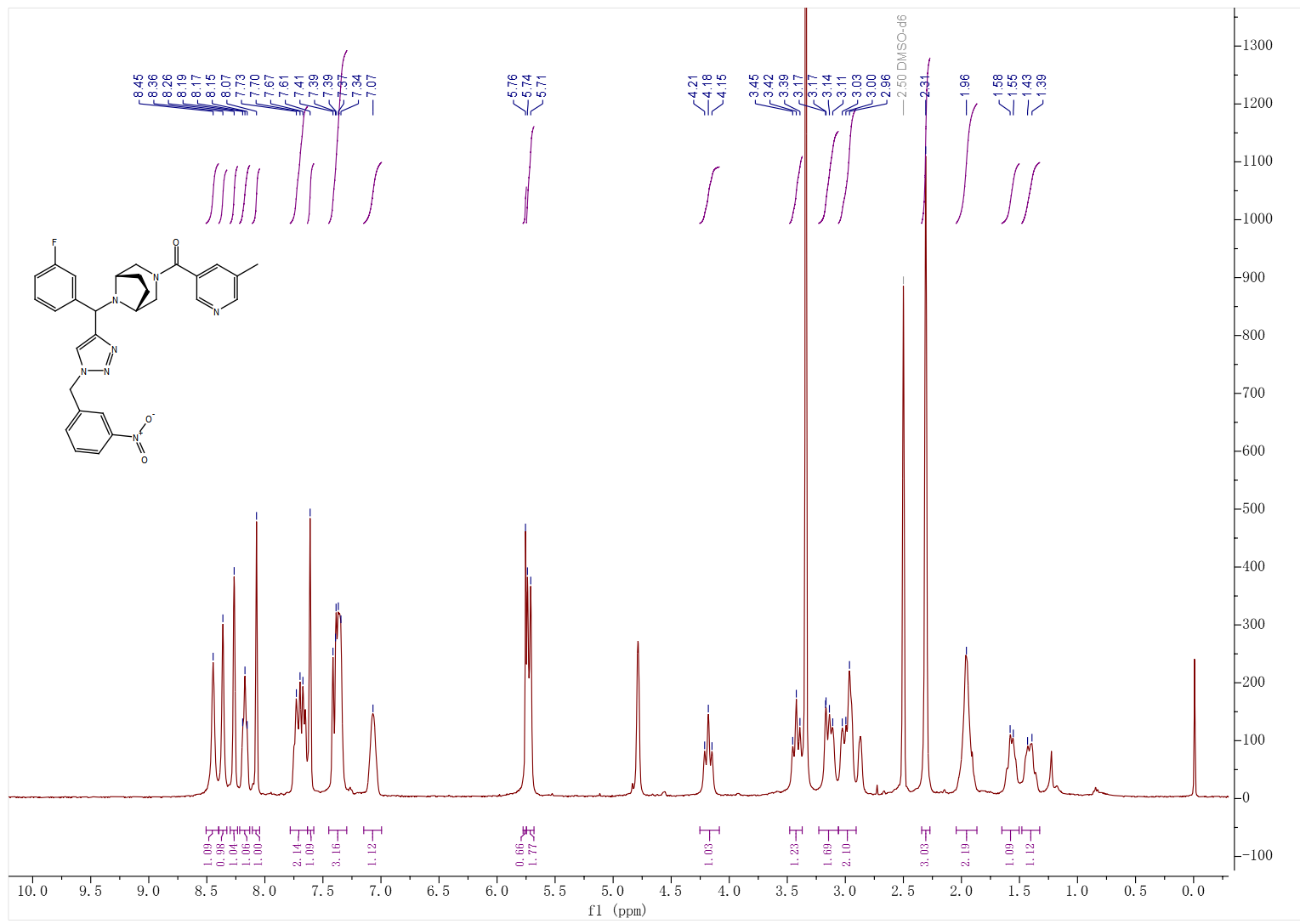


^13^C NMR (100 MHz, DMSO-*d*_6_) of **C5N5**


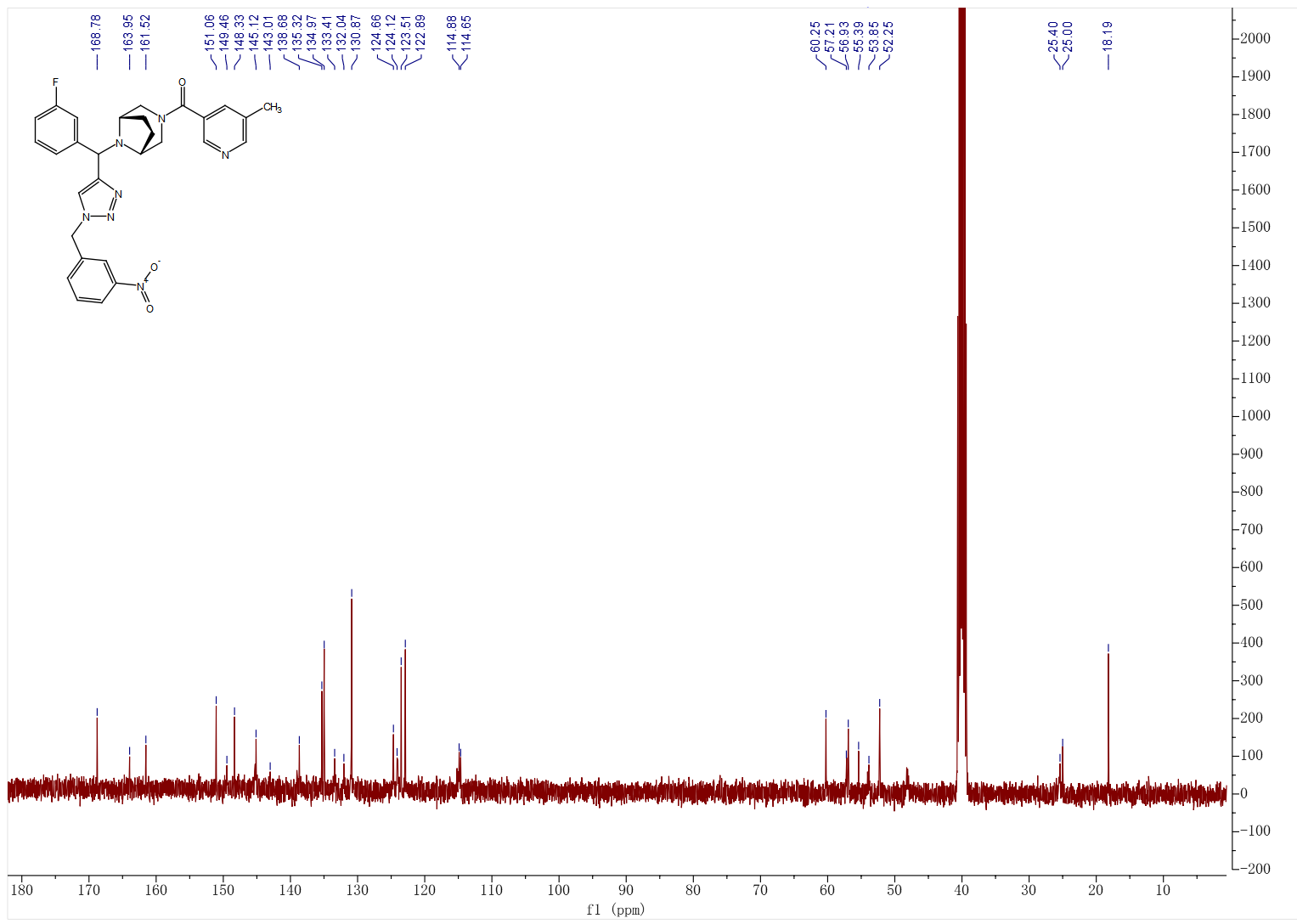


^1^H NMR (600 MHz, DMSO-*d*_6_) of **C5N6**


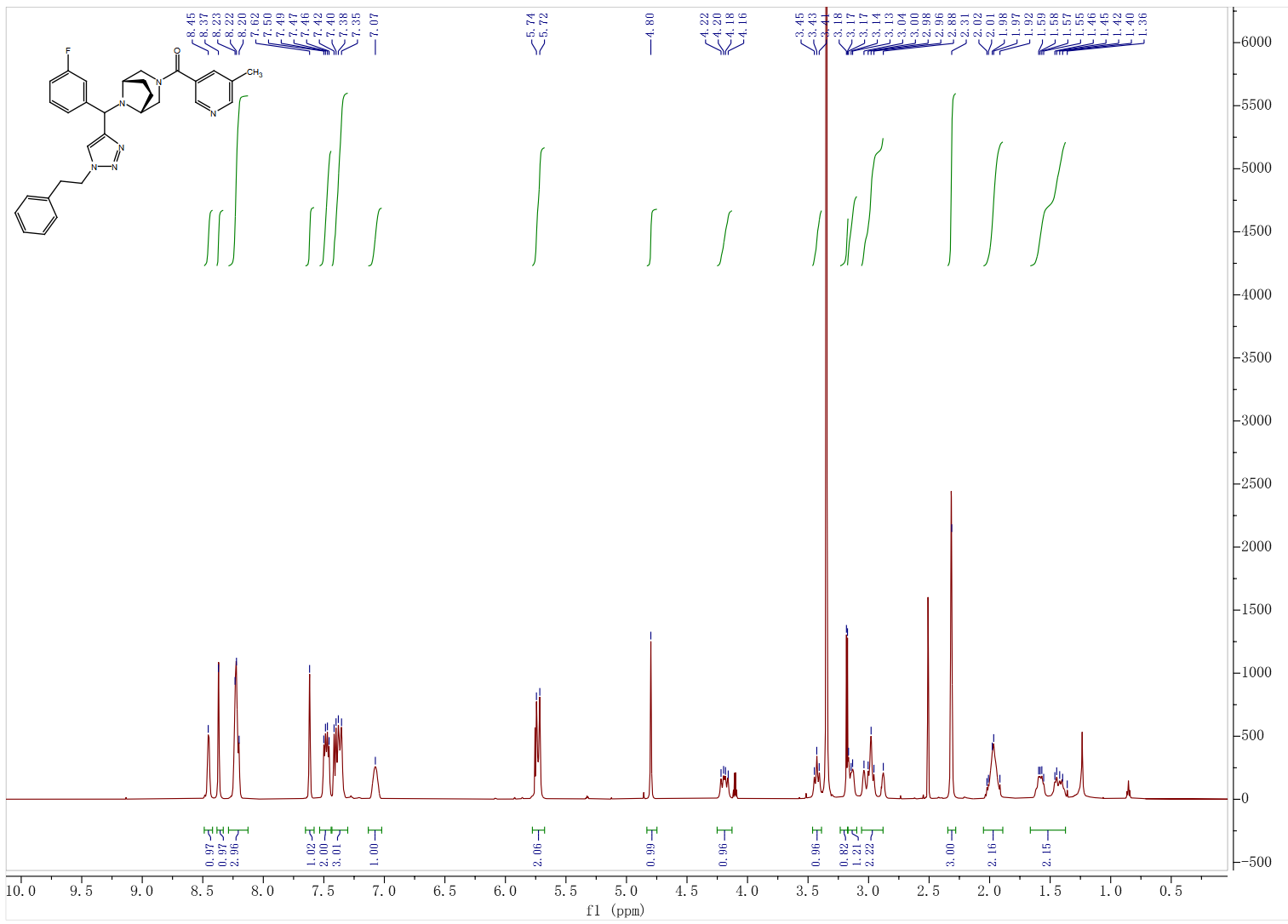


^13^C NMR (150 MHz, DMSO-*d*_6_) of **C5N6**


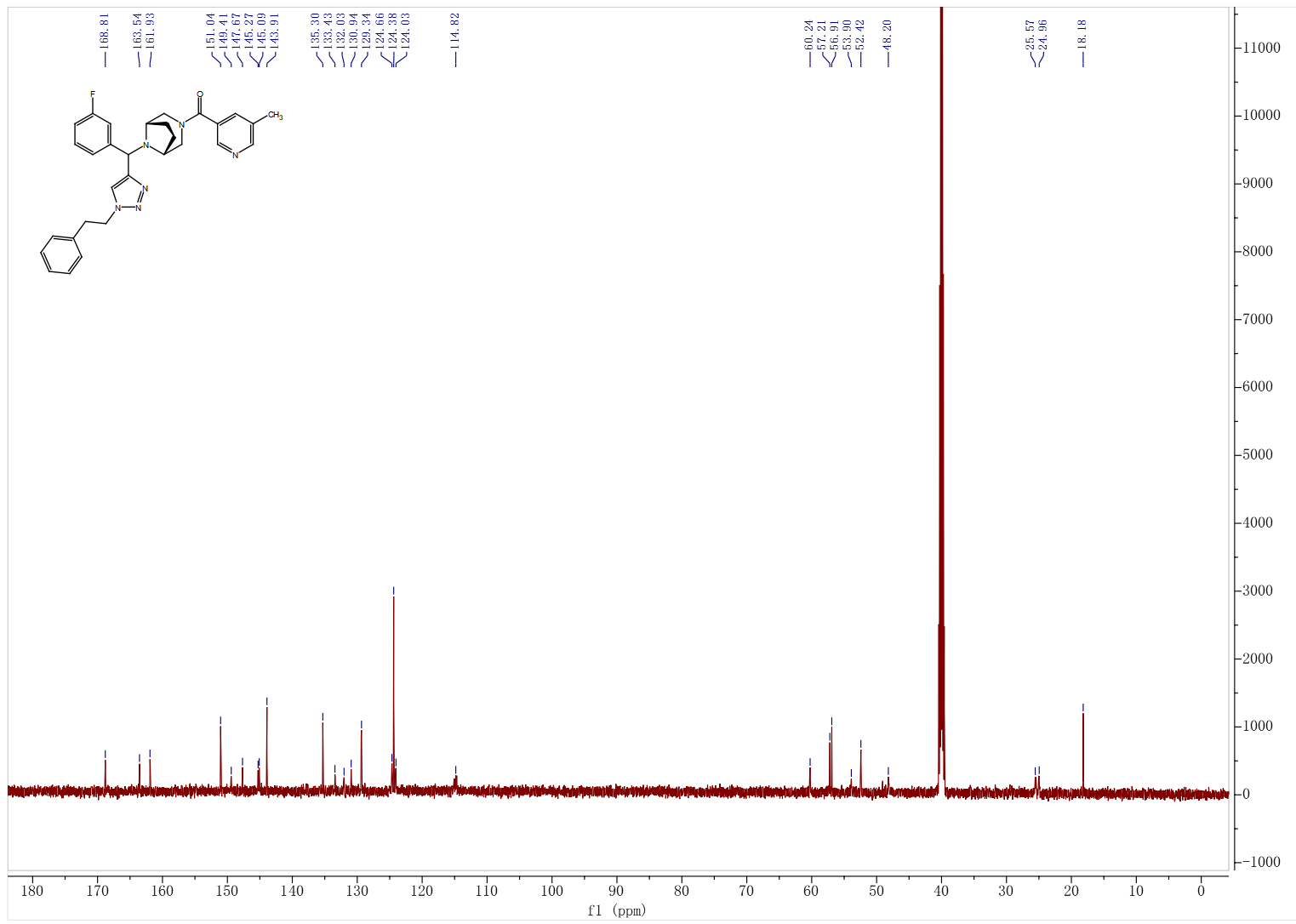


^1^H NMR (400 MHz, DMSO-*d*_6_) of **C5N16**


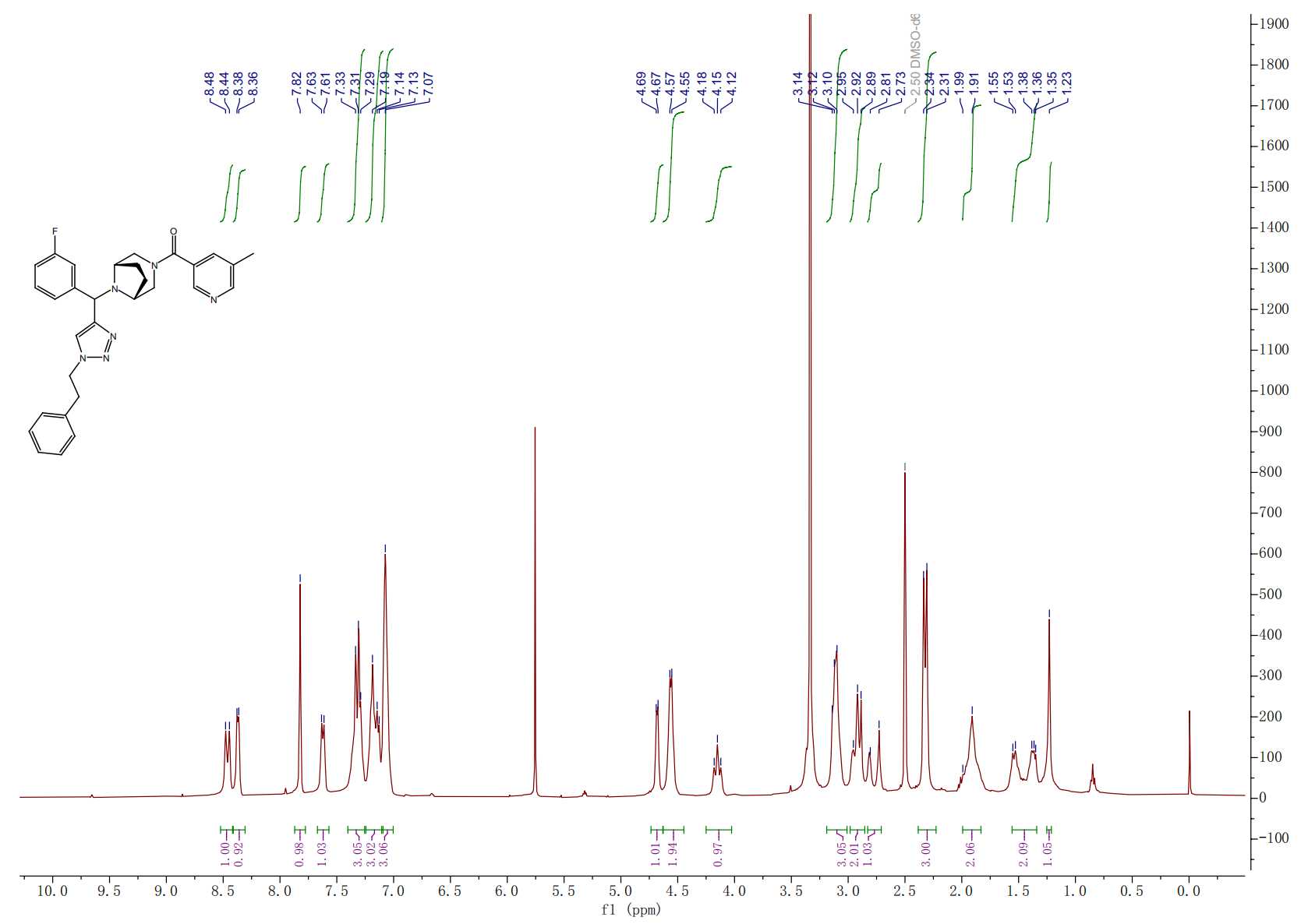


^13^C NMR (150 MHz, DMSO-*d*_6_) of **C5N16**


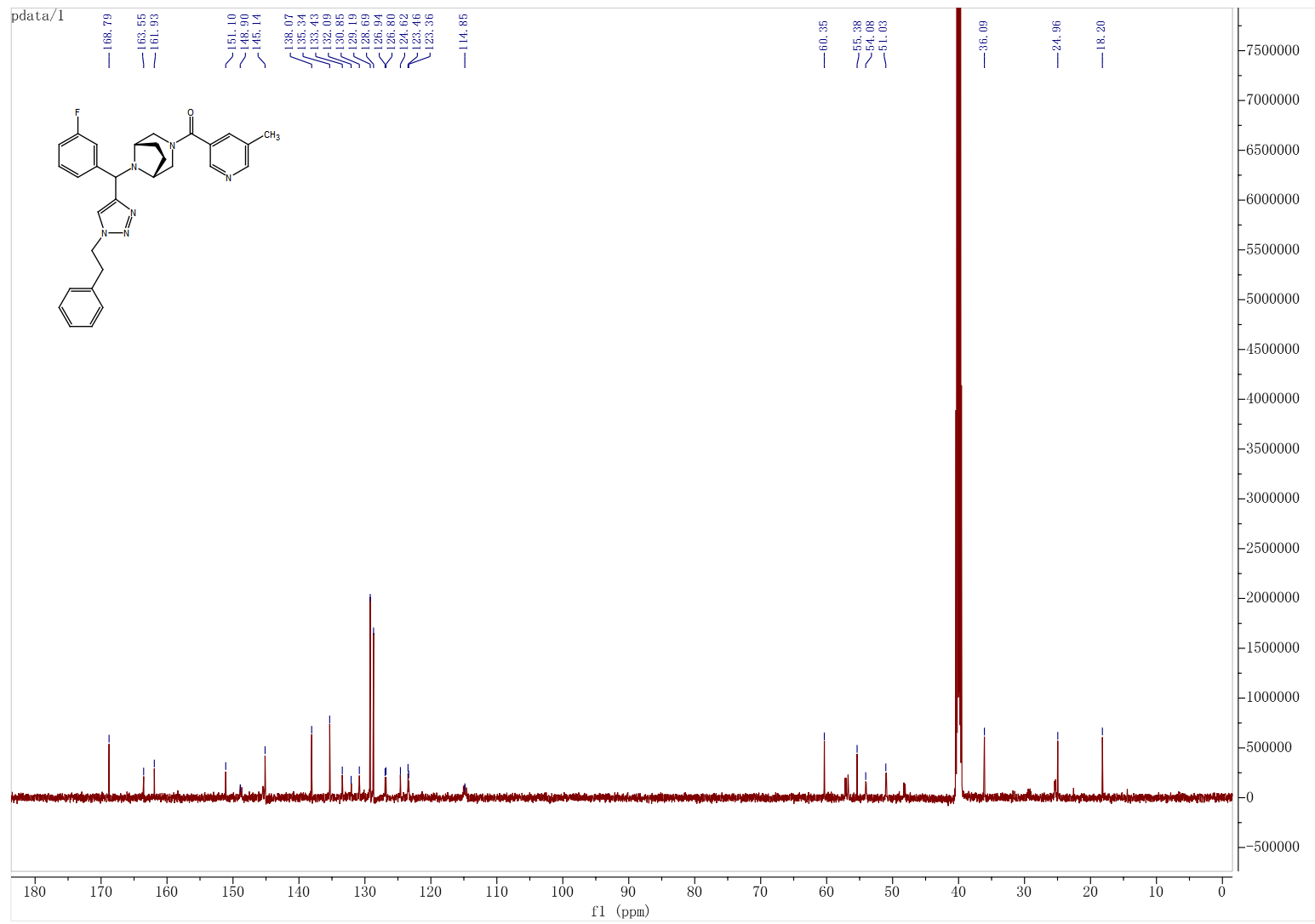


^1^H NMR (400 MHz, DMSO-*d*_6_) of **C5N17**


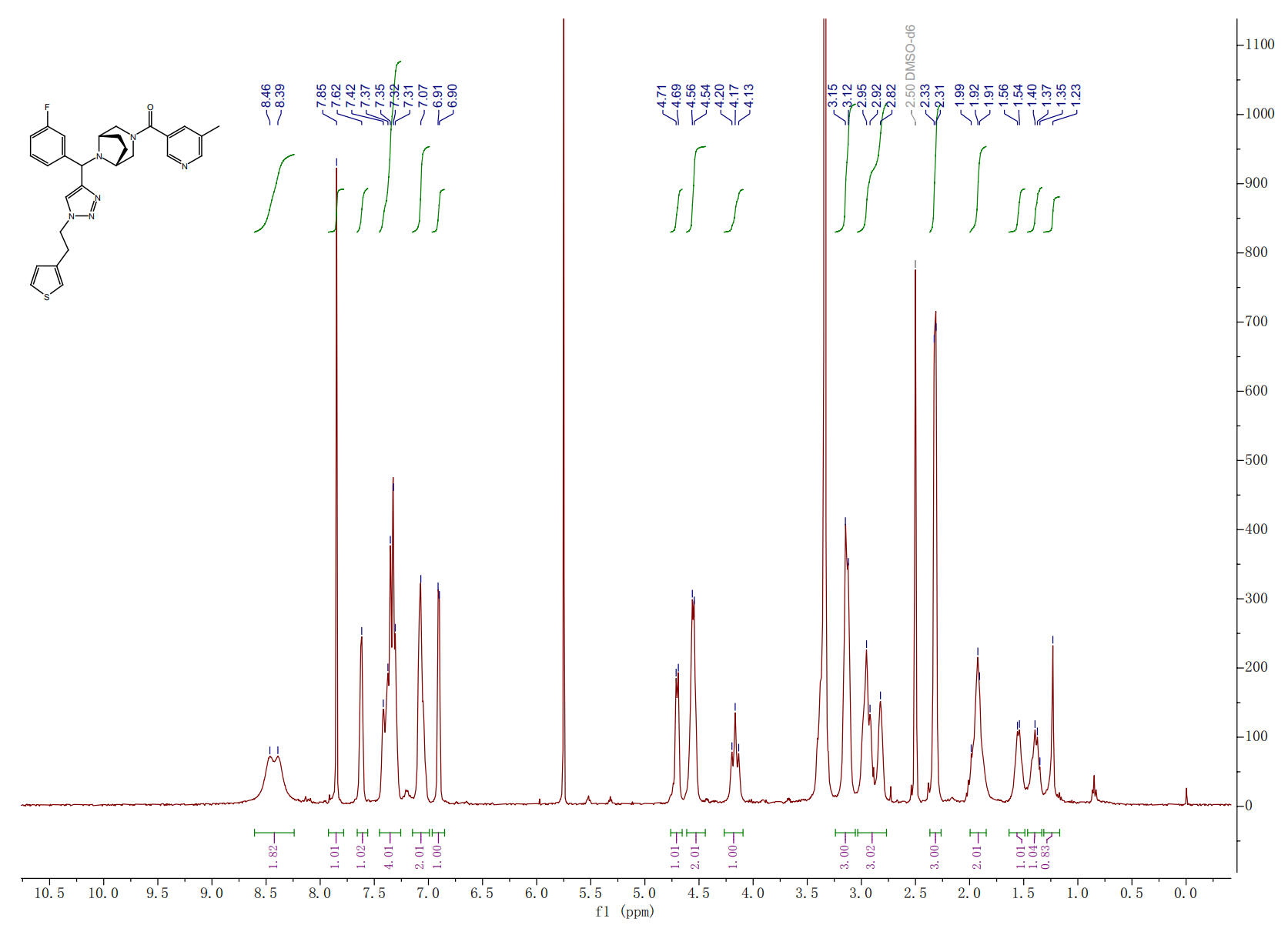


^13^C NMR (150 MHz, DMSO-*d*_6_) of **C5N17**


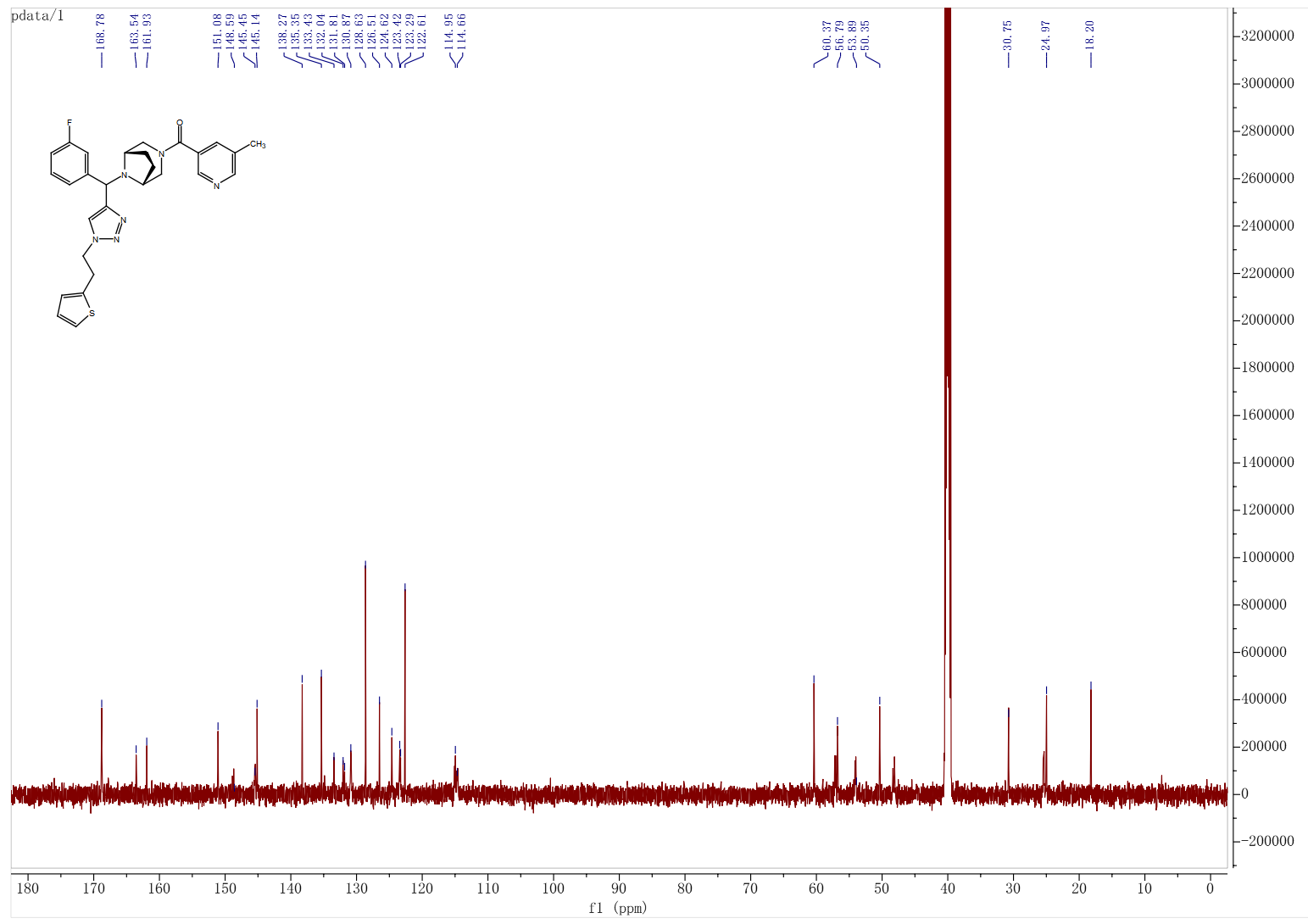


^1^H NMR (400 MHz, DMSO-*d*_6_) of **C5N21**


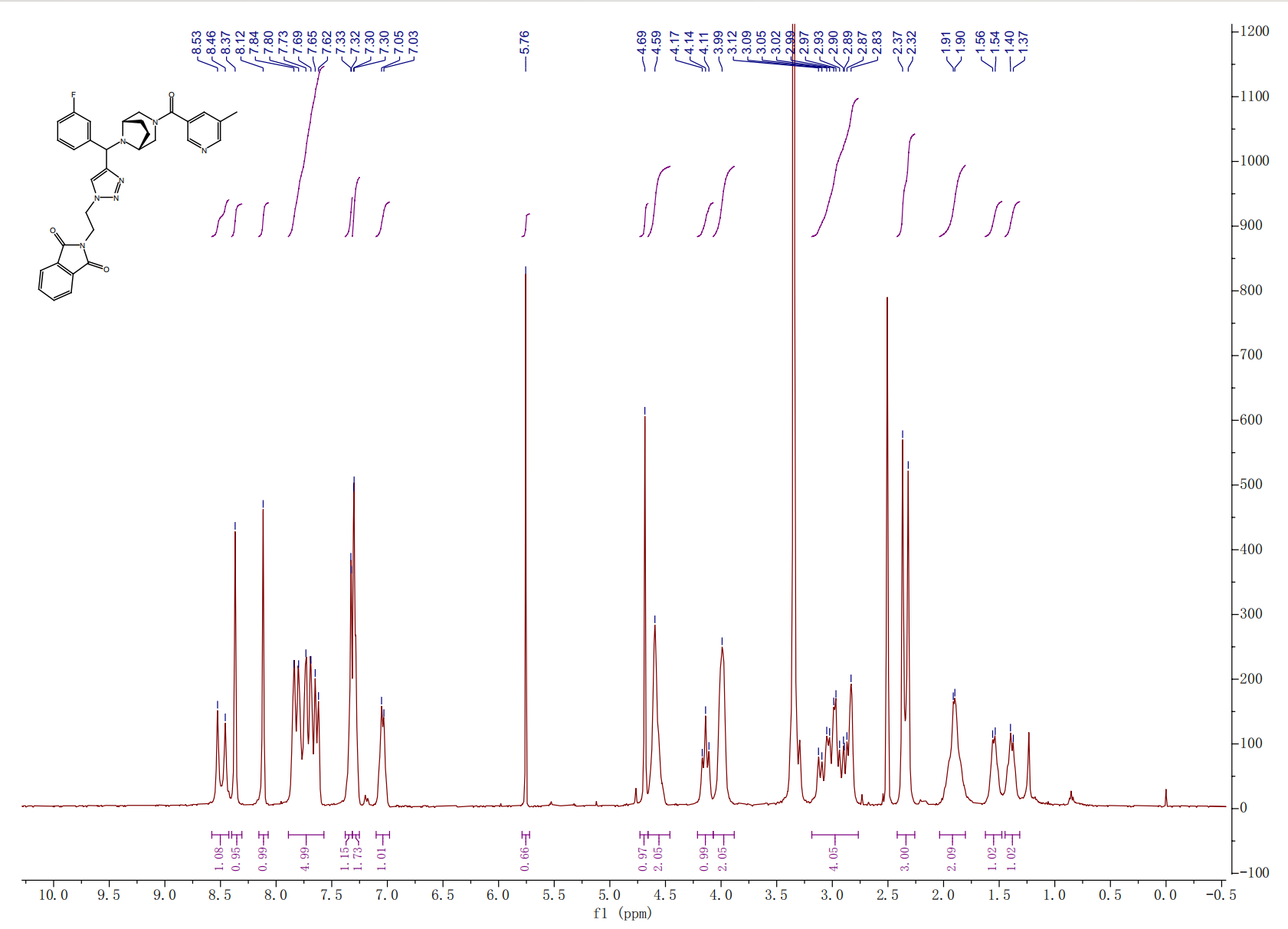


^13^C NMR (150 MHz, DMSO-*d*_6_) of **C5N21**


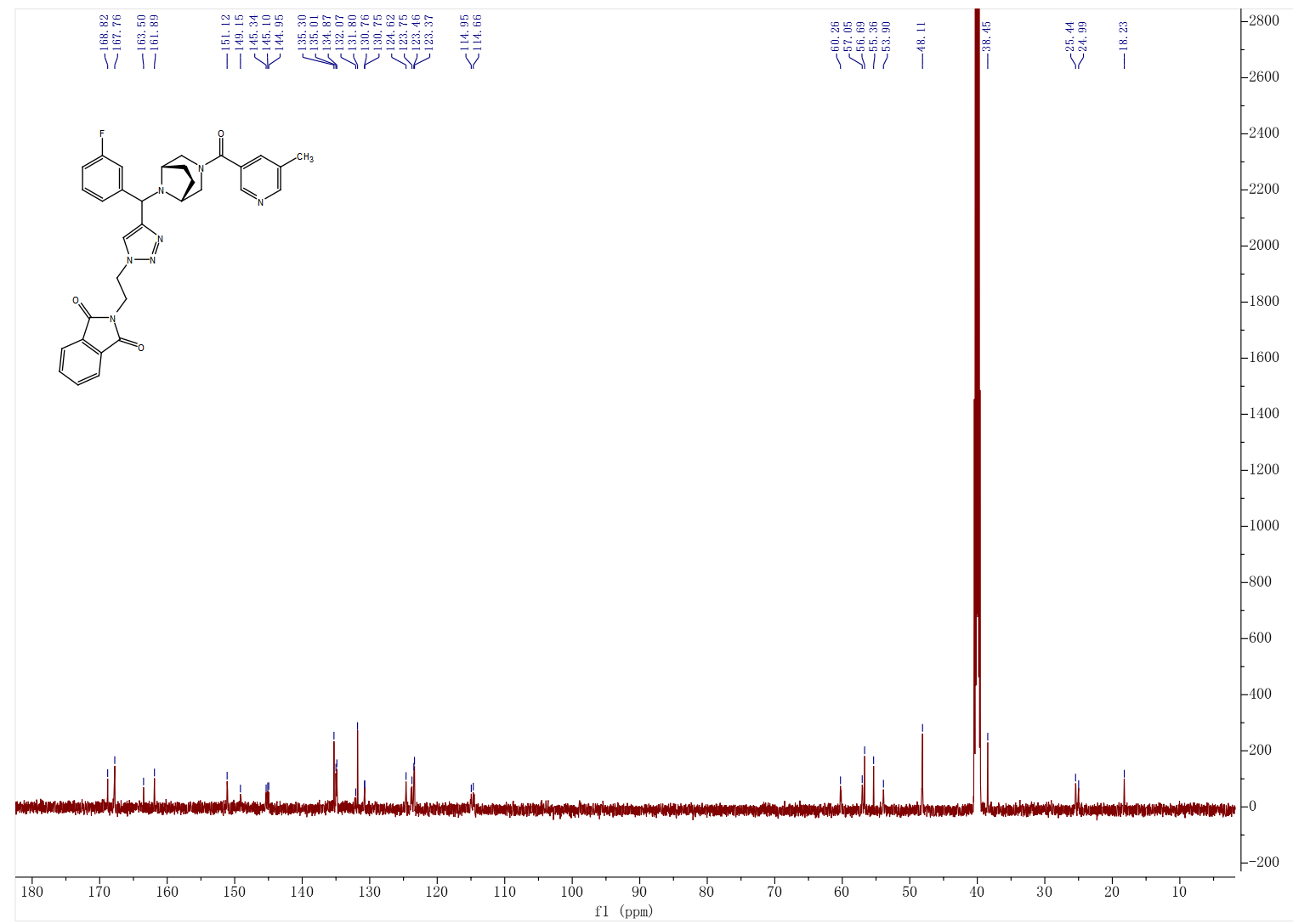


^1^H NMR (400 MHz, DMSO-*d*_6_) of **C5N39**


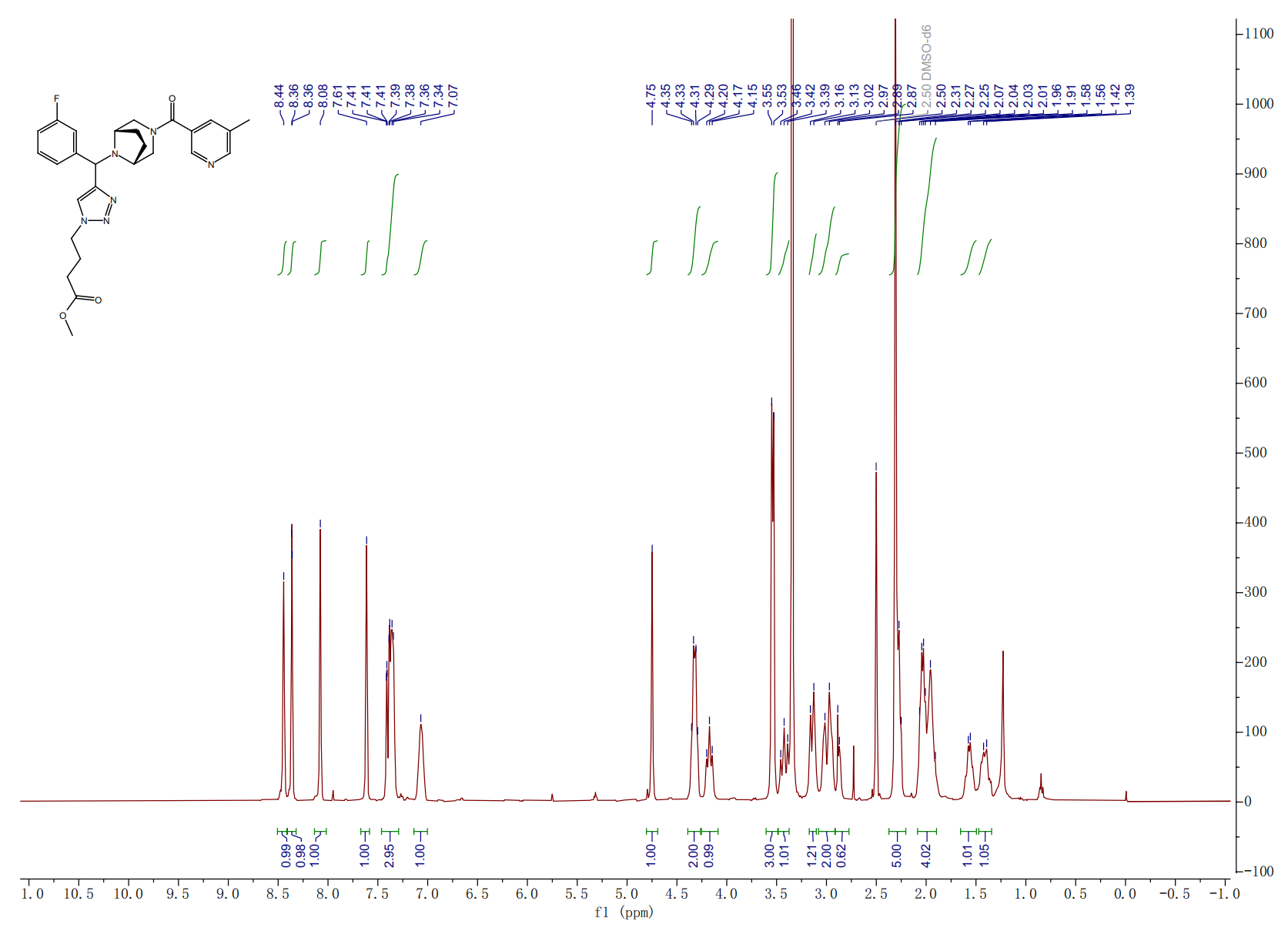


^13^C NMR (150 MHz, DMSO-*d*_6_) of **C5N39**


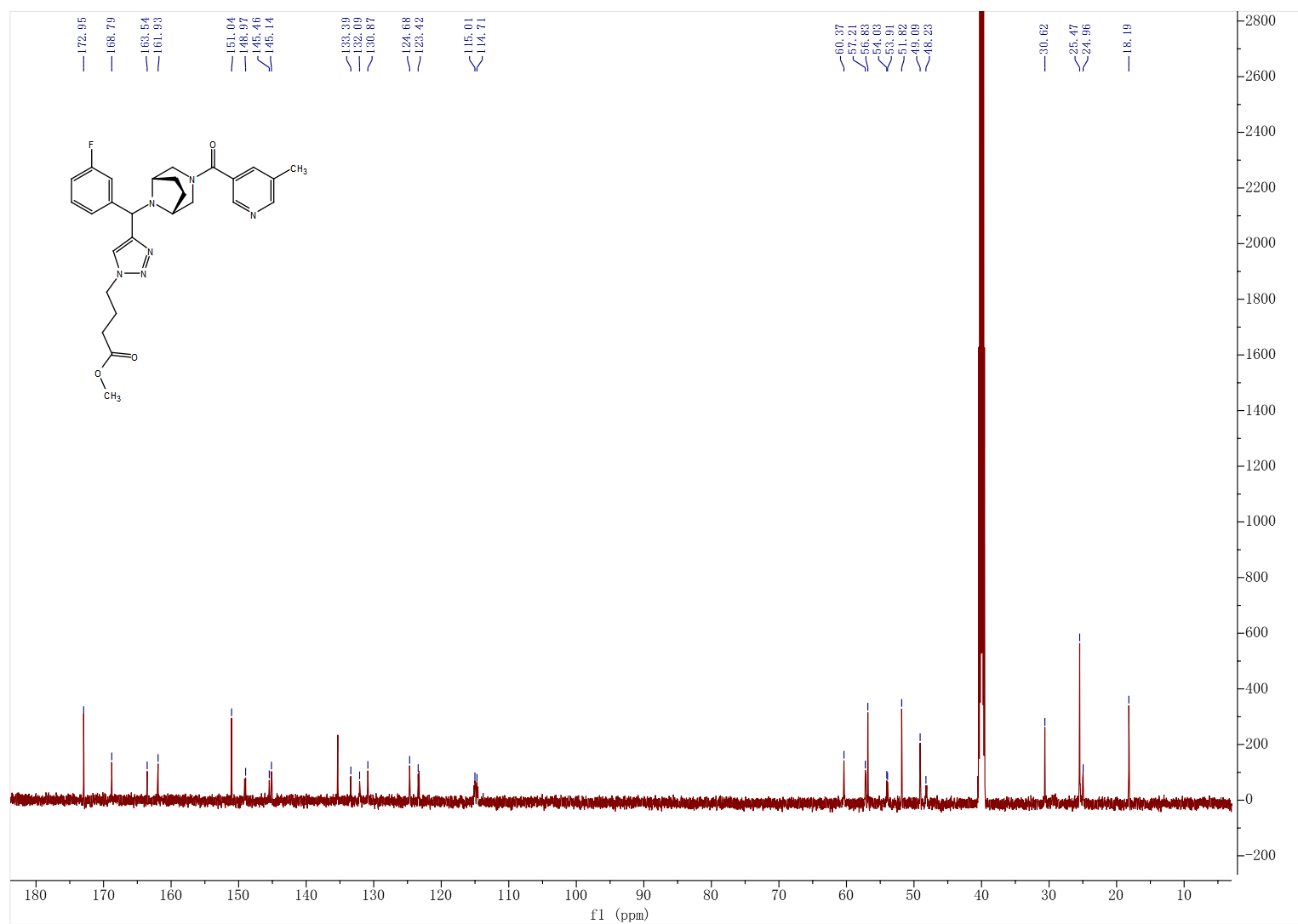


^1^H NMR (400 MHz, DMSO-*d*_6_) of **C5N41**


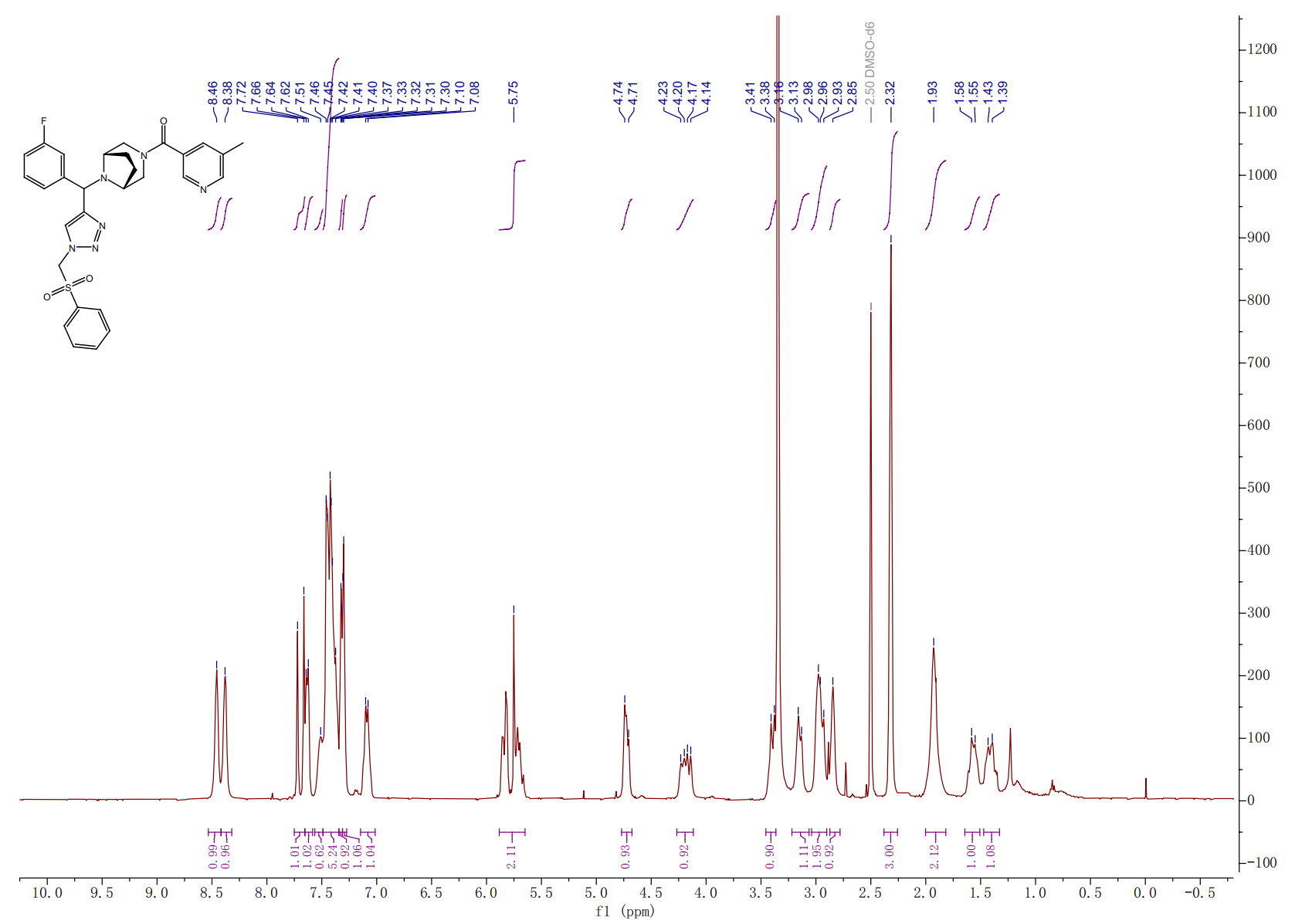


^13^C NMR (100 MHz, DMSO-*d*_6_) of **C5N41**


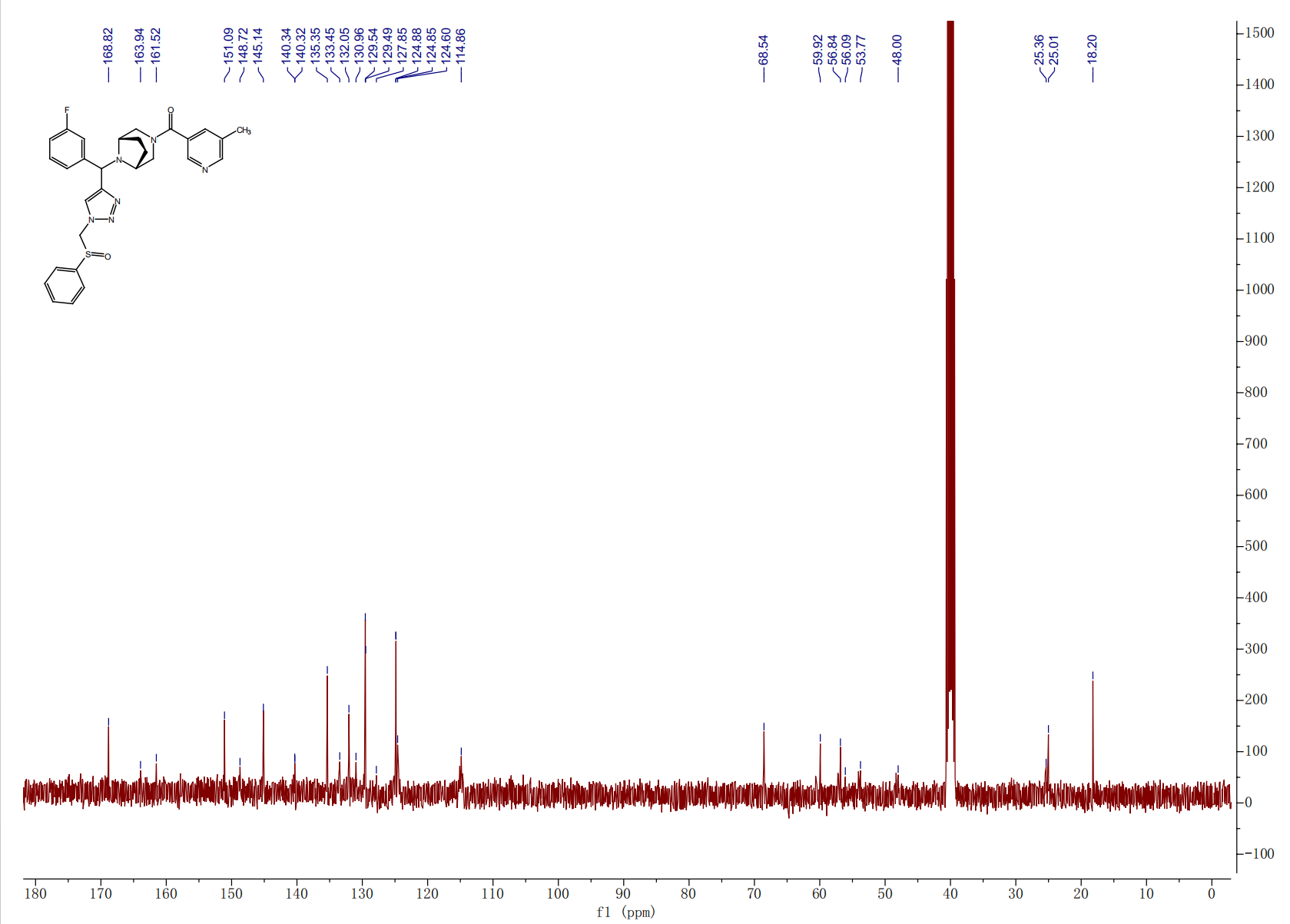


^1^H NMR (400 MHz, DMSO-*d*_6_) of **C5N42**


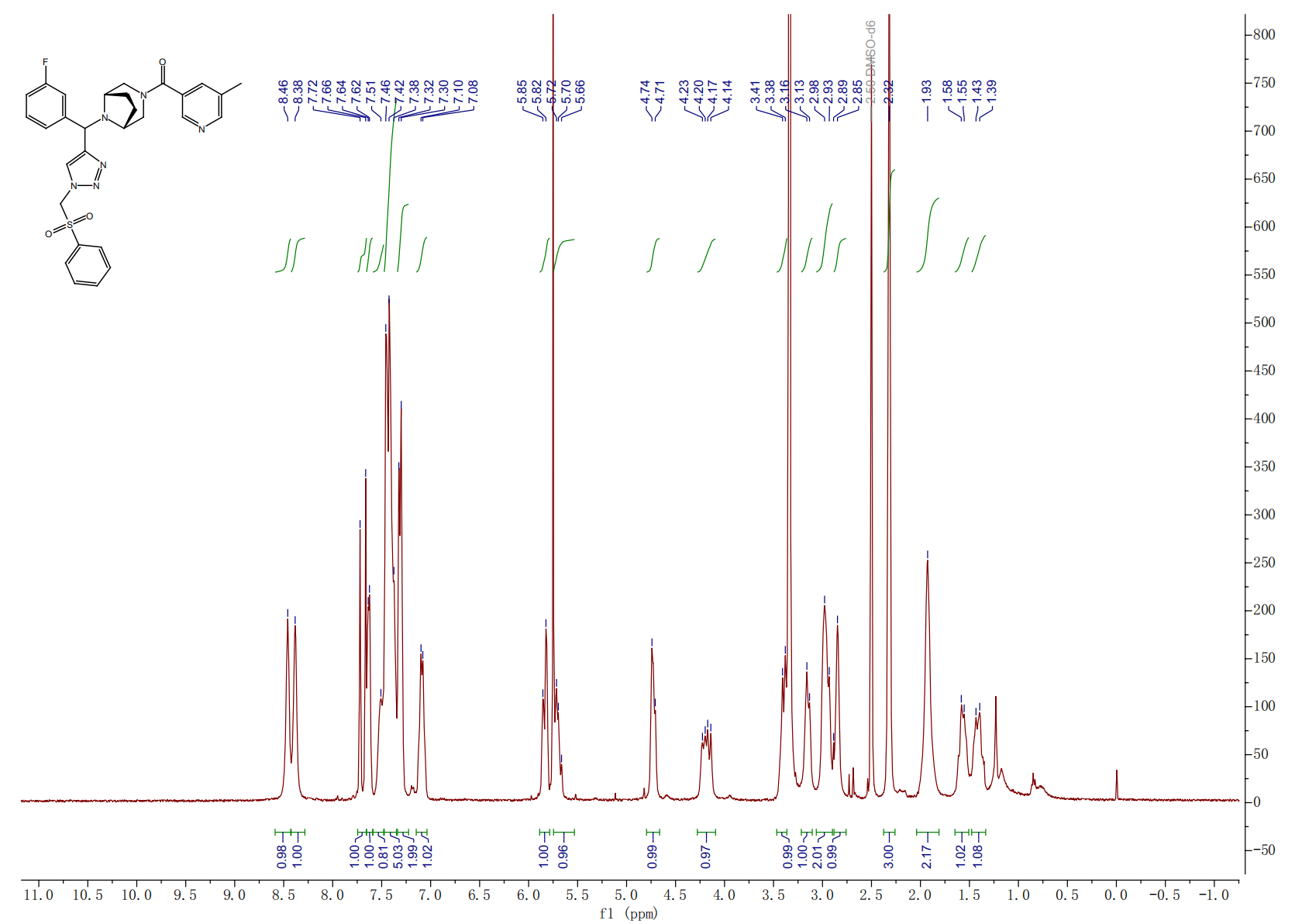


^13^C NMR (100 MHz, DMSO-*d*_6_) of **C5N42**


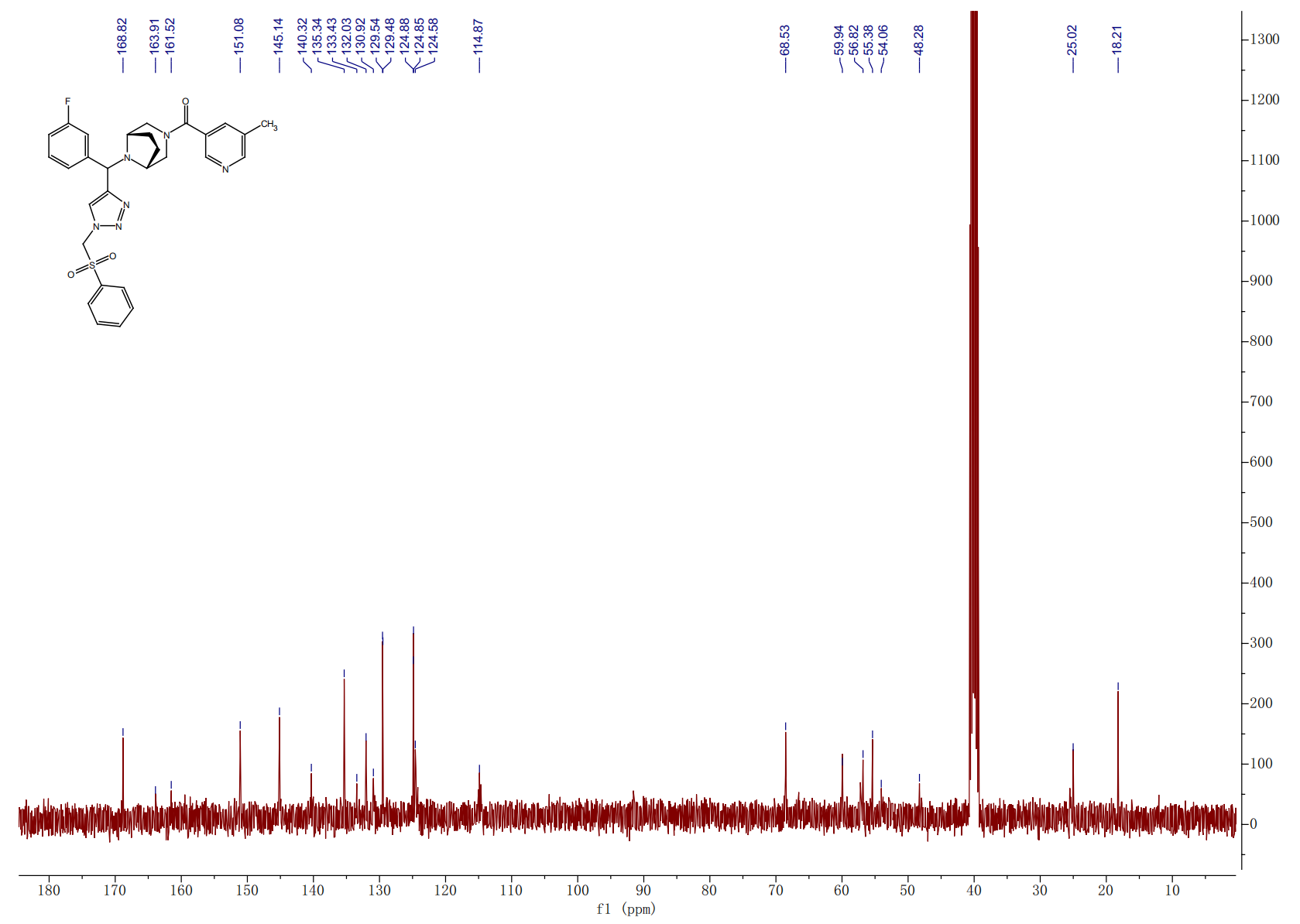


^1^H NMR (600 MHz, DMSO-*d*_6_) of **C5N50**


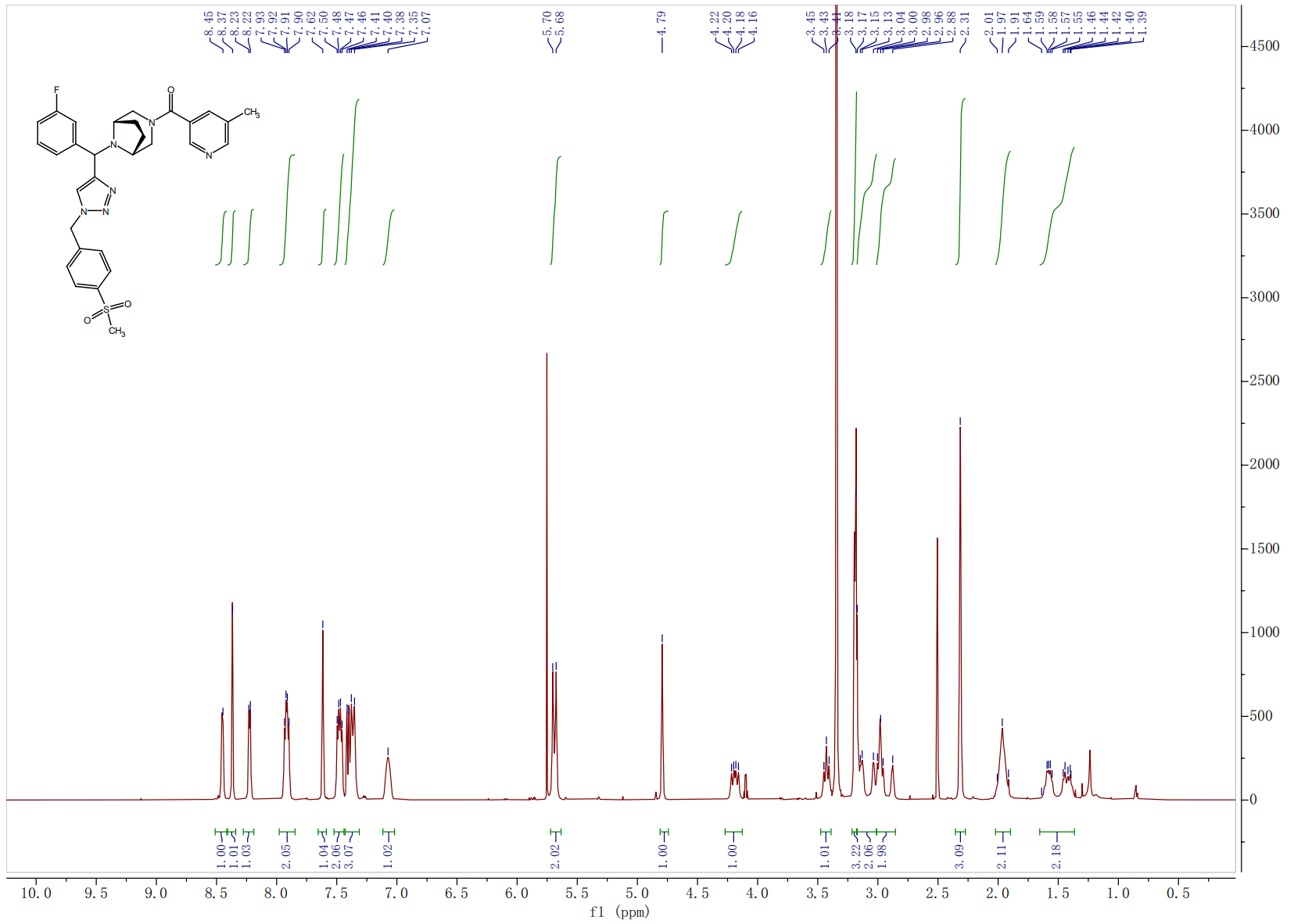


^13^C NMR (150 MHz, DMSO-*d*_6_) of **C5N50**


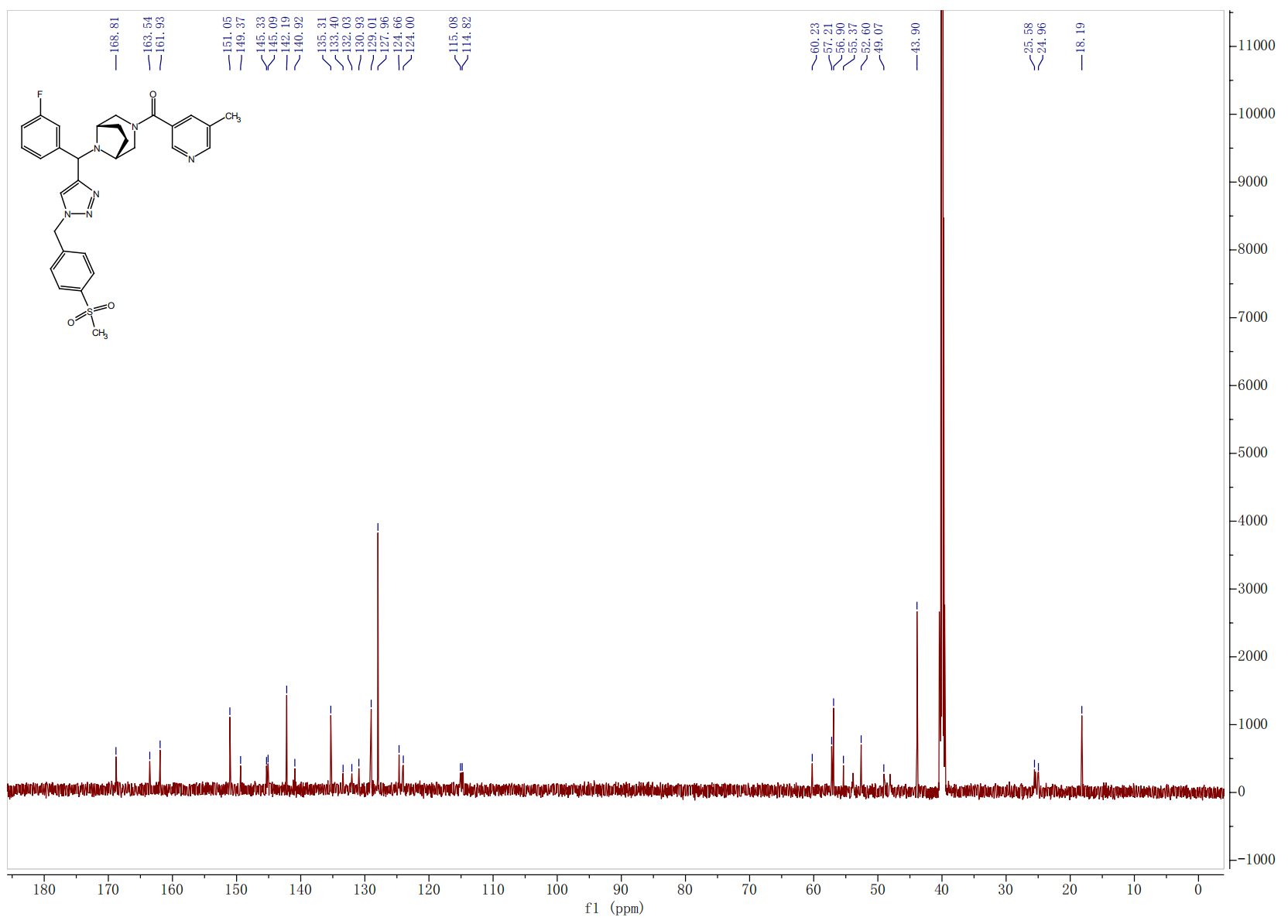


^1^H NMR (400 MHz, DMSO-*d*_6_) of **C5N57**


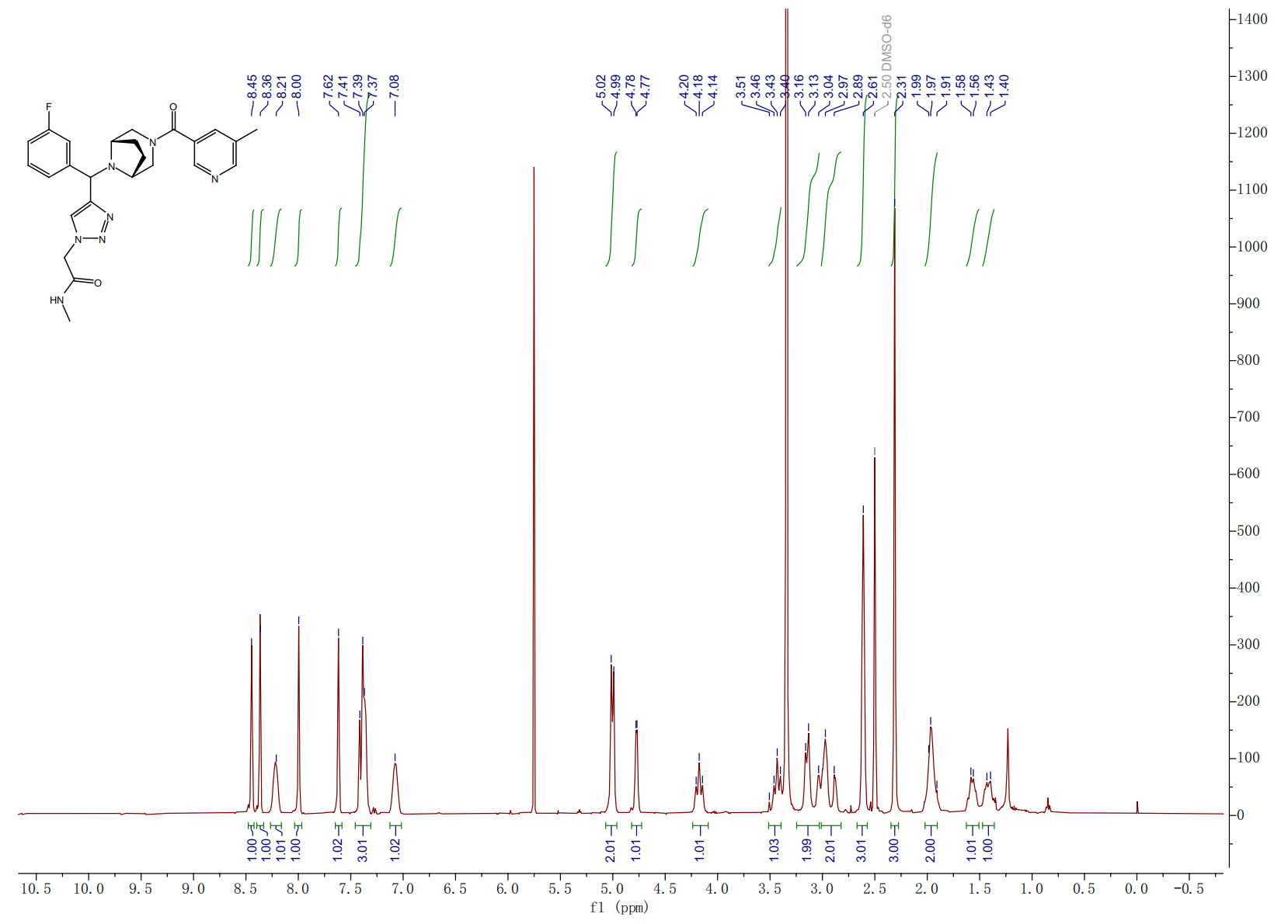


^13^C NMR (100 MHz, DMSO-*d*_6_) of **C5N57**


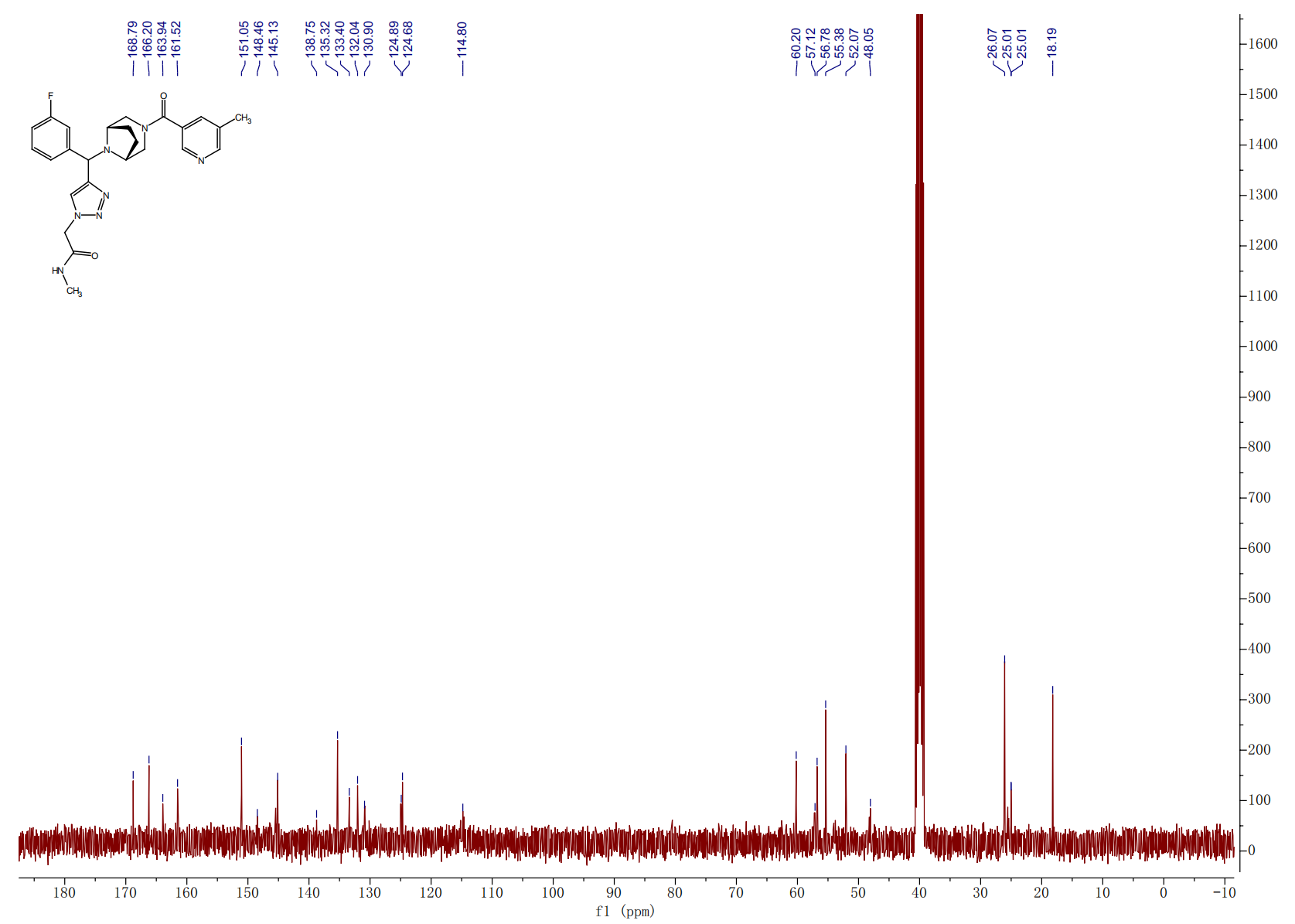


^1^H NMR (600 MHz, DMSO-*d*_6_) of **C5N58**


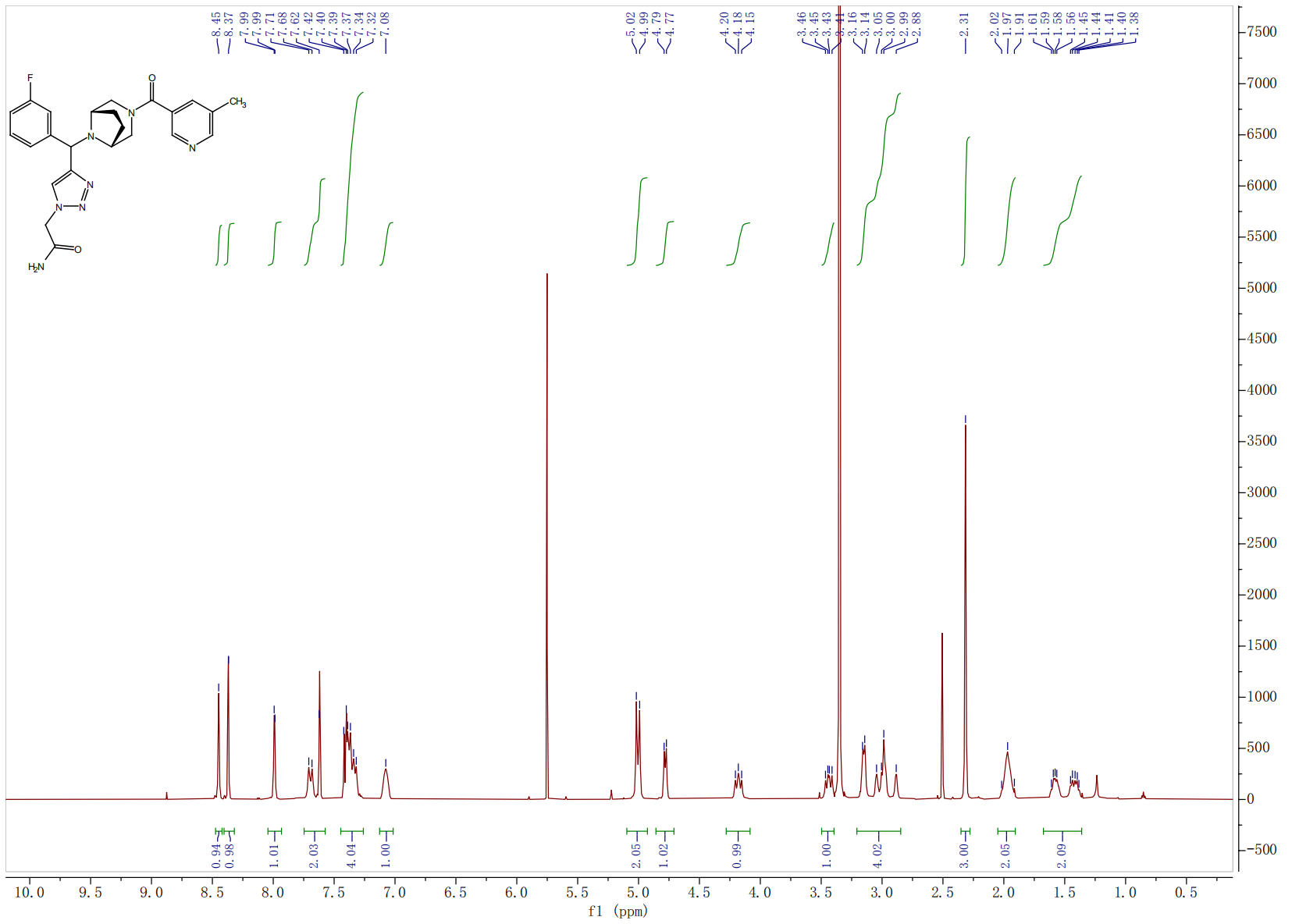


^13^C NMR (150 MHz, DMSO-*d*_6_) of **C5N58**


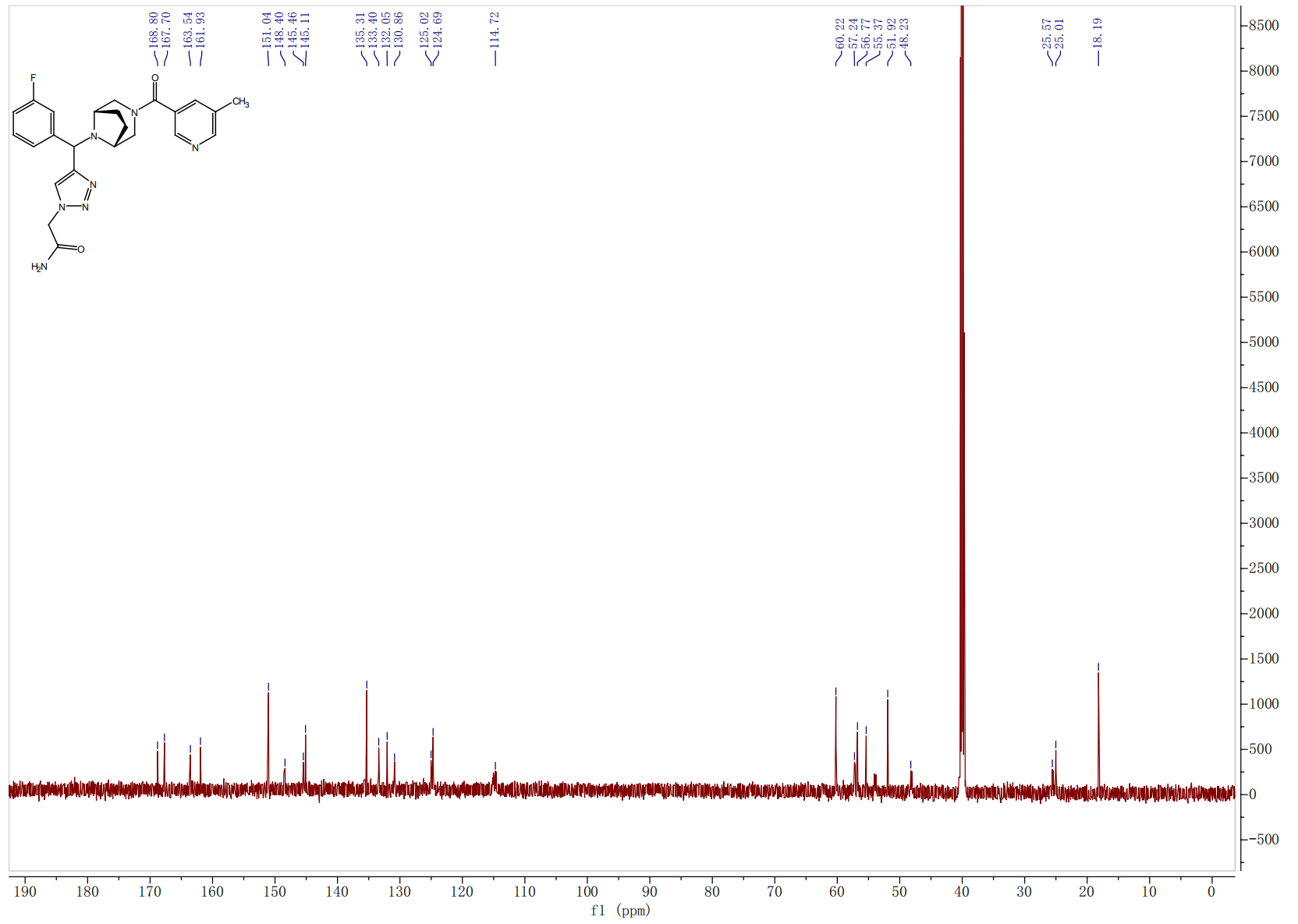


^1^H NMR (600 MHz, DMSO-*d*_6_) of **C5N64**


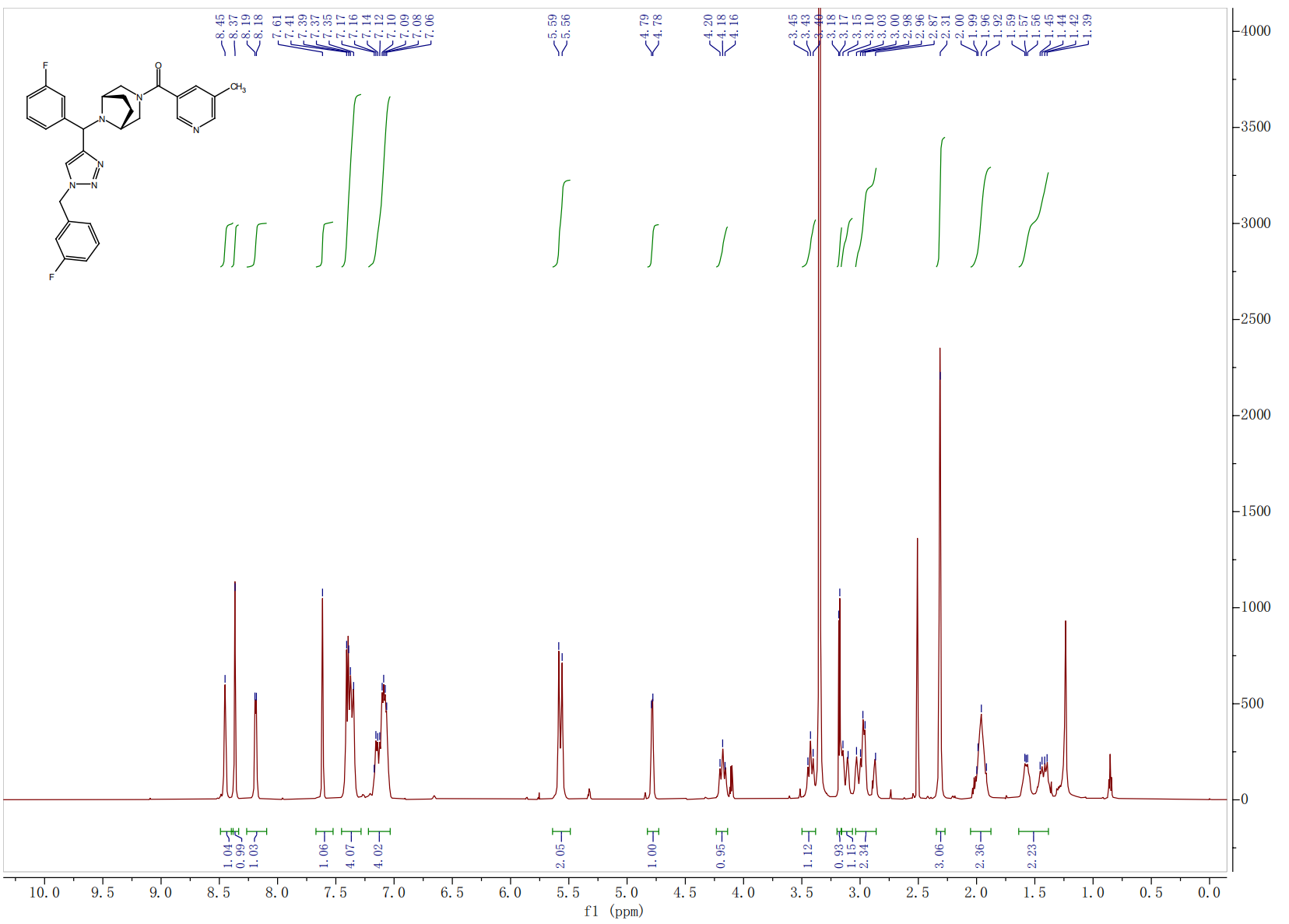


^13^C NMR (150 MHz, DMSO-*d*_6_) of **C5N64**


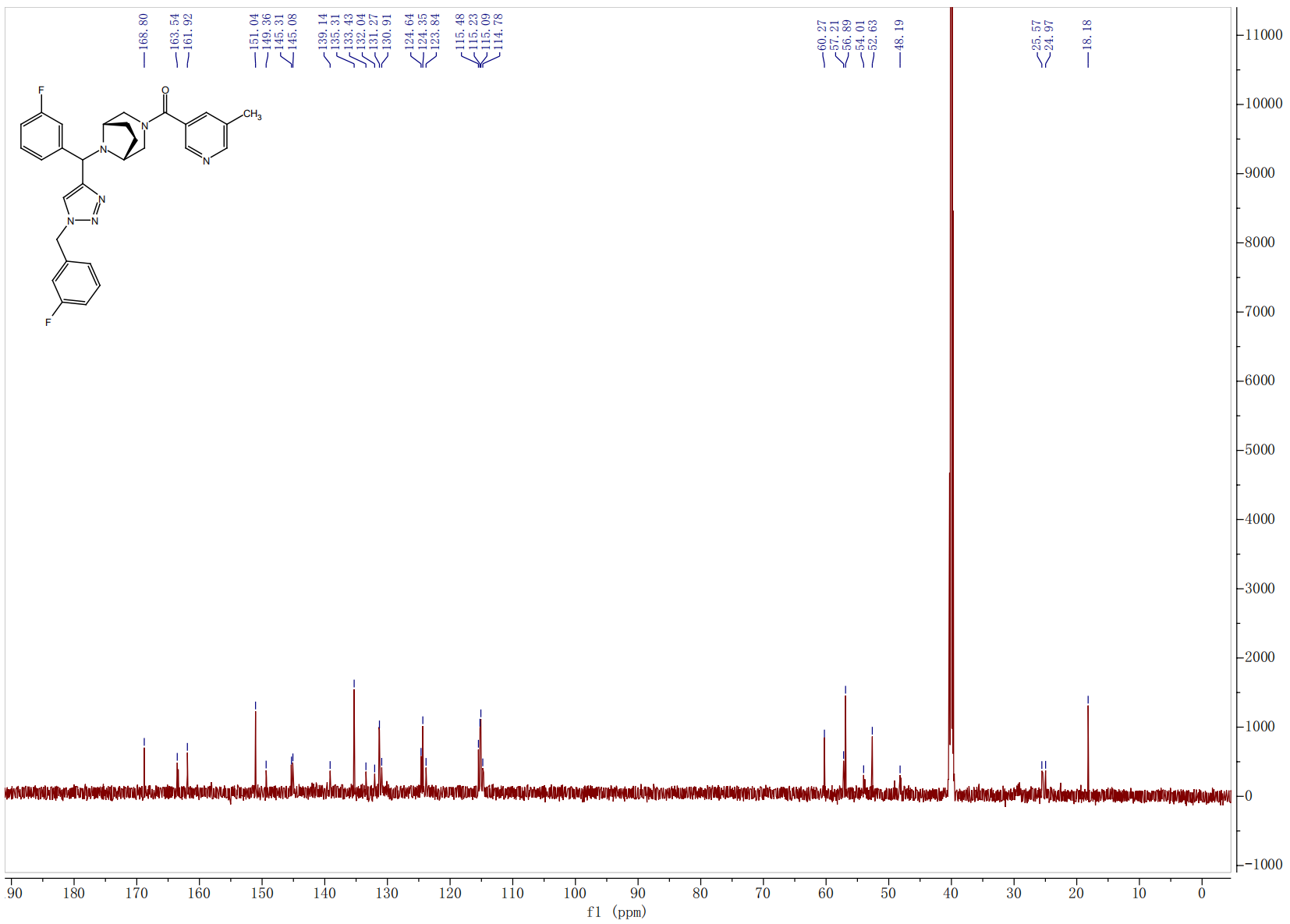


^1^H NMR (400 MHz, DMSO-*d*_6_) of **C4N17**


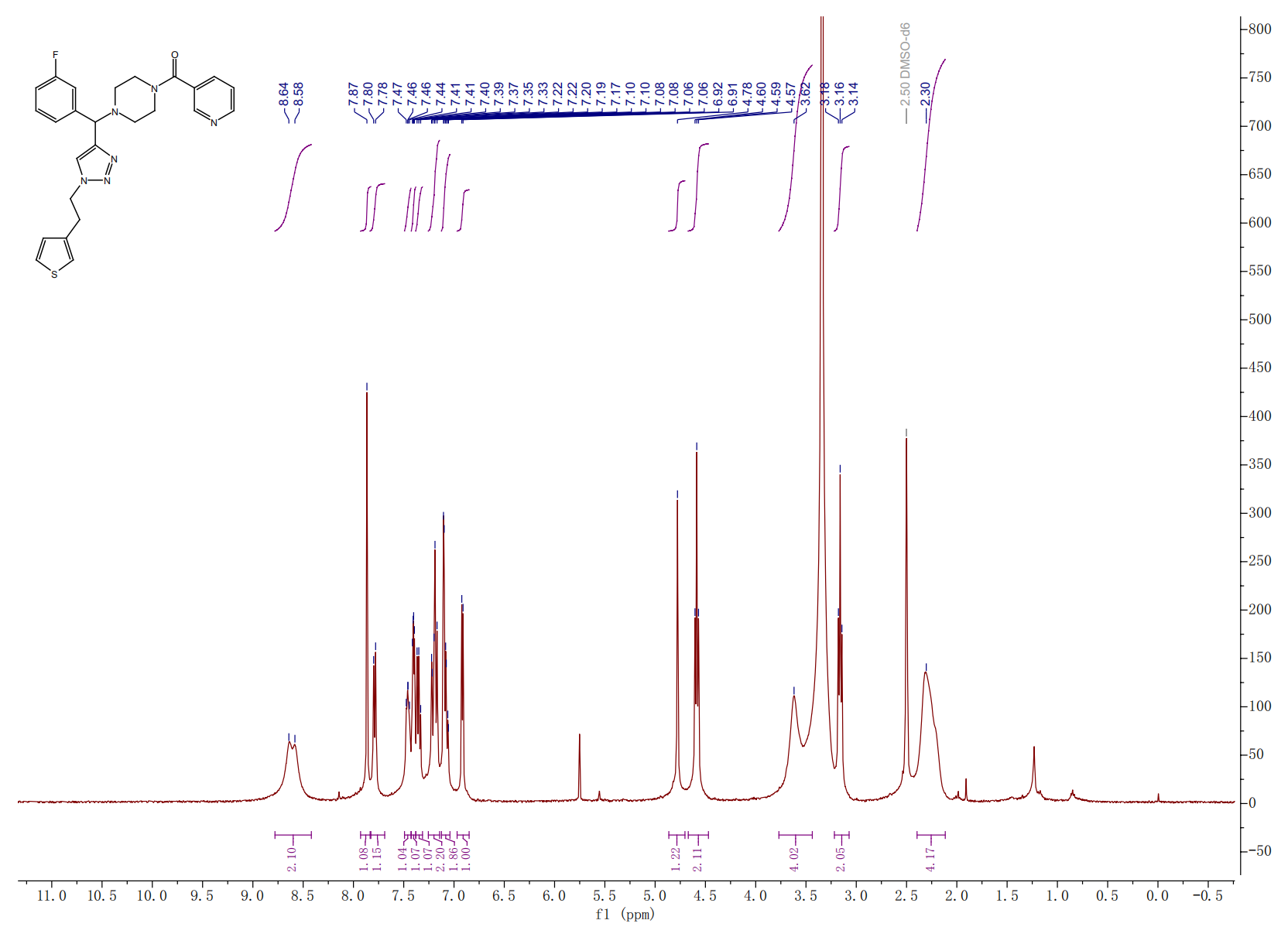


^13^C NMR (100 MHz, DMSO-*d*_6_) of **C4N17**


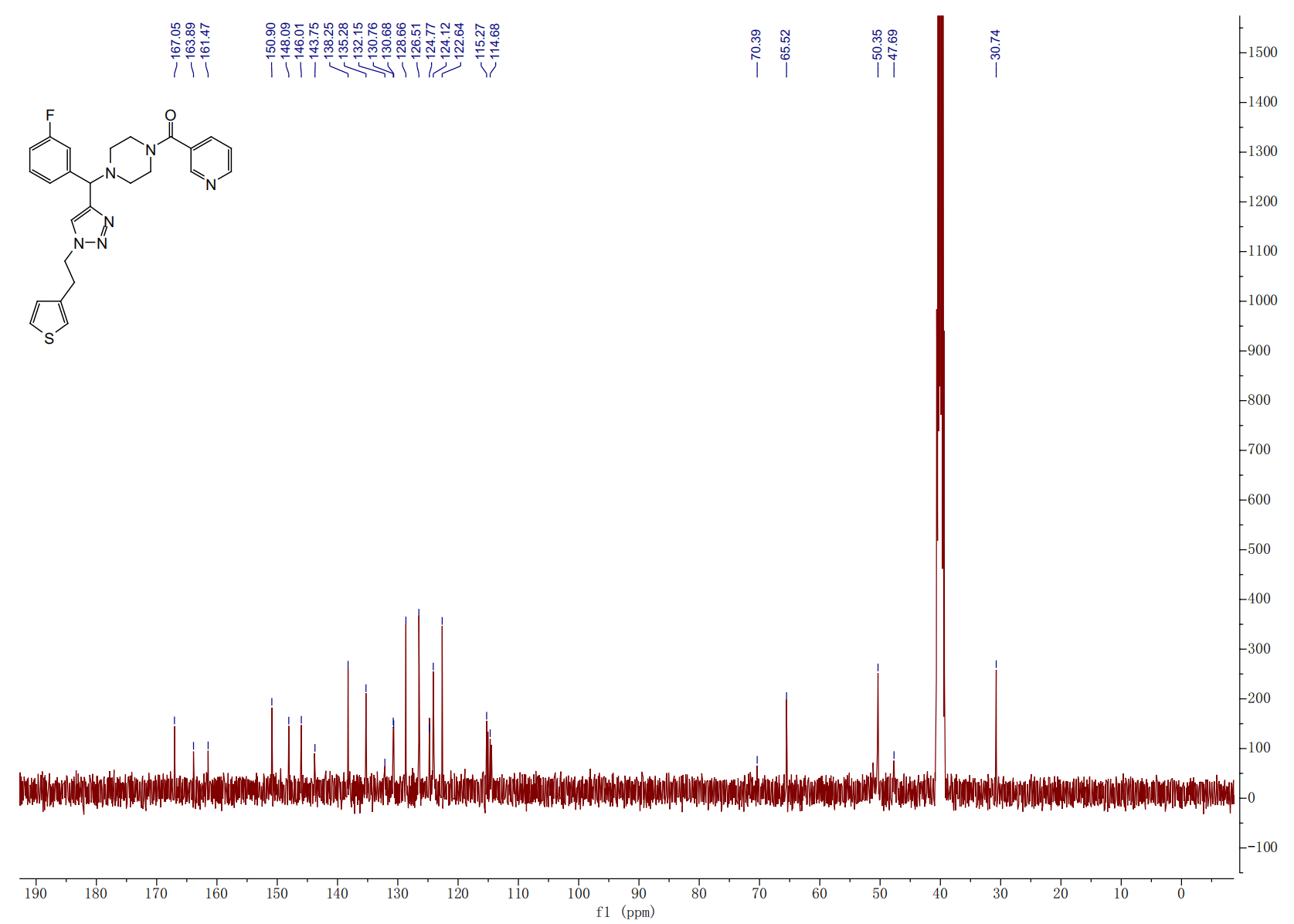


^1^H NMR (400 MHz, DMSO-*d*_6_) of **C4N21**


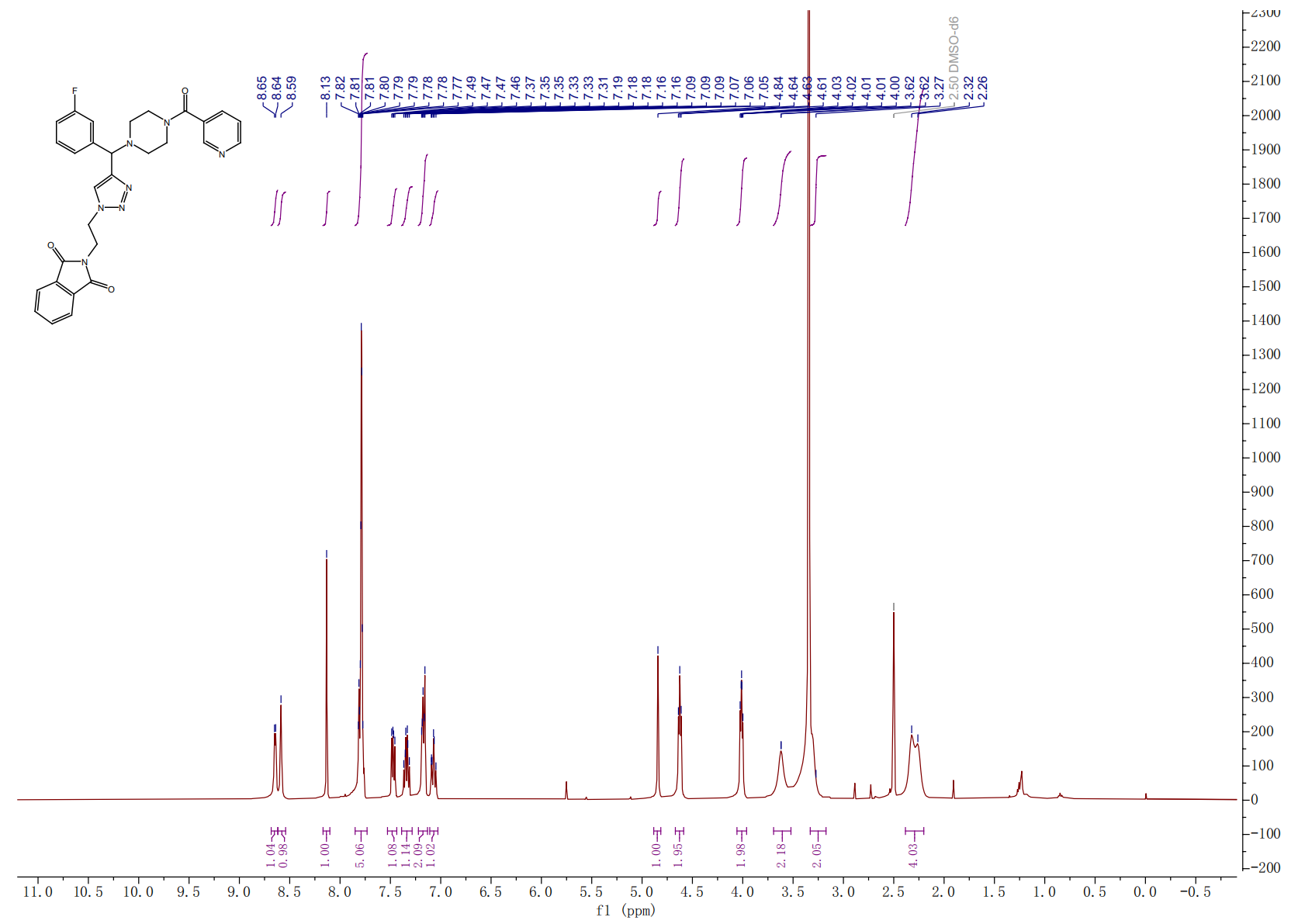


^13^C NMR (150 MHz, DMSO-*d*_6_) of **C4N21**


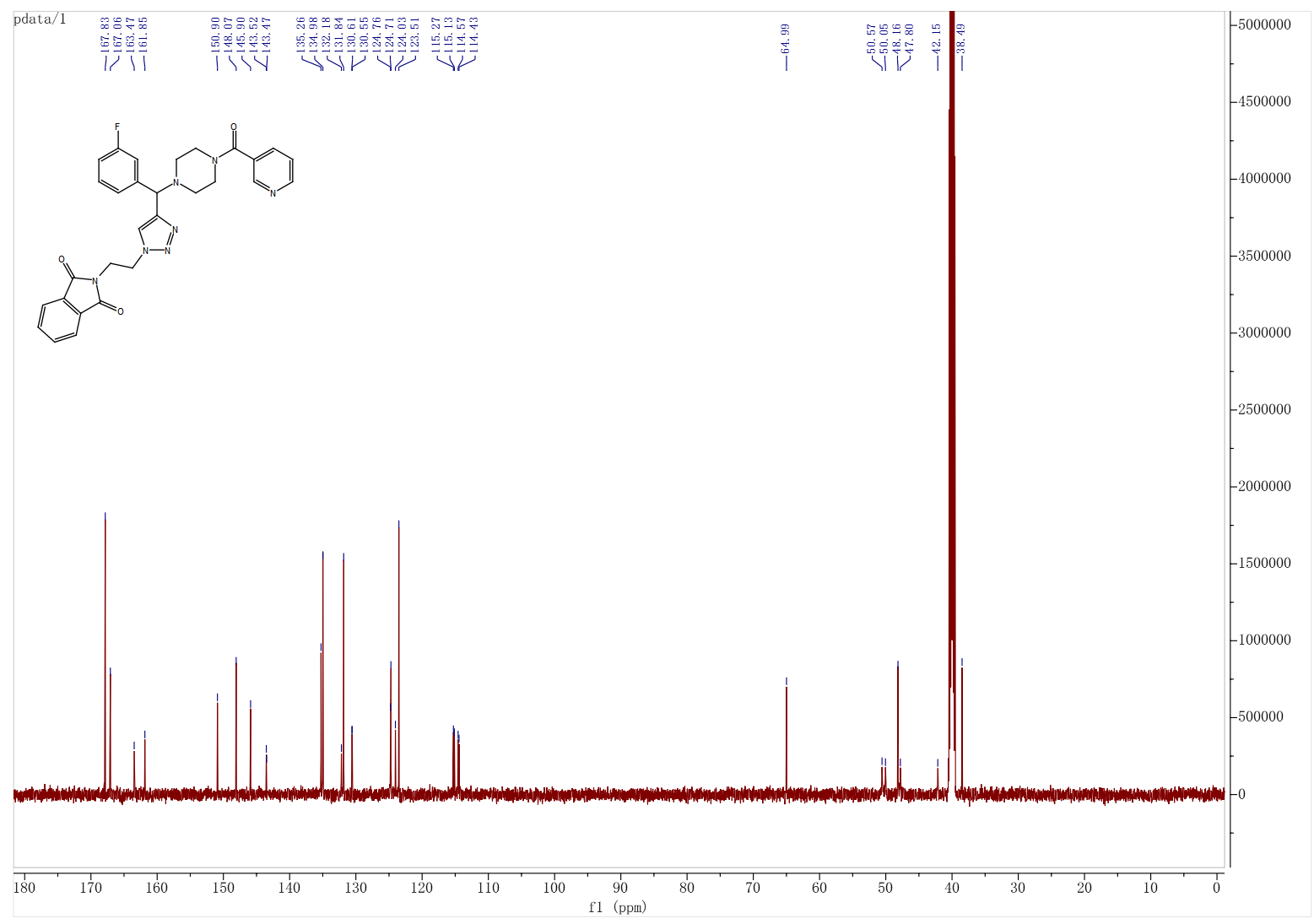


^1^H NMR (400 MHz, DMSO-*d*_6_) of **C6N17**


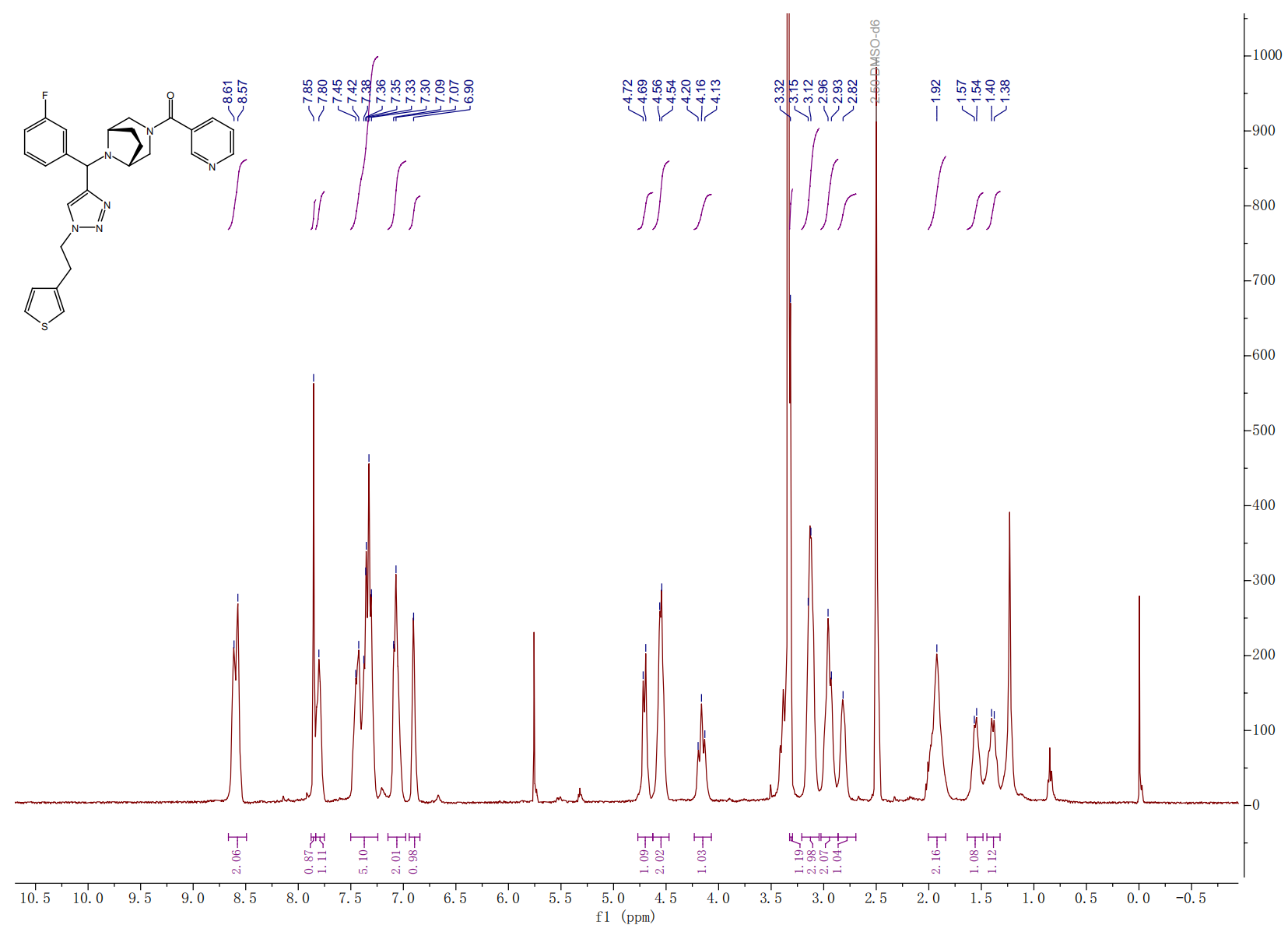


^13^C NMR (100 MHz, DMSO-*d*_6_) of **C6N17**


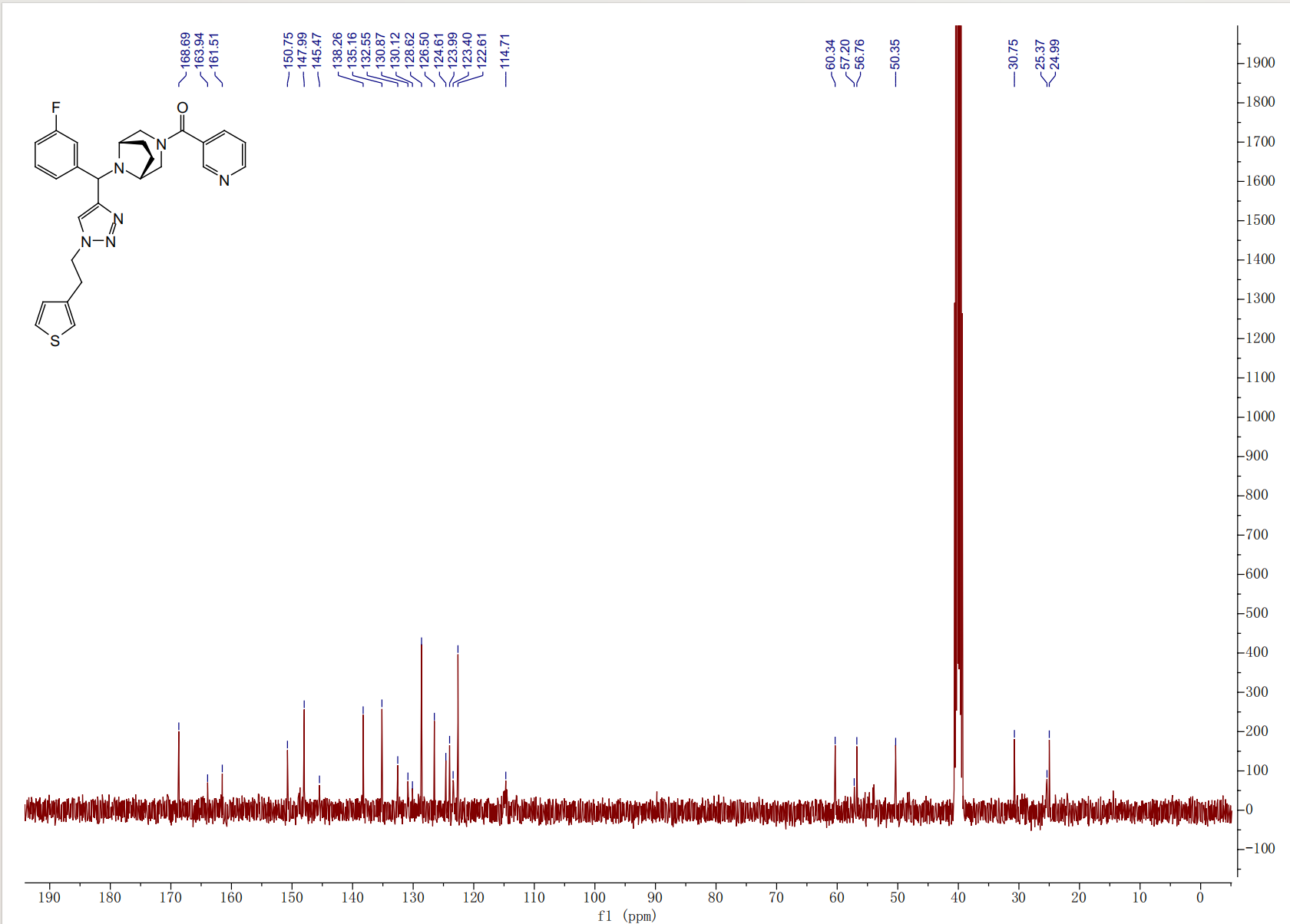


^1^H NMR (400 MHz, DMSO-*d*_6_) of **C6N21**


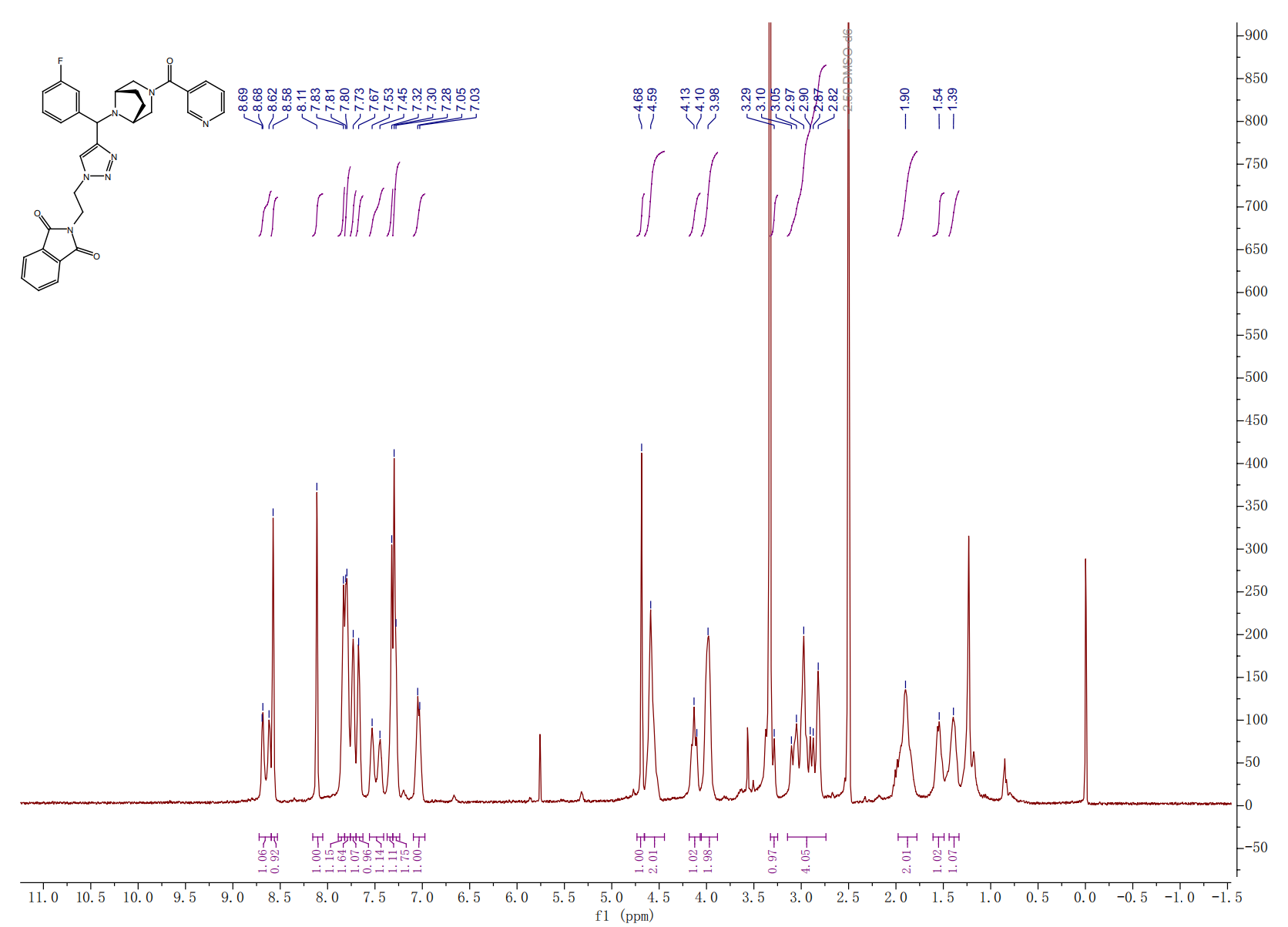


^13^C NMR (150 MHz, DMSO-*d*_6_) of **C6N21**


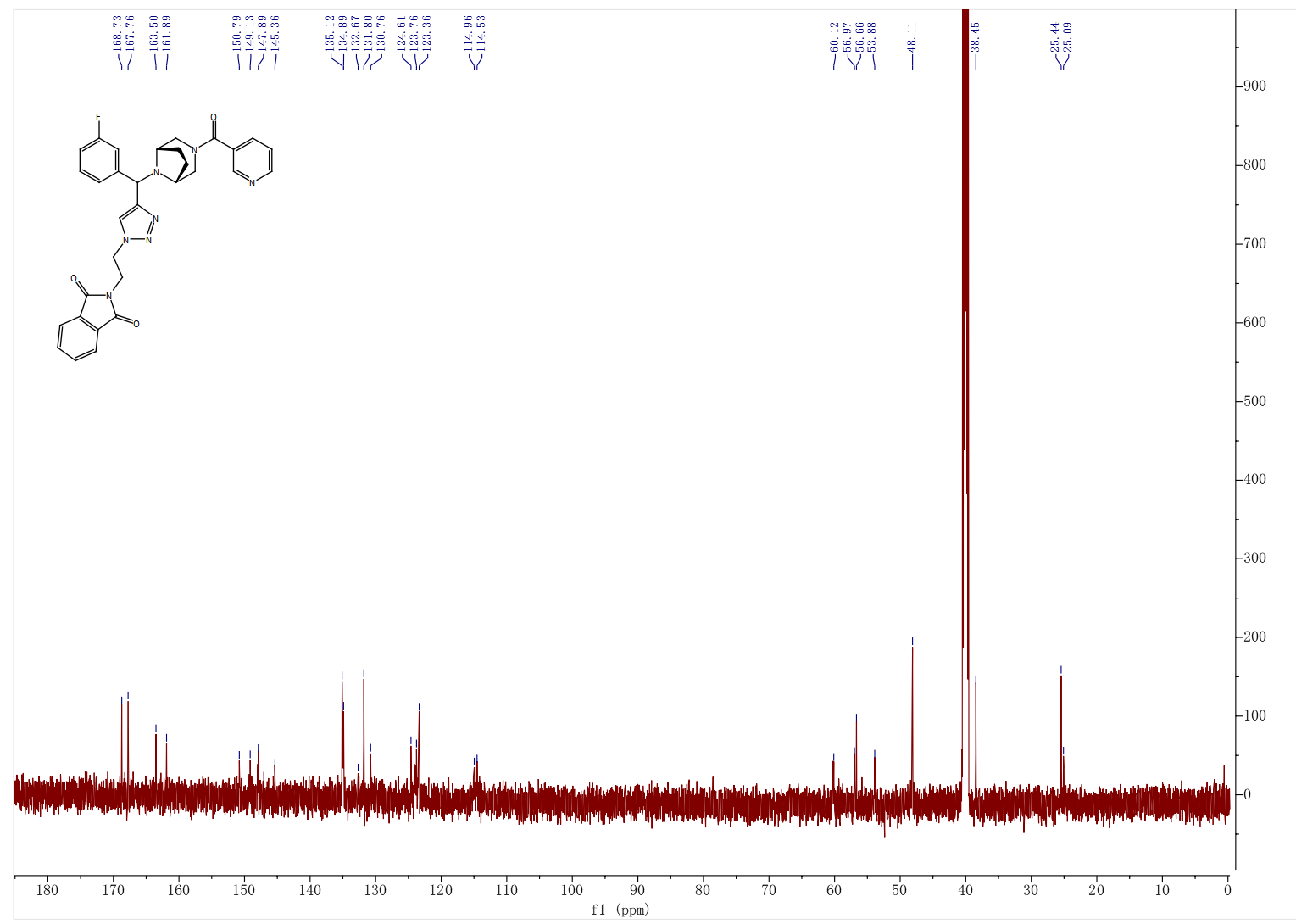


^1^H NMR (600 MHz, CDCl_3_-*d*_6_) of **C5N17A**


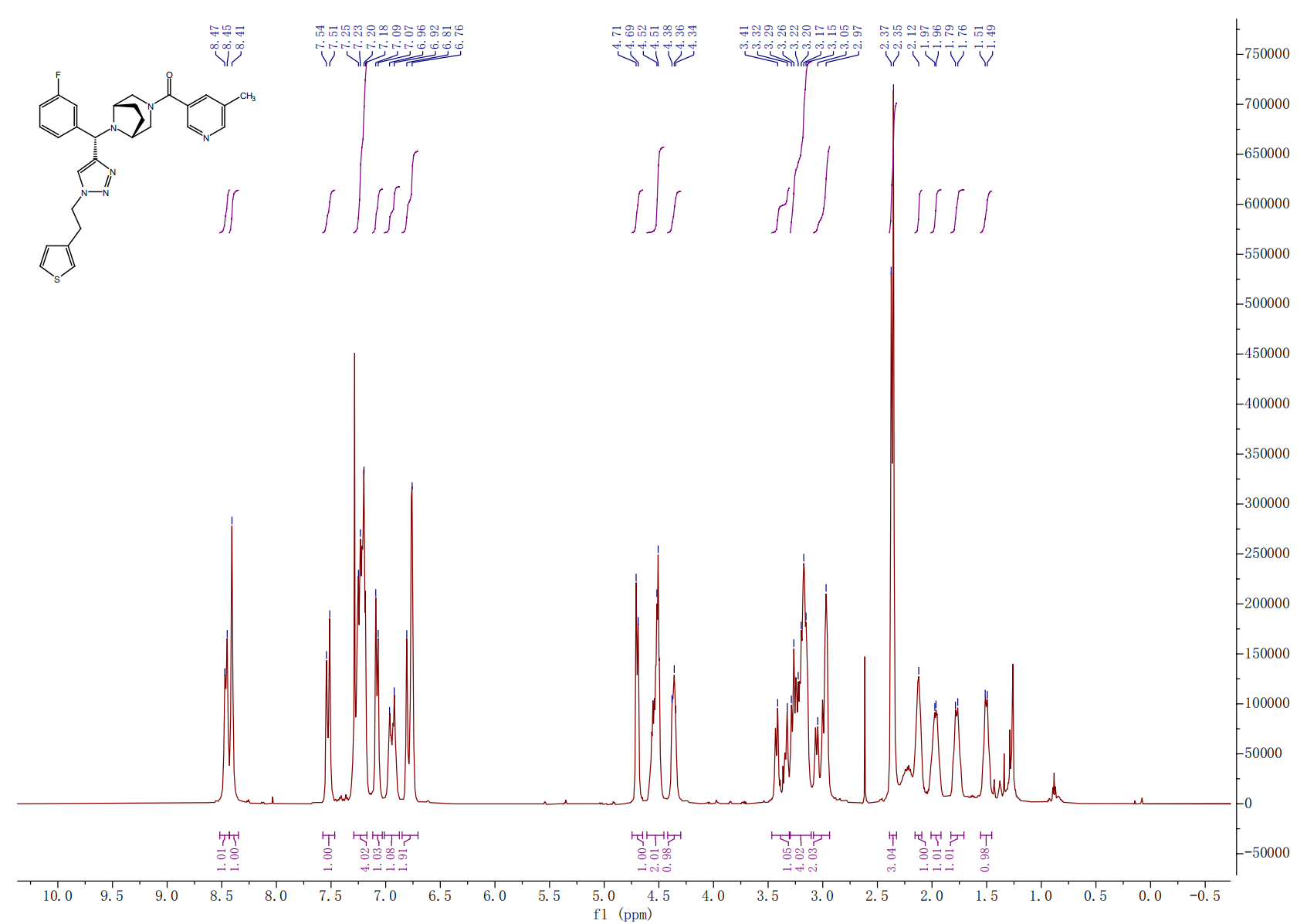


^13^C NMR (150 MHz, CDCl_3_-*d*_6_) of **C5N17A**


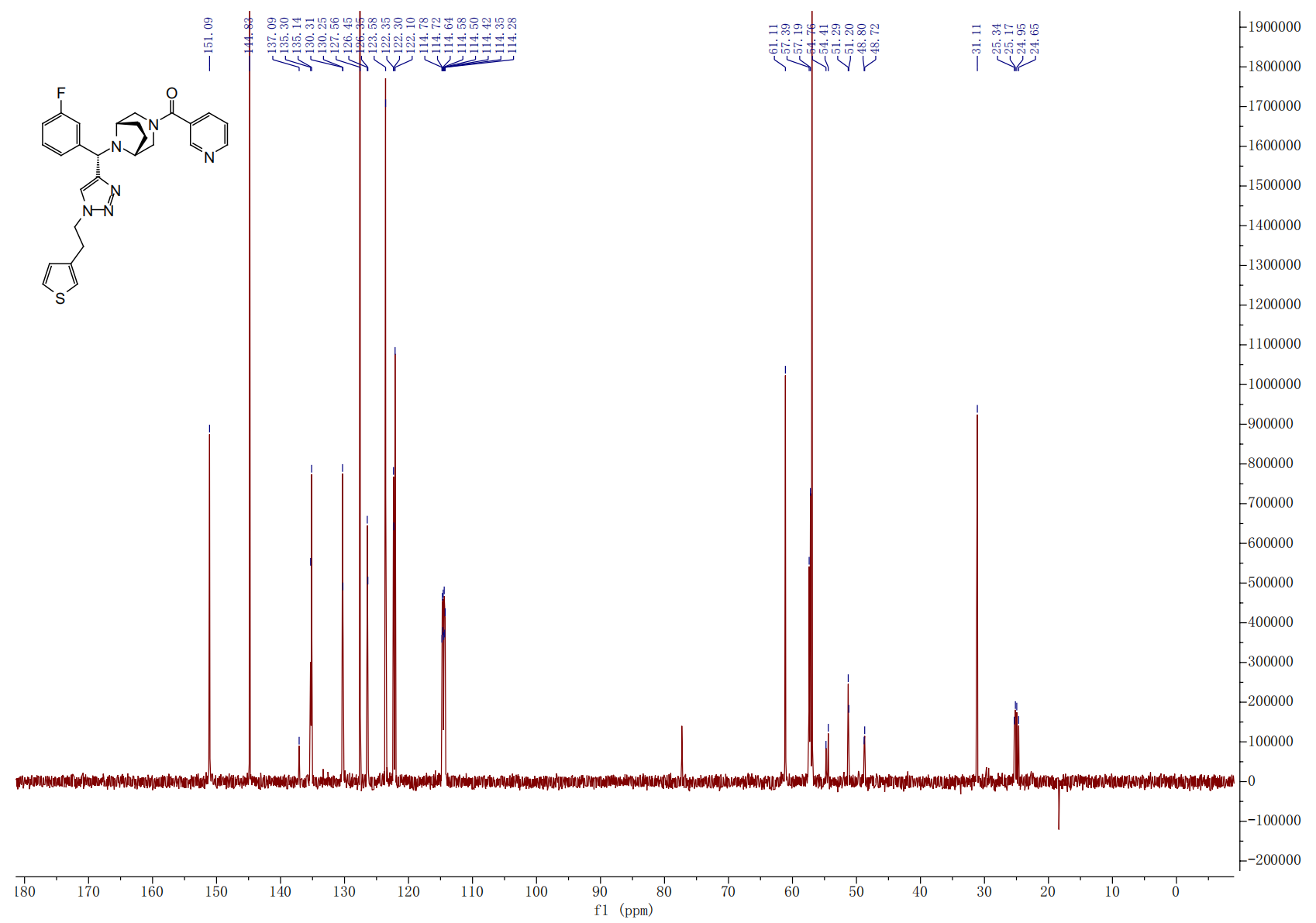


^1^H NMR (600 MHz, CDCl_3_-*d*_6_) of **C5N17B**


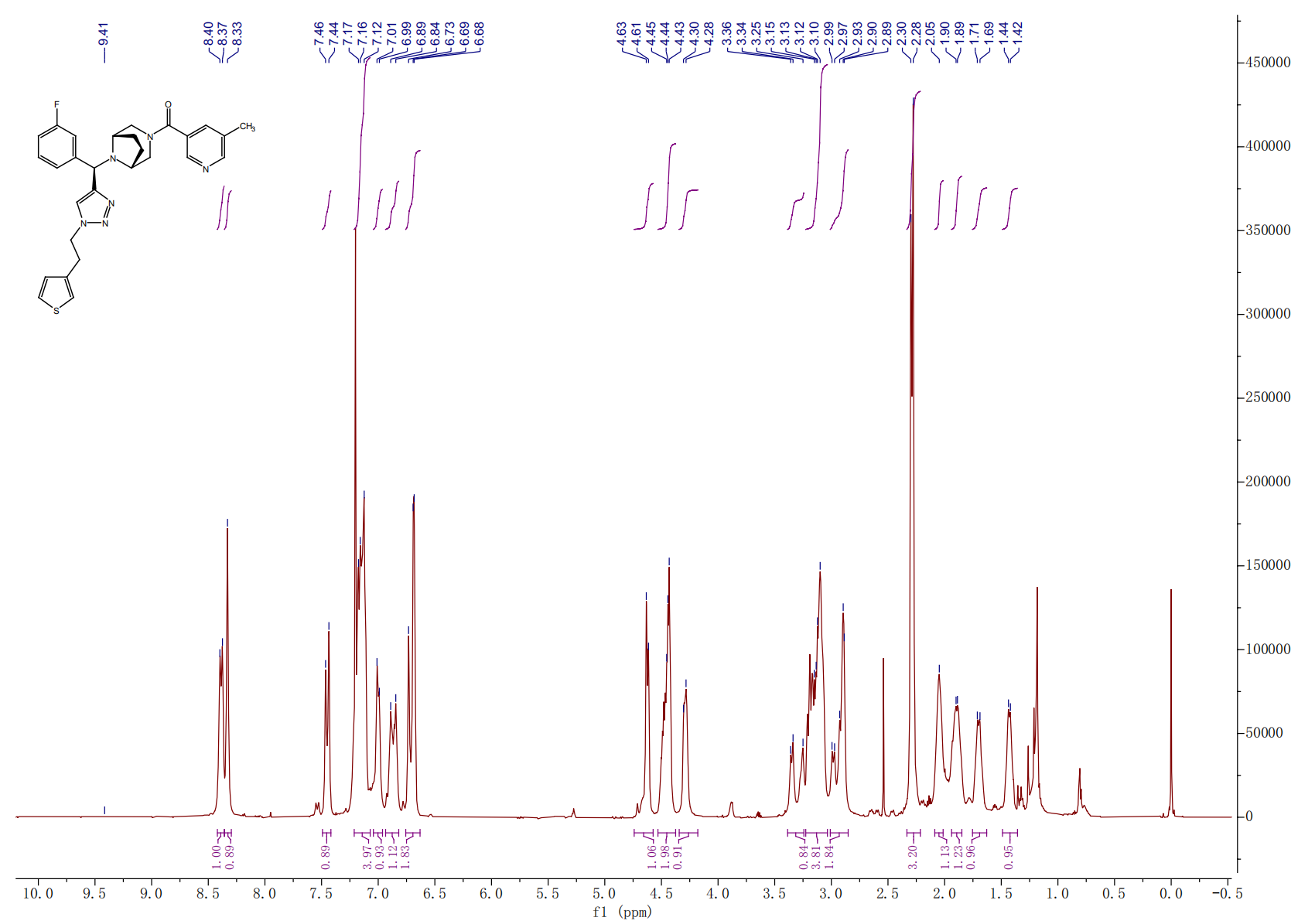


^13^C NMR (150 MHz, CDCl_3_-*d*_6_) of **C5N17B**


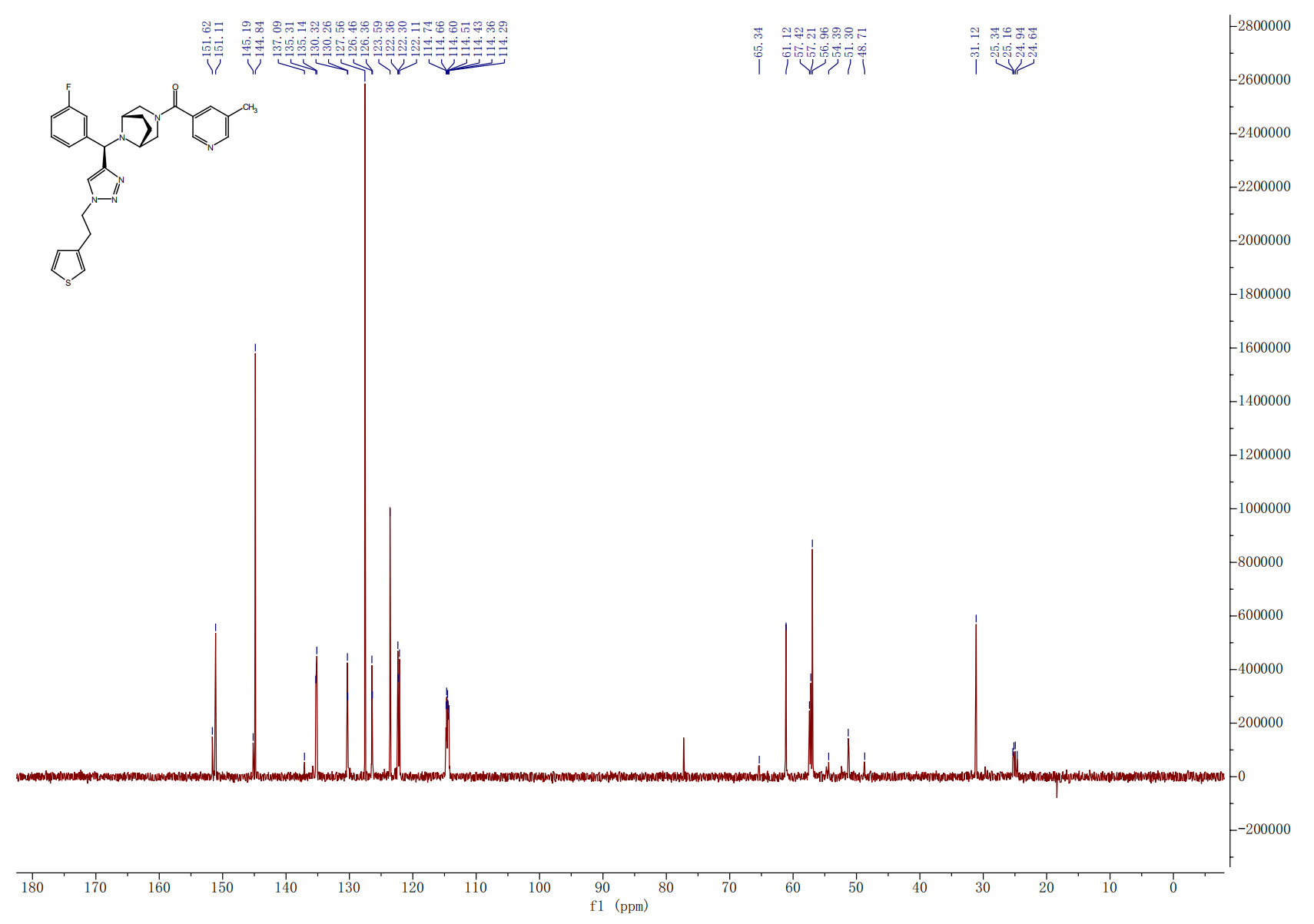


3.4.2 HRMS spectra of final compounds

High-resolution mass spectrometry of **C5N5**

High-resolution mass spectrometry of **C5N6**

High-resolution mass spectrometry of **C5N16**

High-resolution mass spectrometry of **C5N17**

High-resolution mass spectrometry of **C5N21**

High-resolution mass spectrometry of **C5N39**

High-resolution mass spectrometry of **C5N41**

High-resolution mass spectrometry of **C5N42**

High-resolution mass spectrometry of **C5N50**

High-resolution mass spectrometry of **C5N57**

High-resolution mass spectrometry of **C5N58**

High-resolution mass spectrometry of **C5N64**

High-resolution mass spectrometry of **C4N17**

High-resolution mass spectrometry of **C4N21**

High-resolution mass spectrometry of **C6N17**

High-resolution mass spectrometry of **C6N21**

3.4.3 HPLC chromatograms of final compounds

HPLC chromatogram report of **C5N5**


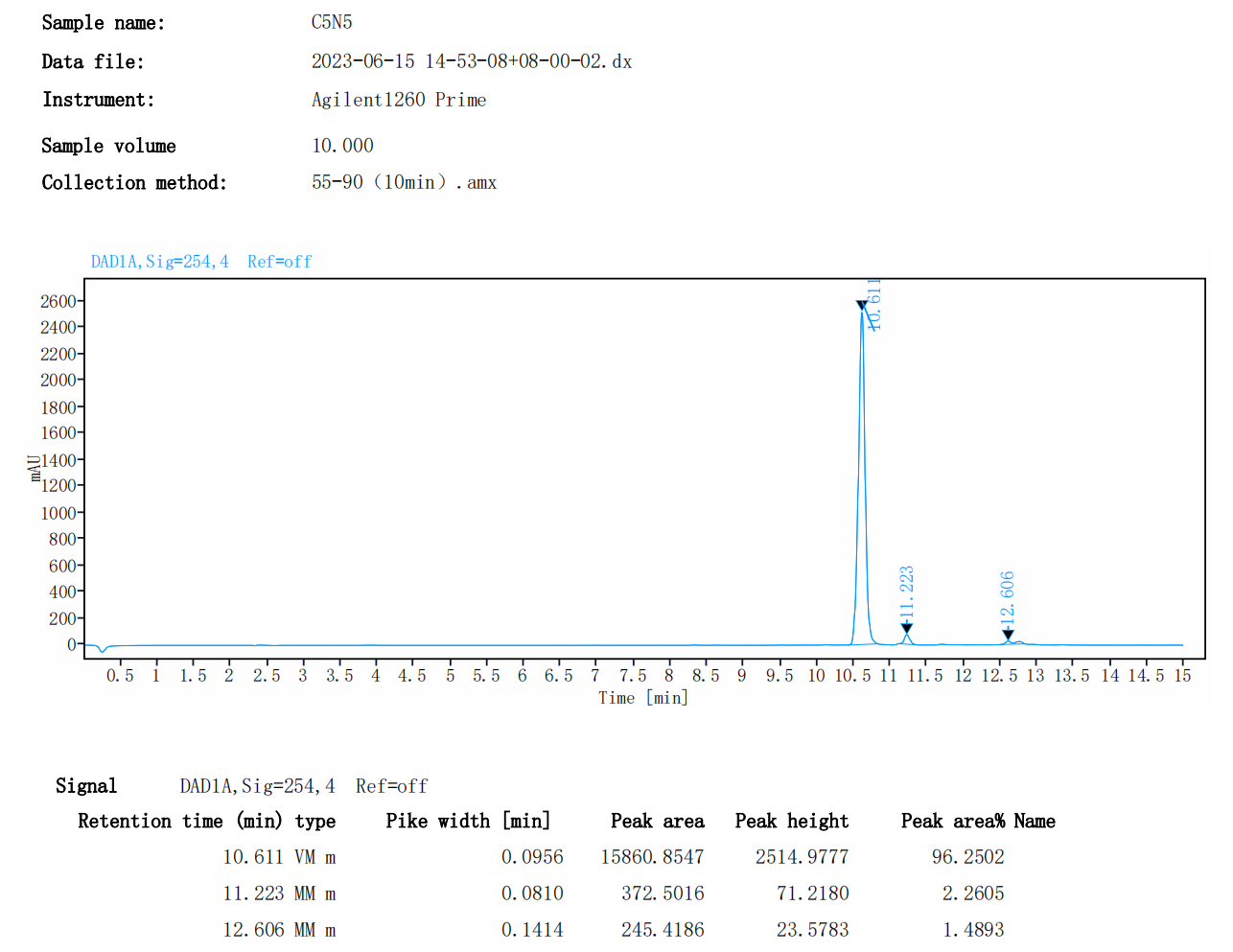


HPLC chromatogram report of **C5N6**


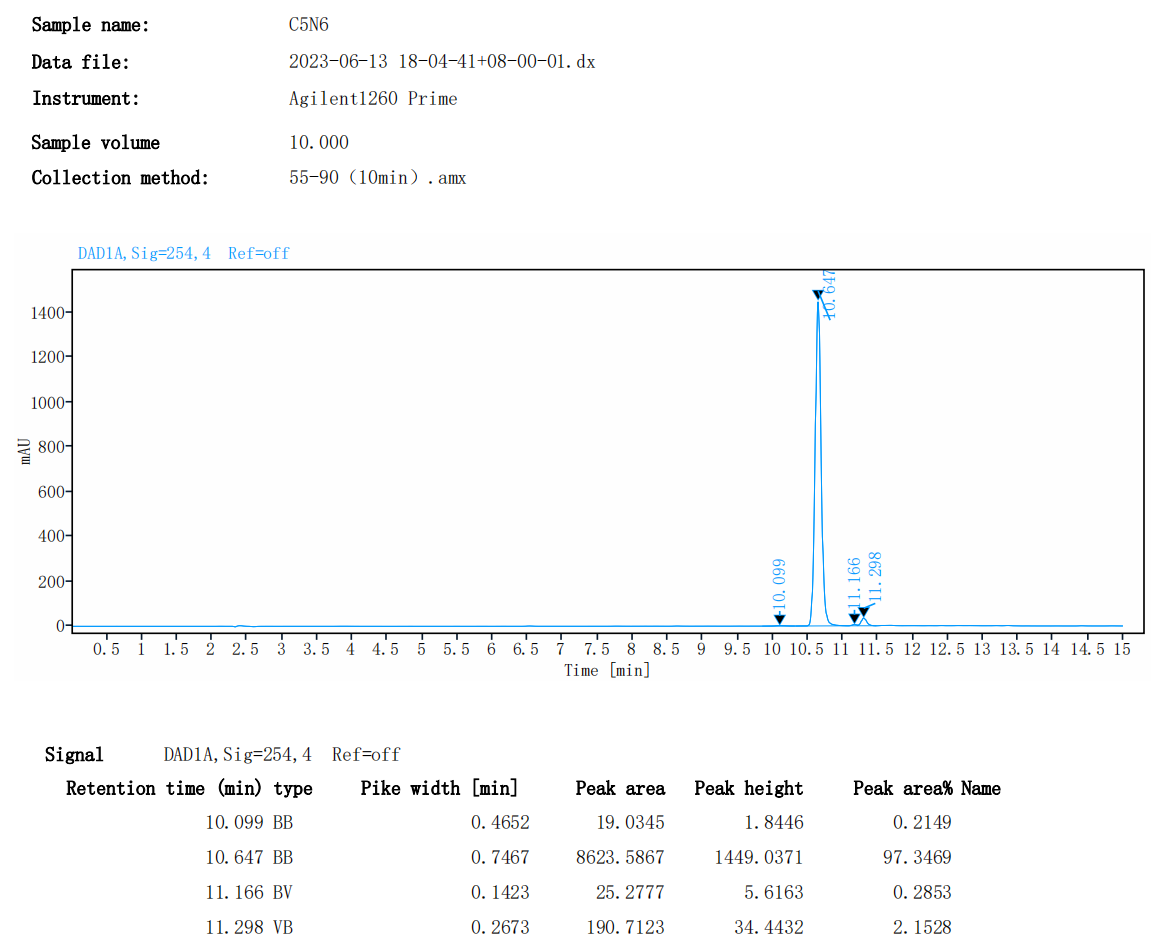


HPLC chromatogram report of **C5N16**


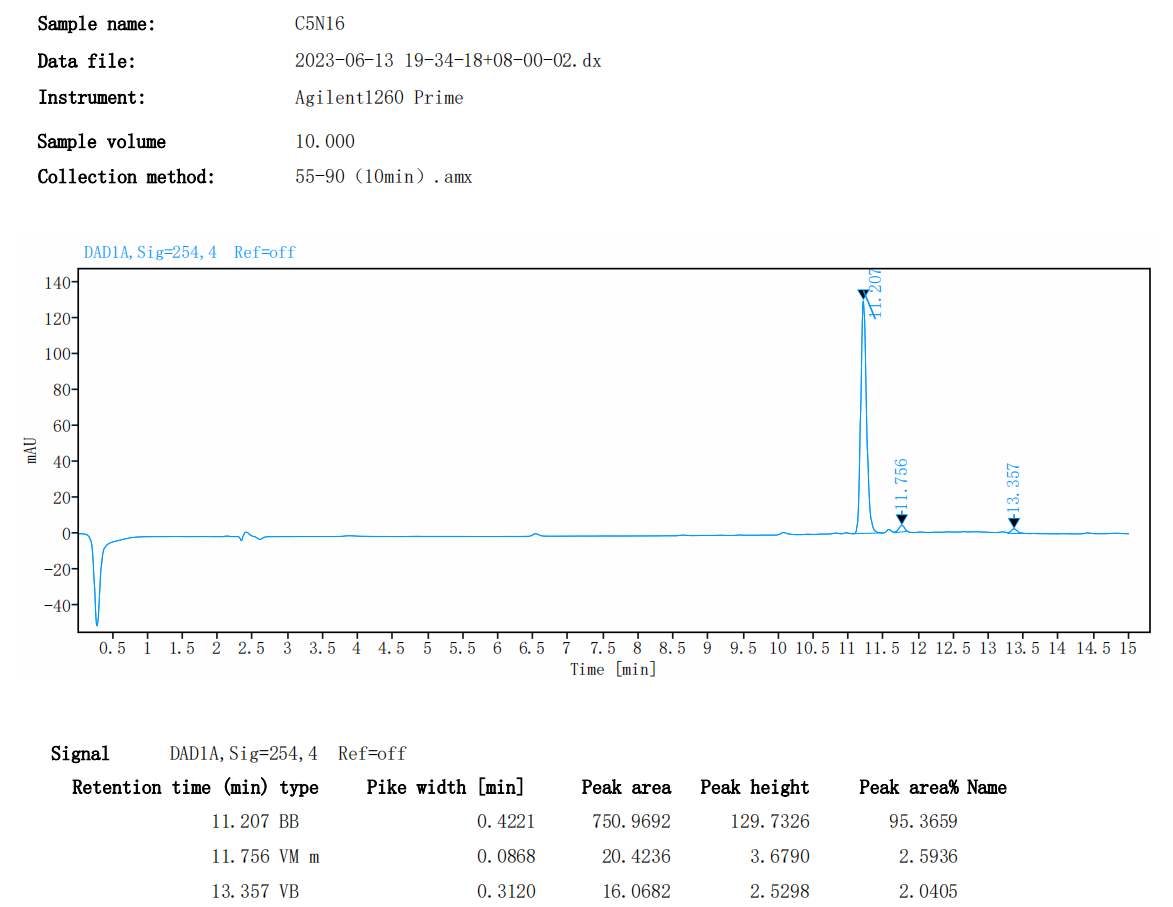


HPLC chromatogram report of **C5N17**


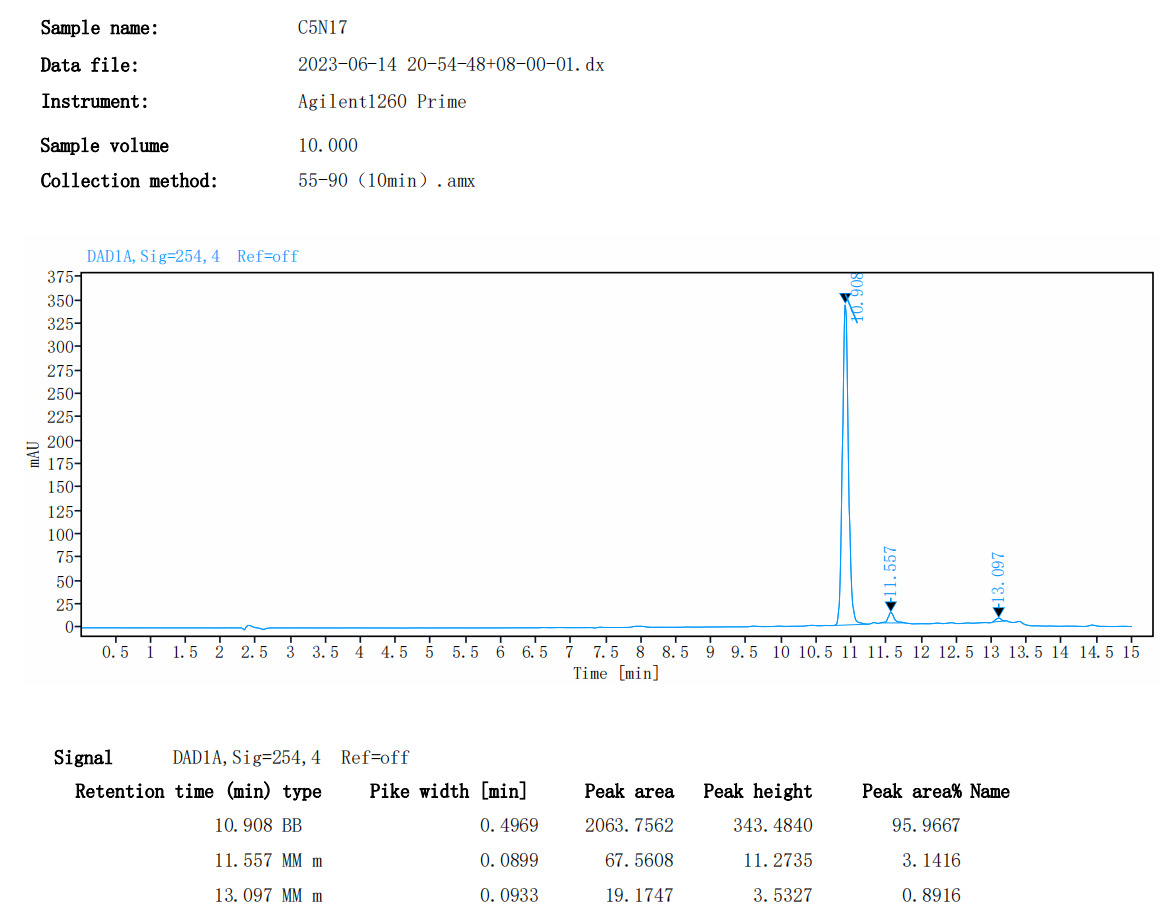


HPLC chromatogram report of **C5N21**


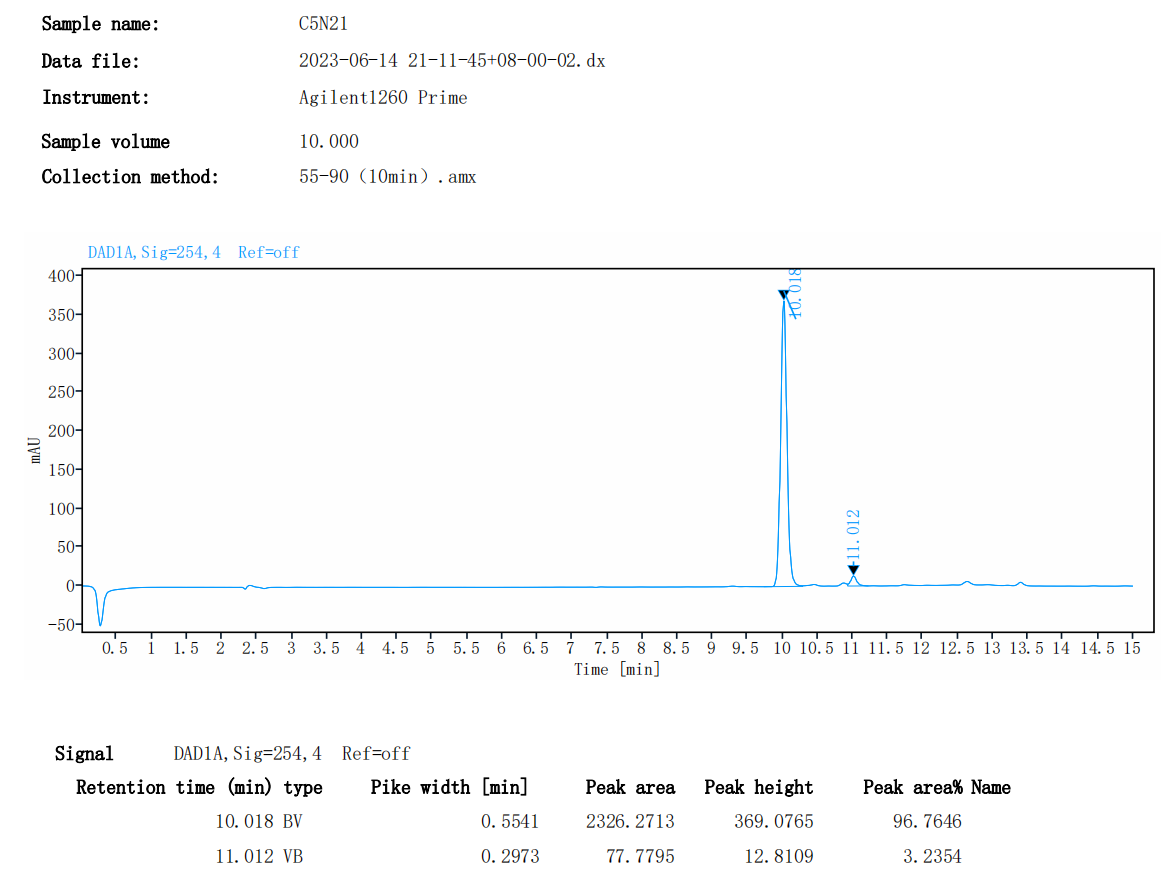


HPLC chromatogram report of **C5N39**


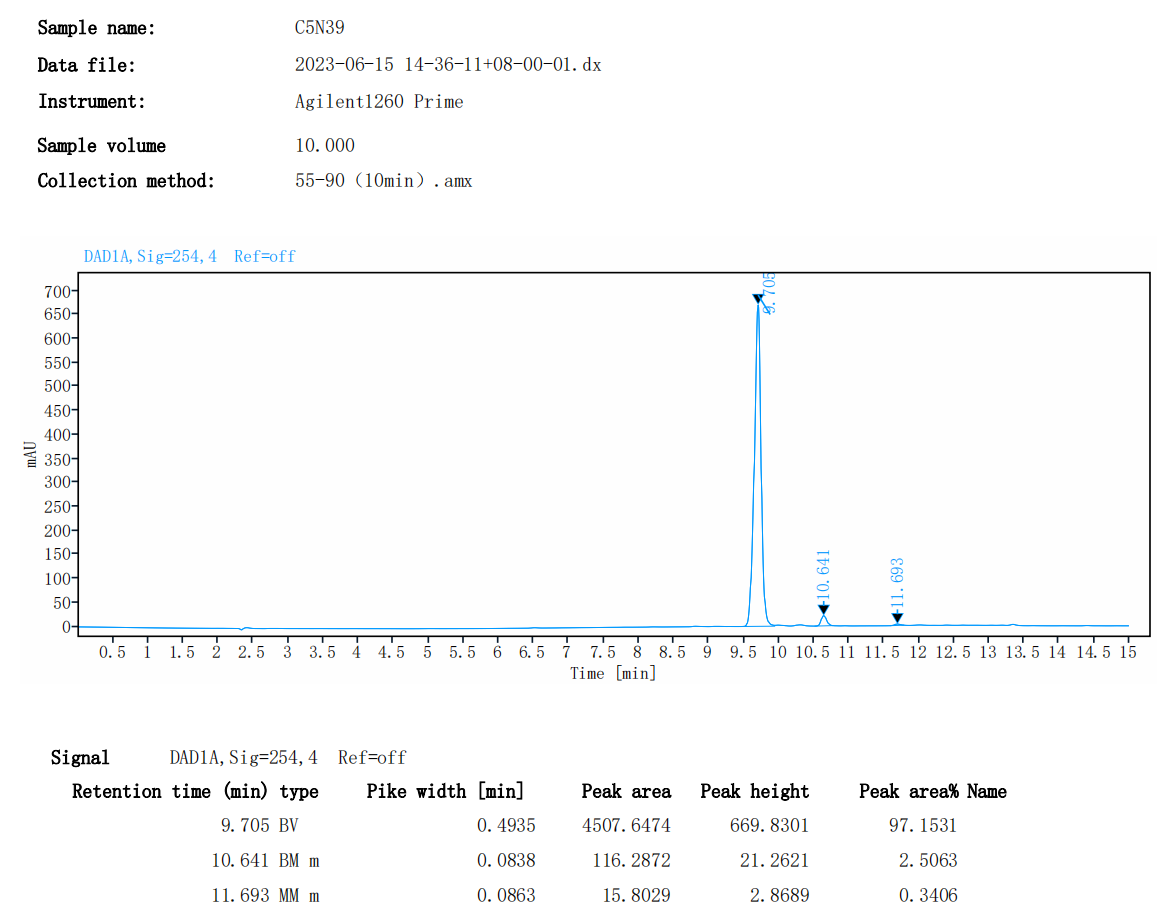


HPLC chromatogram report of **C5N41**


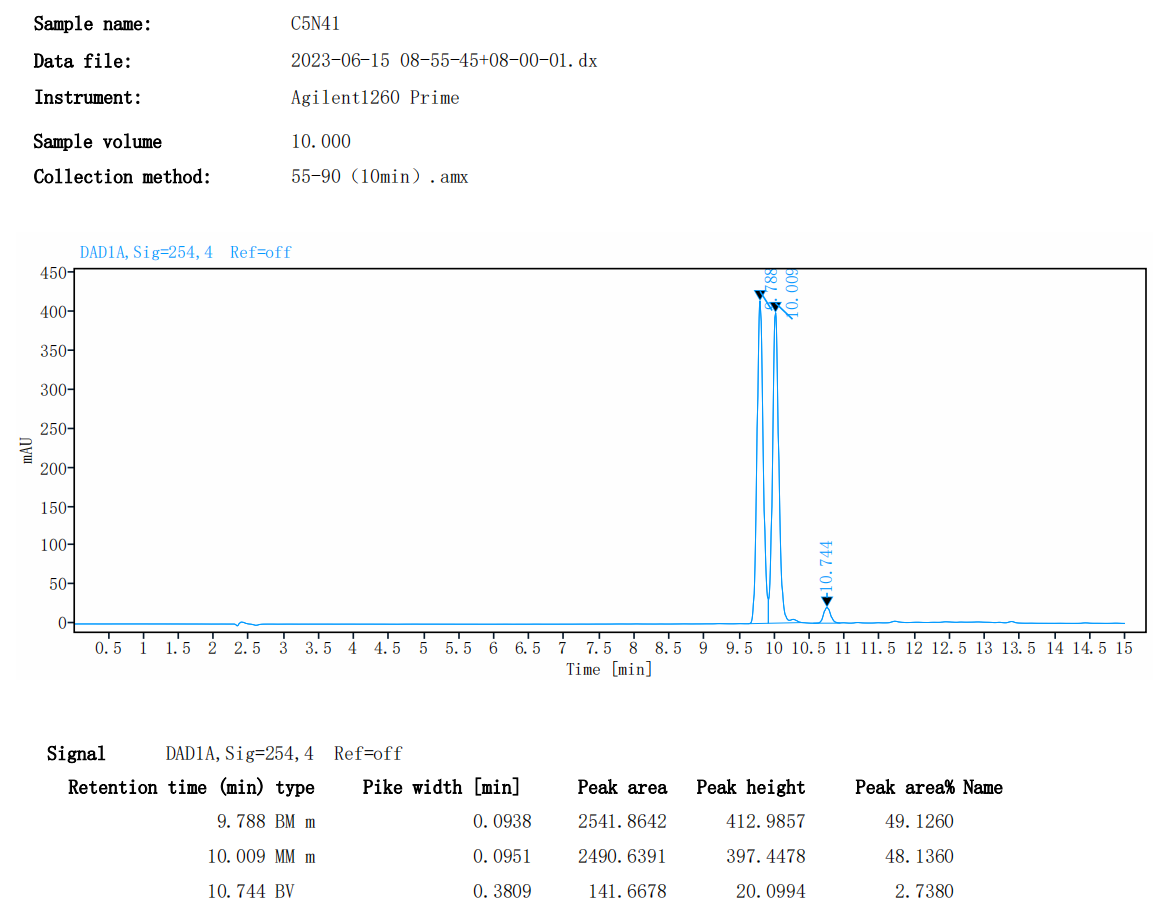


HPLC chromatogram report of **C5N42**


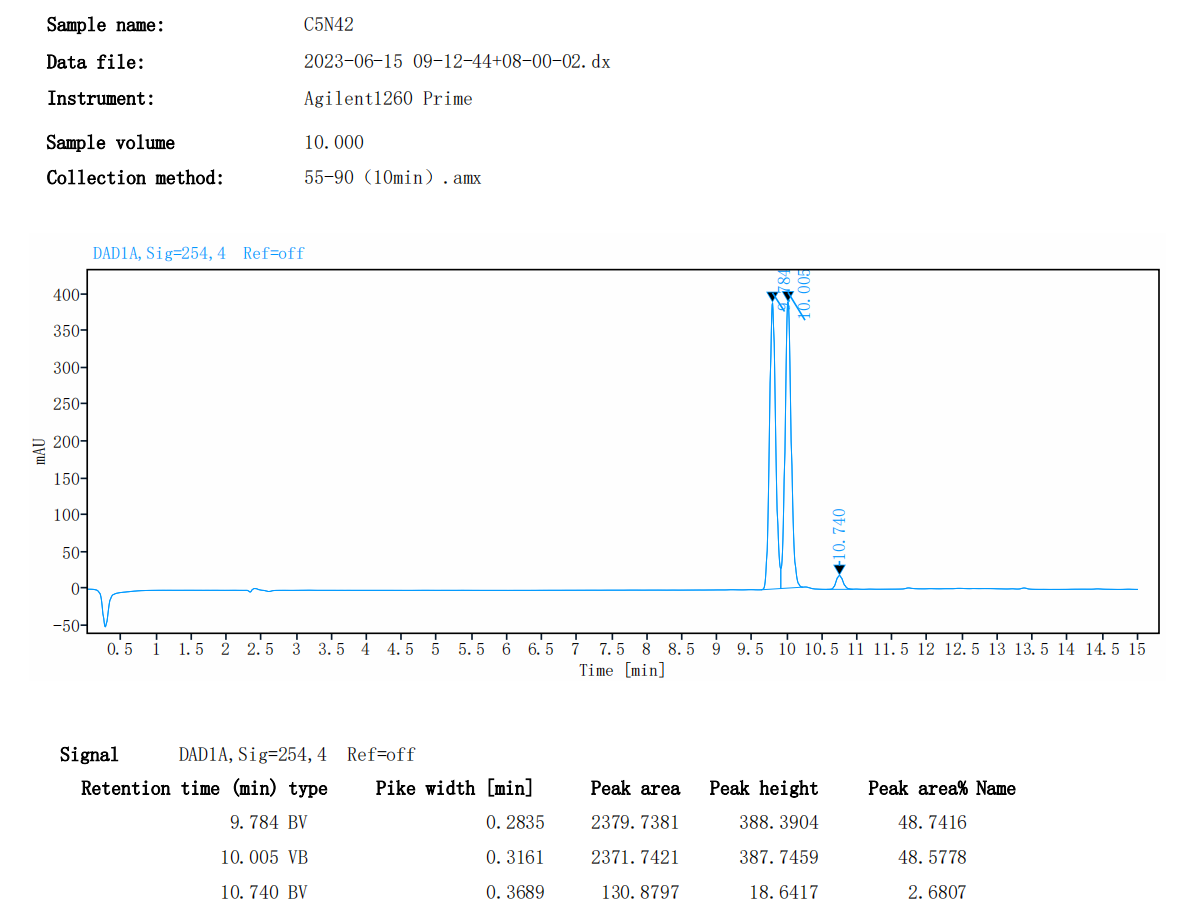


HPLC chromatogram report of **C5N50**


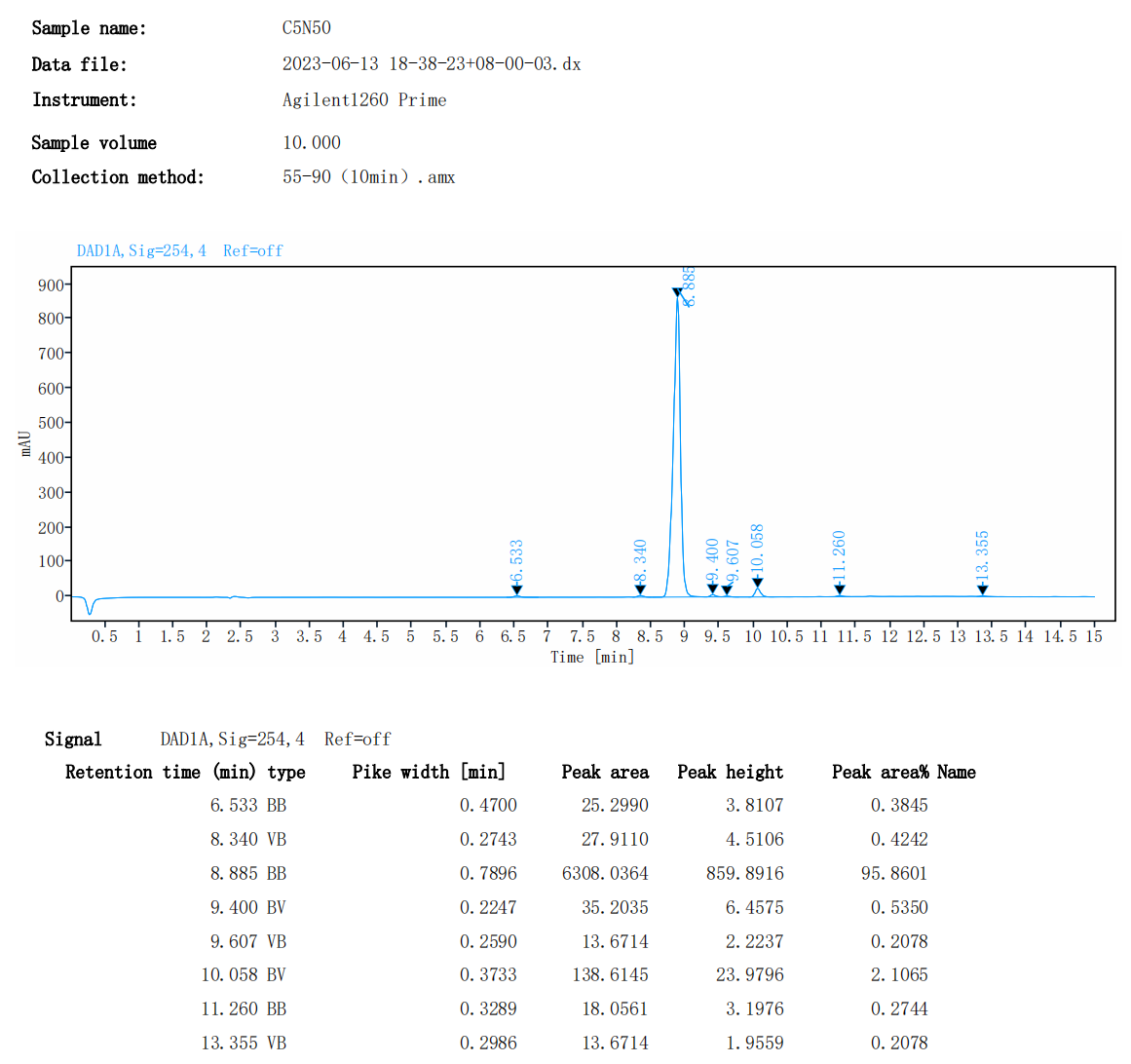


HPLC chromatogram report of **C5N57**


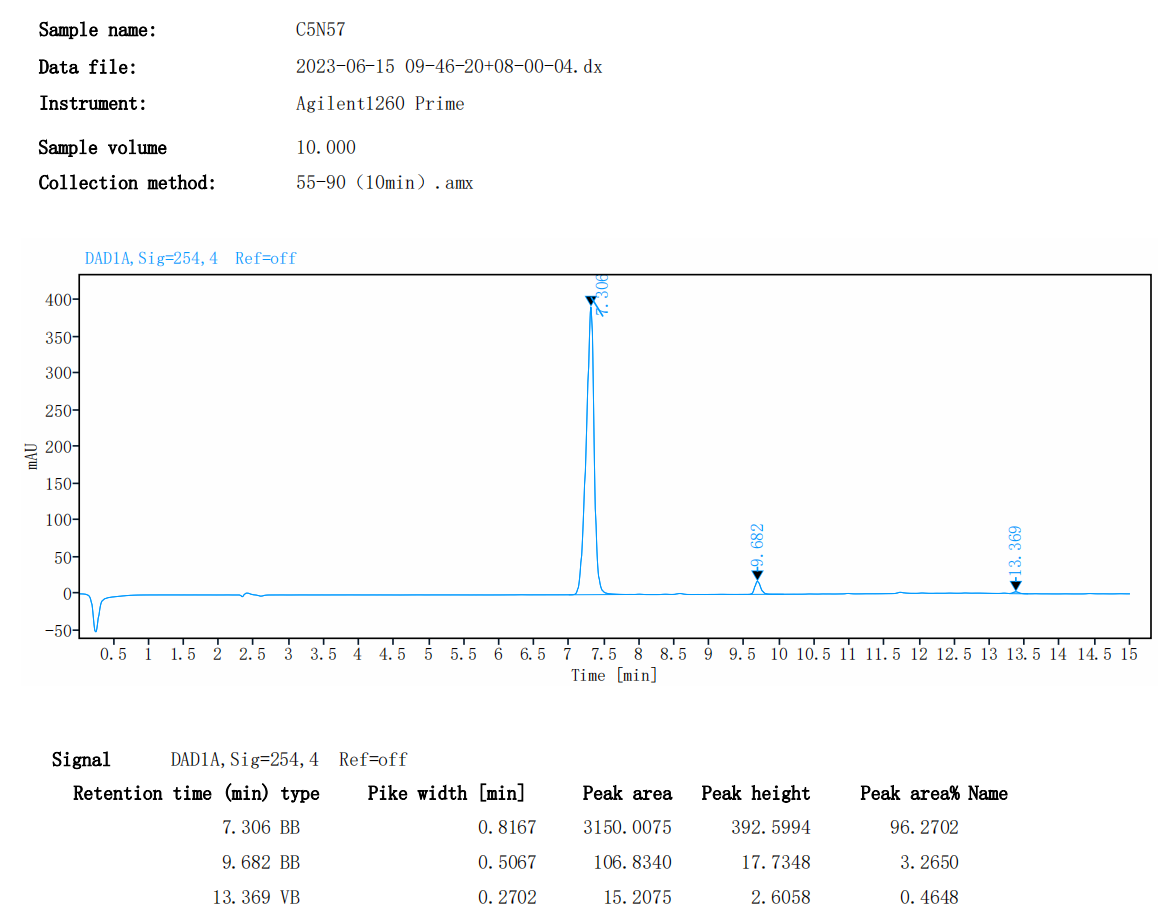


HPLC chromatogram report of **C5N58**


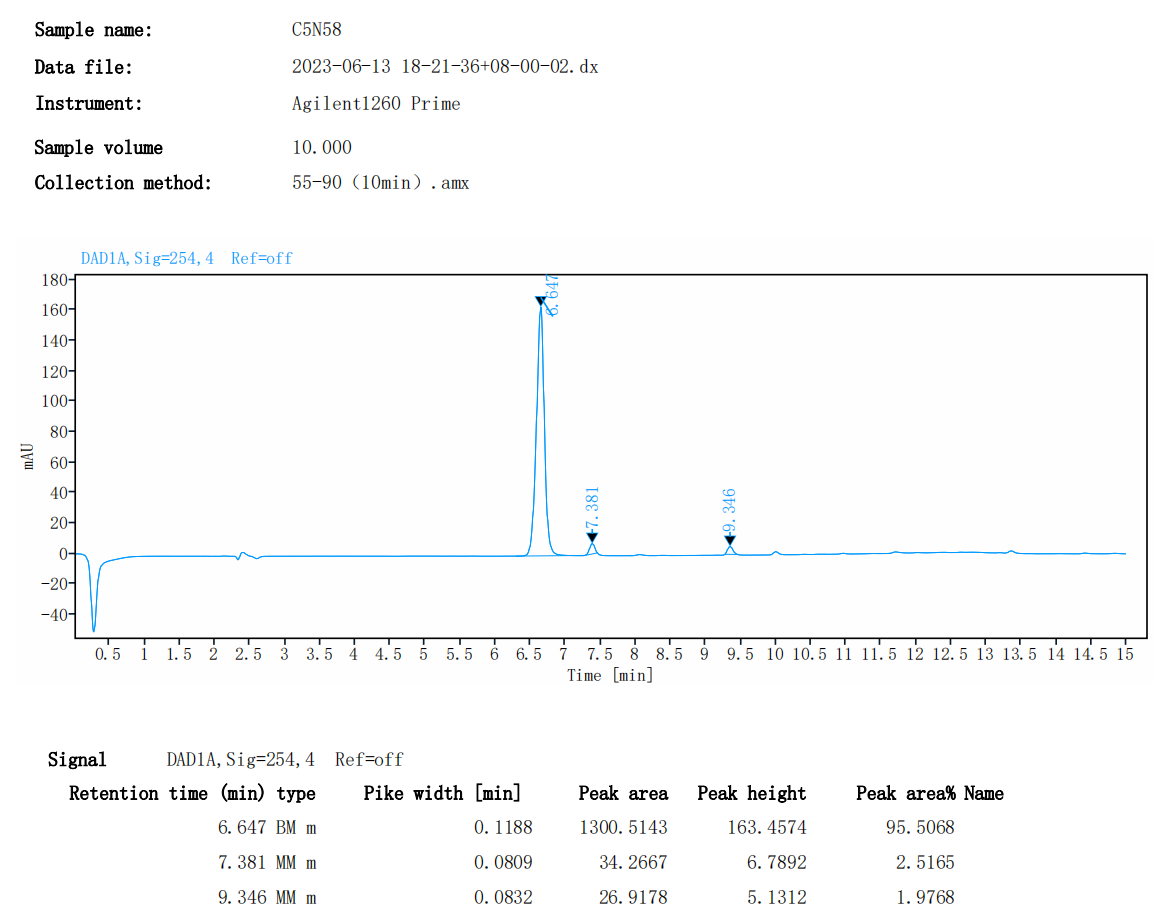


HPLC chromatogram report of **C5N64**


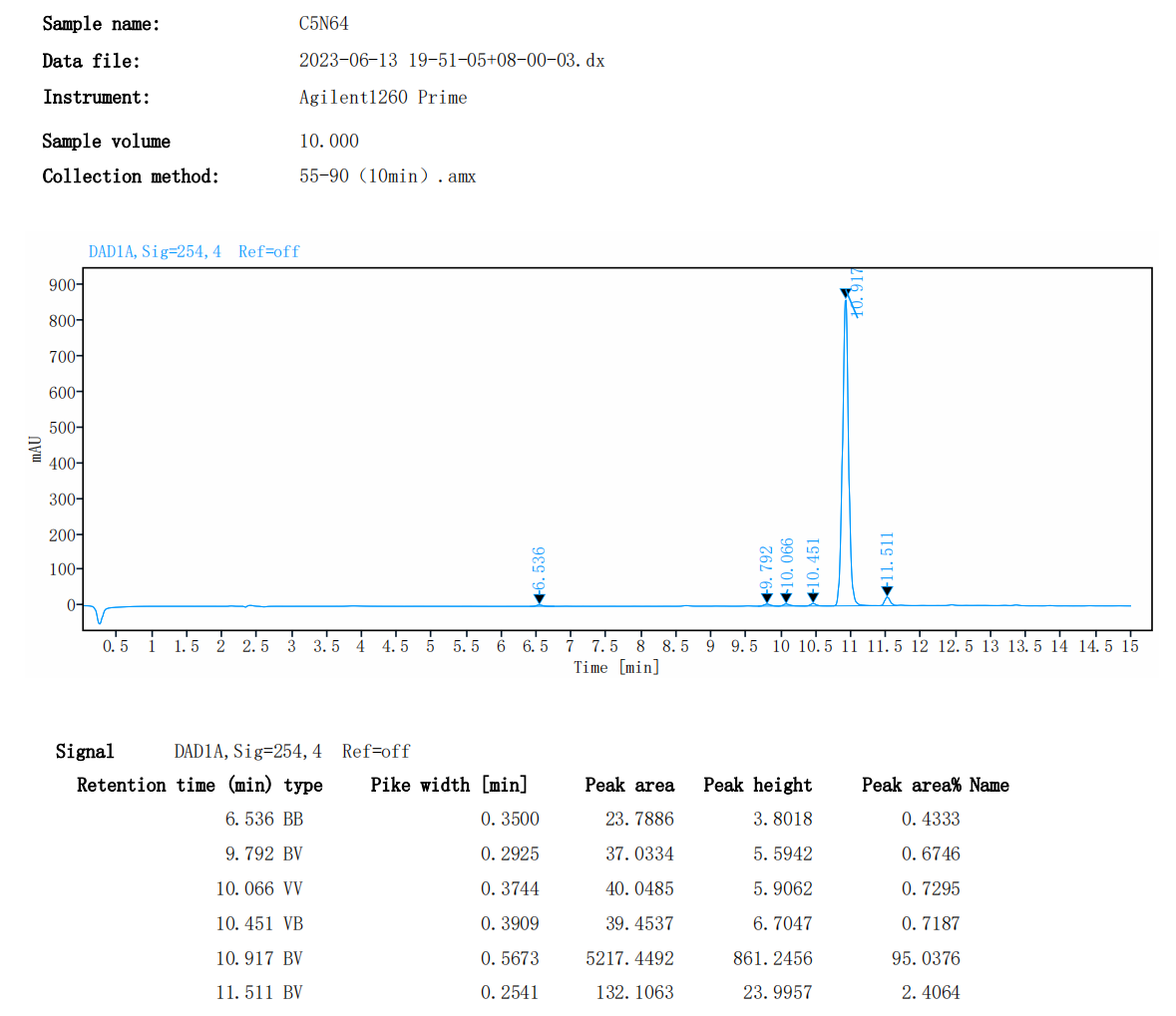


HPLC chromatogram report of **C6N17**


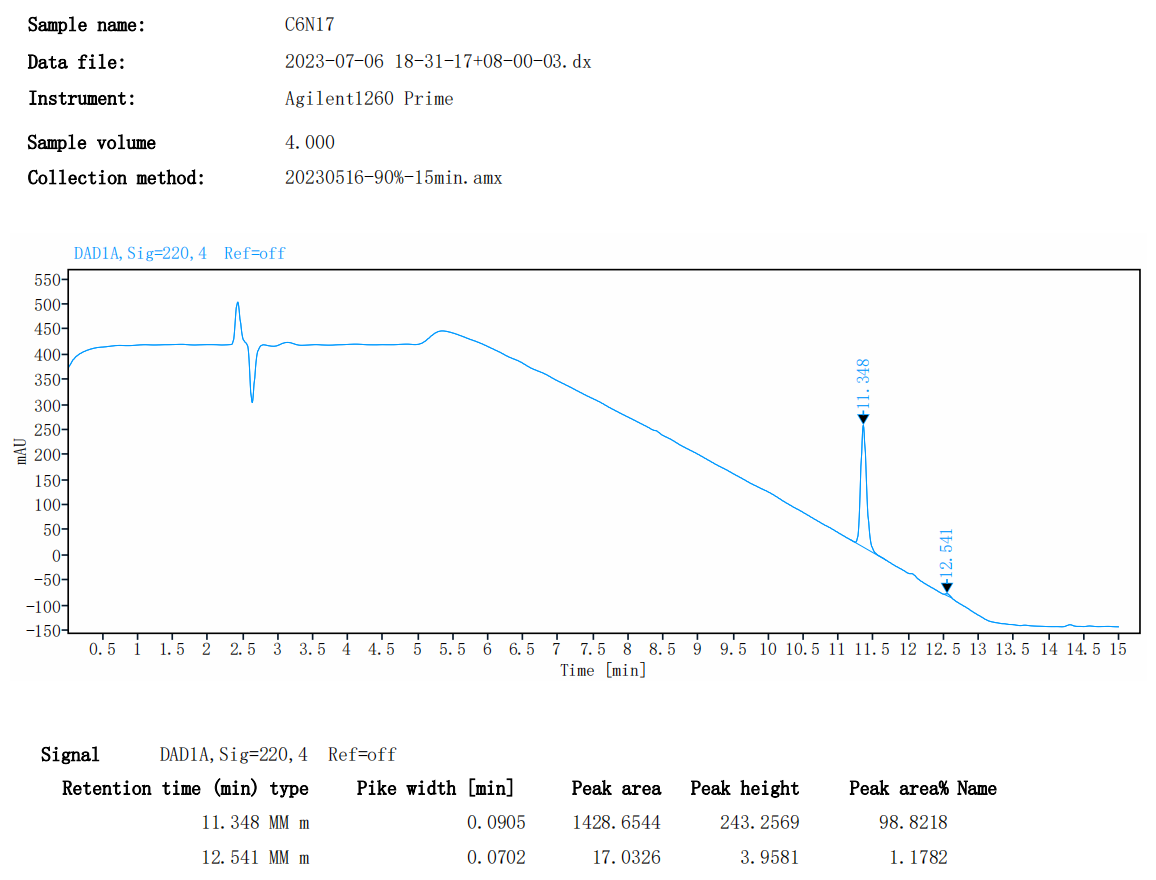


HPLC chromatogram report of **C6N21**


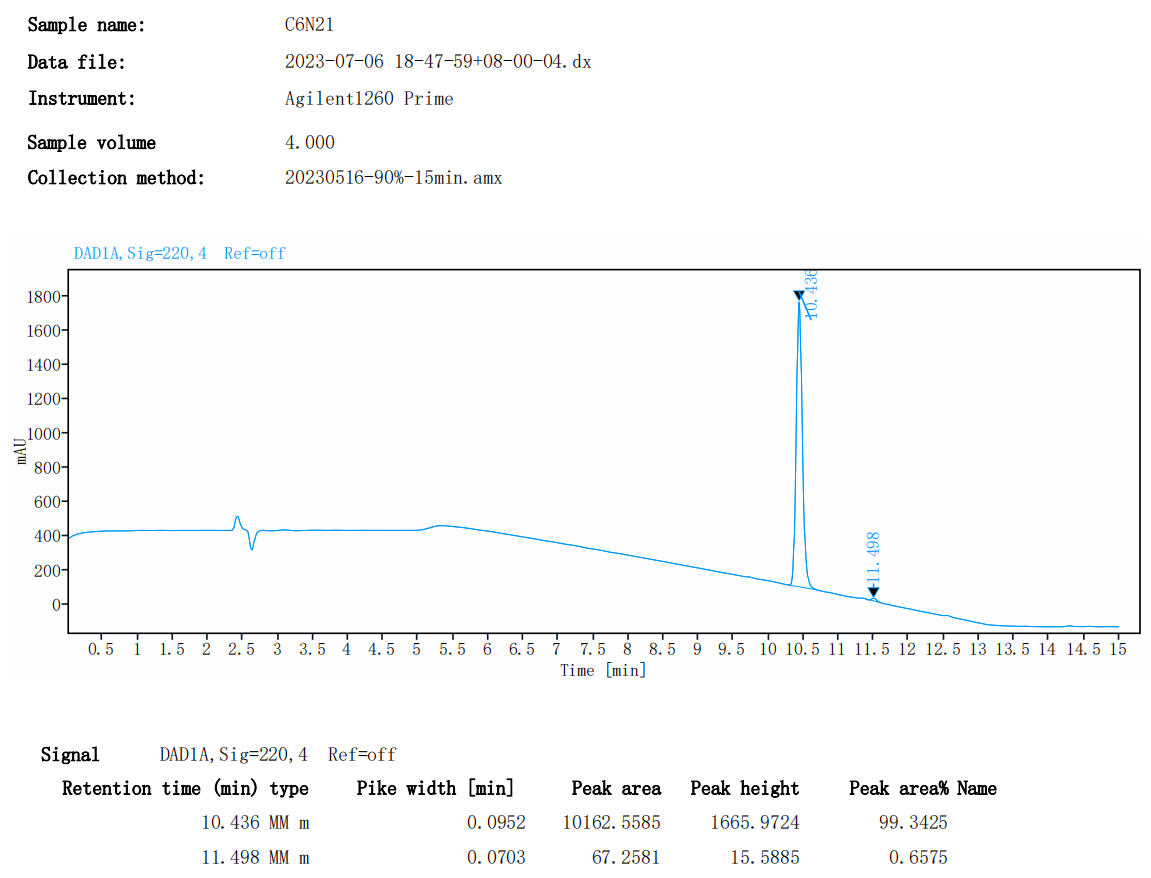


HPLC chromatogram report of **C4N17**


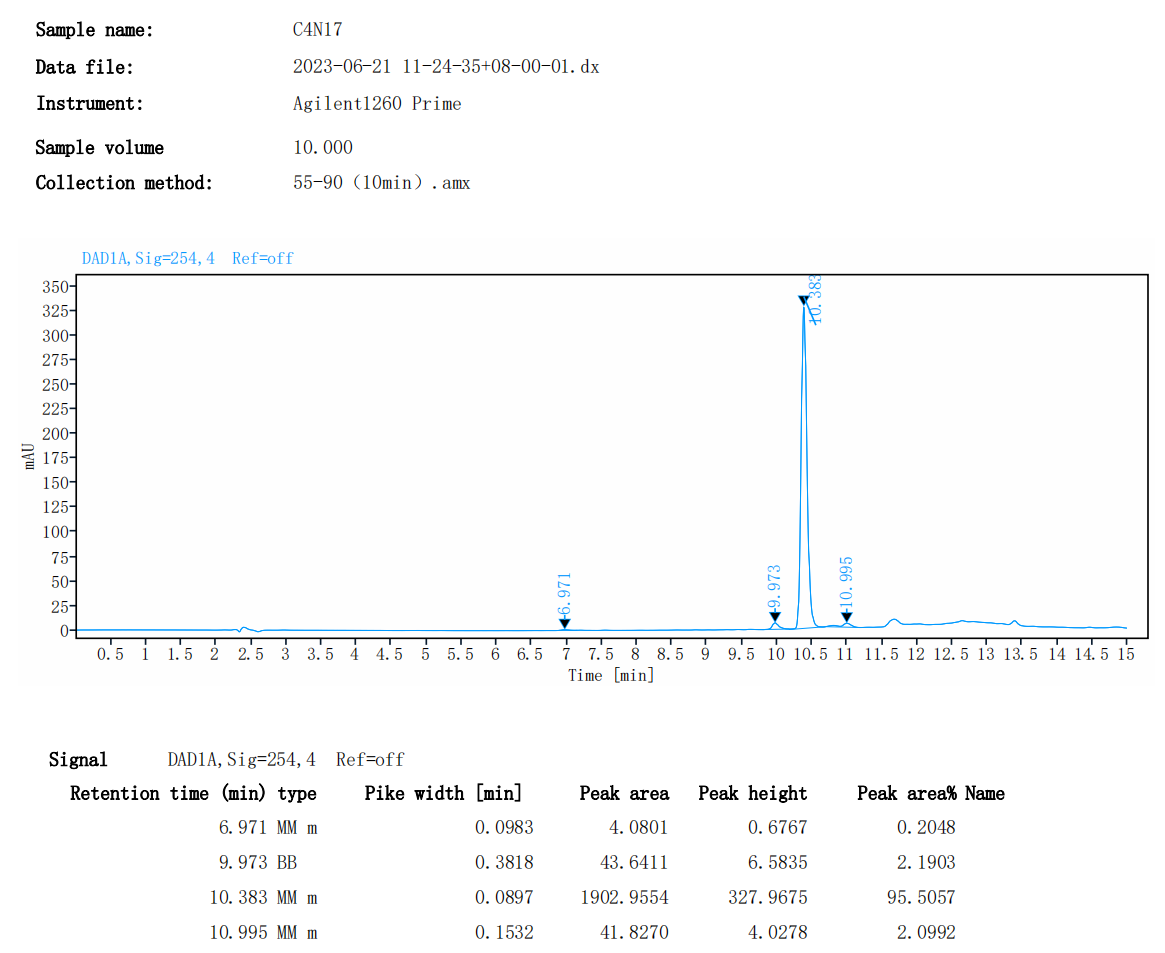


HPLC chromatogram report of **C4N21**


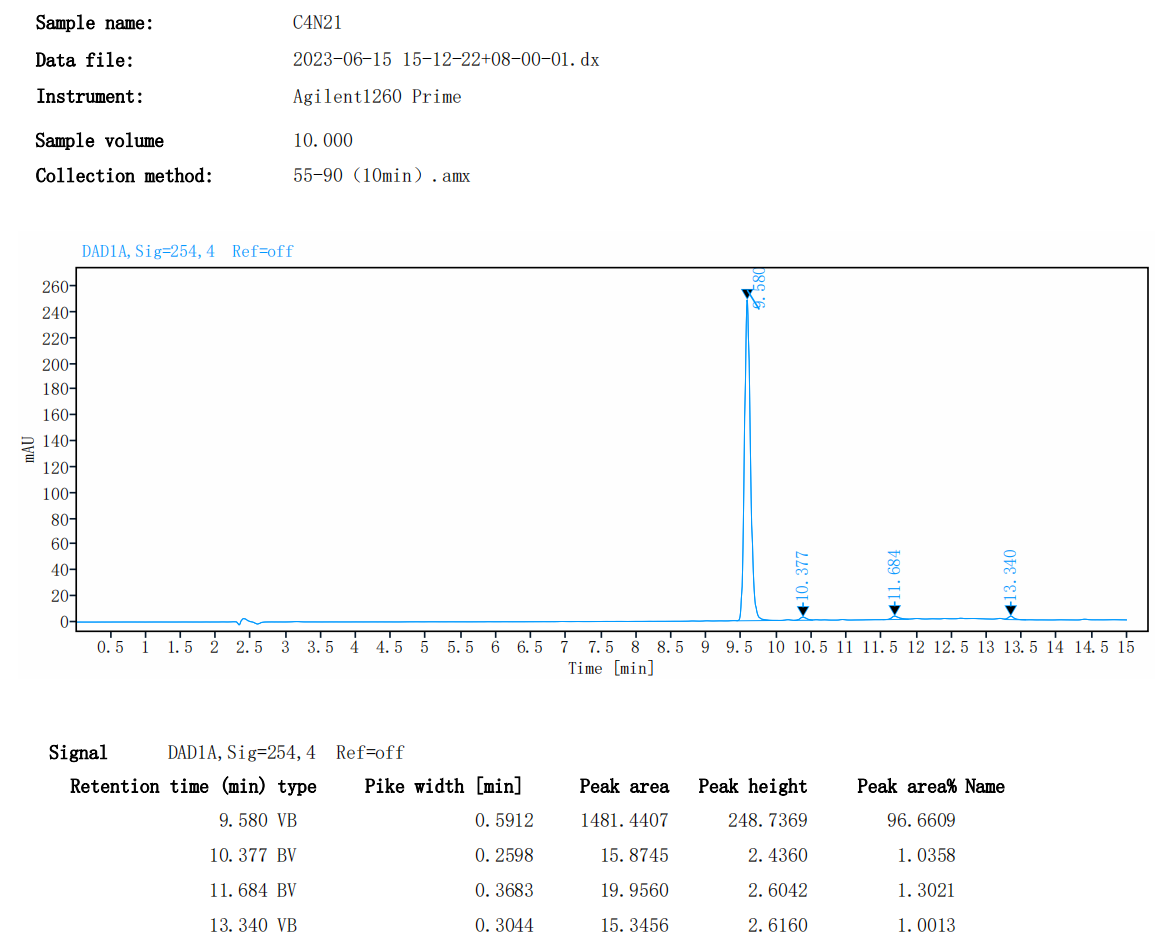


Chiral HPLC chromatogram report of **C5N17A**


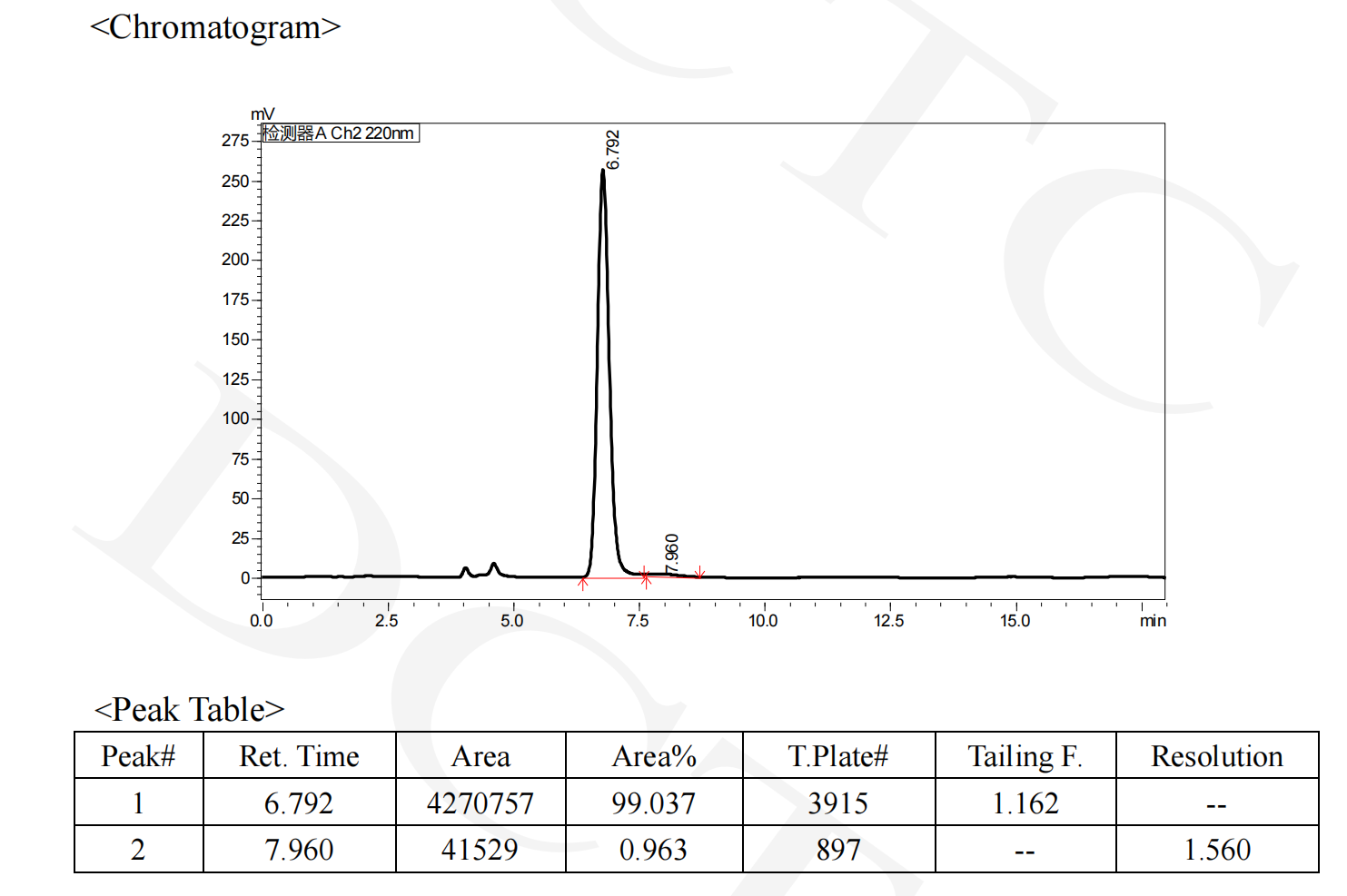


Chiral HPLC chromatogram report of **C5N17B**


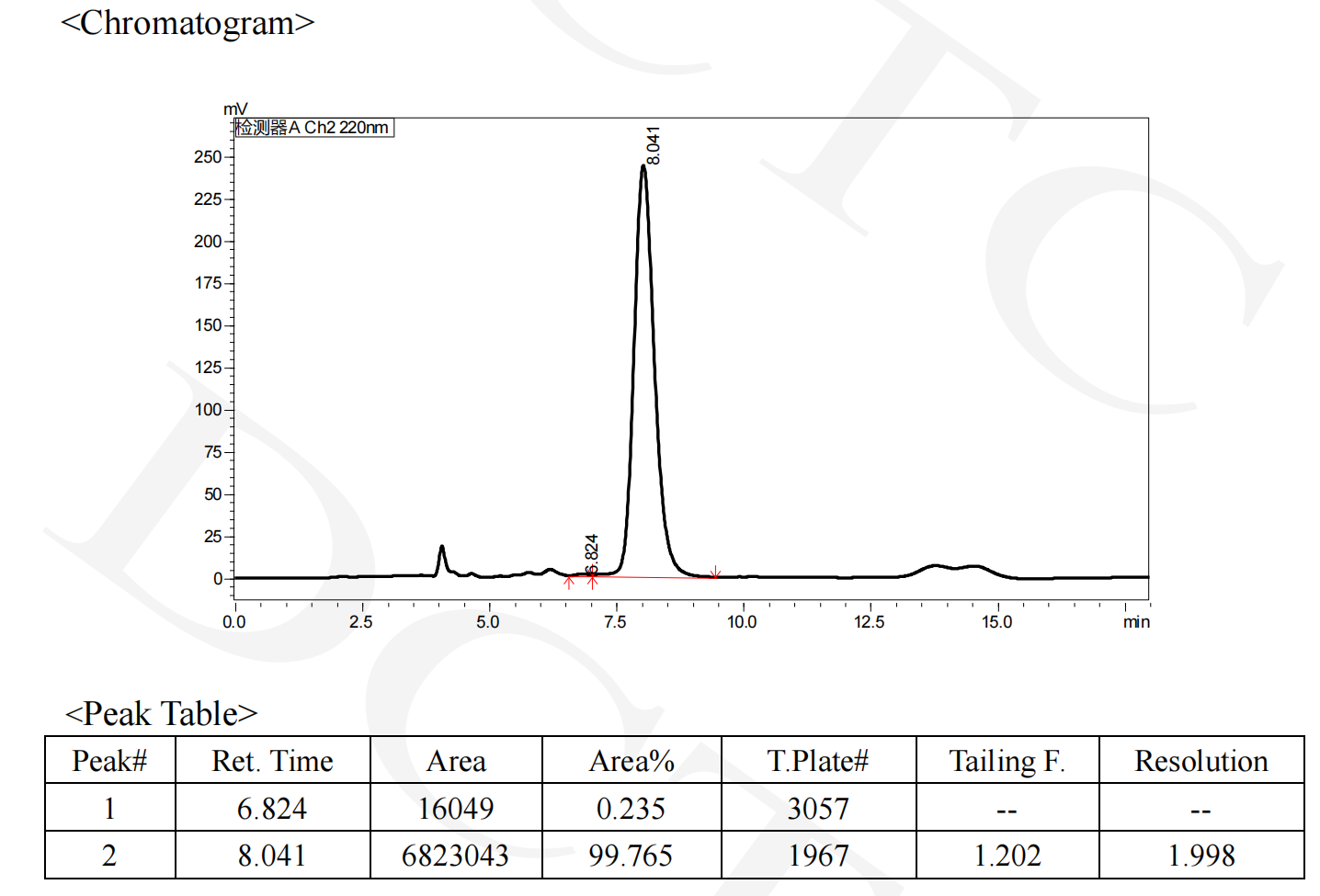

Supplement: Supplementary file 1 — Supporting Information [file ADVS-11-2404884-s001.docx]
